# G‑Quadruplex-Interacting Photooxidative Platinum(IV) Prodrug for Inhibiting Oncogene Transcription and Amplifying Antitumor Immunity

**DOI:** 10.1021/jacs.6c16762

**Published:** 2026-10-21

**Authors:** Dengshuai Wei, Yijia Chen, Jiaqian Xu, Guangyu Zhu

**Affiliations:** † Department of Chemistry, 53025 City University of Hong Kong, Kowloon Tong, Hong Kong SAR 999077, P. R. China; ‡ Department of Pharmaceutics, School of Pharmacy, 12593 Qingdao University, Qingdao 266021, P. R. China; § City University of Hong Kong Shenzhen Research Institute, Shenzhen 518057, P. R. China

## Abstract

Conventional platinum (Pt)-based chemotherapeutics elicit cancer cell death primarily through DNA binding, but this approach is inherently limited by poor site specificity and a lack of spatiotemporal control, which compromises therapeutic precision and safety. To overcome these limitations, we report DNATplatin, an *in situ* activatable and photooxidative Pt­(IV) prodrug designed to combine precise recognition of DNA secondary structures with targeted genetic intervention through DNA photooxidation. By incorporating a bis-*N*-methylpyridinium moiety to selectively recognize DNA, DNATplatin shows a 10-fold increase in the levels in genomic DNA relative to oxaliplatin and a 2.5-fold emission turn-on selectivity for G-quadruplexes (G4s) over typical duplex DNA. Following precise nuclear localization, photoactivated DNATplatin acts as a potent photooxidant that oxidizes G4, dismantles G4 scaffolds, and abrogates circular dichroism signatures. These orchestrated lesions generate localized oxidative stress and selectively silence major G4-driven oncogenes, thereby effectively circumventing traditional Pt resistance by bypassing canonical adduct-dependent pathways. In a murine triple-negative breast tumor model, DNATplatin produces profound tumor suppression and elicits a robust systemic immune response, characterized by CD4^+^/CD8^+^ T-cell activation and depletion of Foxp3^+^ regulatory T cells. Collectively, this work establishes a controllable “target-and-destroy” strategy and underscores the potential of Pt­(IV) photooxidants for topology-selective genetic intervention.

## Graphical abstract

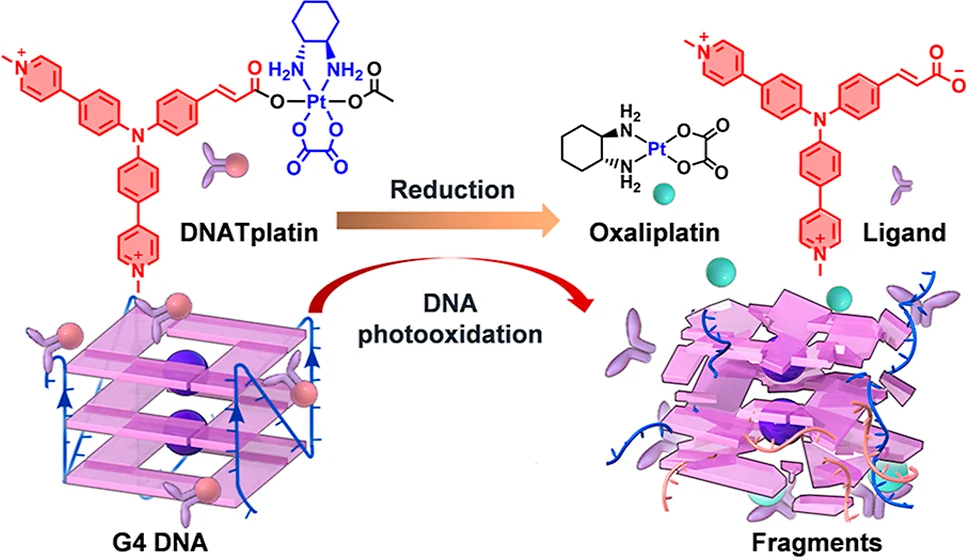

## Introduction

DNA in all organisms encodes the critical information required for sustaining life, regulating fundamental cellular processes such as proliferation, differentiation, and apoptosis.^1^ Structural or functional aberrations in DNA can cause cell cycle dysregulation, confer resistance to apoptosis, and promote genomic instability, thereby contributing to tumorigenesis and immune evasion.^2,3^ In regulatory regions of genes, particularly within promoters, DNA can adopt non-B-form secondary structures such as G-quadruplexes (G4s). These dynamic structures function as molecular switches that directly regulate gene transcription and influence cell fate.^4,5^ Stabilization of G4s can act as a transcriptional off-switch, inducing replication stress and DNA damage that ultimately trigger cell-cycle arrest and apoptosis.^4,6,7^ Consistent with this mechanism, increased G4 stability has been associated with suppression of multiple oncogenes, including *VEGFR*, *MYC*, *Bcl*-2, as well as reduced transcriptional activity across various cancer subtypes, including breast cancer.^8^ Complementary to traditional stabilization strategies, the destruction of G4 structural integrity through localized oxidative cleavage offers an irreversible approach to silencing oncogene expression. Therefore, strategies that target DNA secondary structures, impair DNA replication and transcription, and inhibit DNA repair pathways represent promising avenues for anticancer therapy.

Despite the widespread use of small molecules for DNA-targeted interventions and gene regulation, their actions often lack precise spatiotemporal control and cellular specificity, making it difficult to achieve noninvasive and highly selective modulation.^9^ Classical Pt-based chemotherapeutics such as cisplatin and carboplatin primarily induce DNA damage (for example, through DNA adduct formation), disrupt replication and transcription, and trigger tumor cell death.^10–12^ Oxaliplatin shares these features and additionally kills cancer cells by inducing ribosomal biogenesis stress.^13^ Effective application is challenged by the need for drugs to cross plasma and nuclear membranes, remain stable intracellularly, and avoid off-target interactions.^14^ For Pt-based drugs in particular, these factors collectively reduce controllability, yield unpredictable therapeutic outcomes, and cause frequent systemic side effects.^15^

Light-controlled biomolecular targeting addresses these limitations by providing external, on-demand spatiotemporal control.^4,16–18^ Photoactivatable small molecules combine photoregulation with gene regulation to enable precise control of DNA function, enhance anticancer efficacy, and reduce side effects.^19^ Within this context, photoactivatable Pt­(IV) complexes serve as promising photopharmaceutical platforms: upon irradiation, controlled photoreduction releases active Pt­(II) species and oxidizes biomolecules, enhancing therapeutic specificity and reducing systemic toxicity.^20–24^ A key challenge in translating this localized activation potential into superior anticancer efficacy, however, is ensuring that the photoactivated species act efficiently on defined molecular targets.

Herein, we report DNATplatin (Scheme 1a), a photooxidative Pt­(IV) prodrug designed to recognize and accumulate in cancer-cell DNA with specific high affinity for G4 DNA, thereby inhibiting oncogene transcription and eliciting antitumor immune responses (Scheme 1b). Beyond favorable aqueous solubility and high stability, DNATplatin enables *in situ*, light-triggered reduction and cleavage to release oxaliplatin; crucially, it simultaneously functions as a potent photooxidant that oxidatively destroys G4 DNA. This light-dependent destruction precisely suppresses the transcription of G4-associated oncogenes and concurrently activates antitumor immunity. In a mouse model of triple-negative breast cancer, the combined effects of genetic regulation and immune activation produced significant tumor growth suppression. Overall, this study demonstrates a sequential “target-and-destroy” strategy that couples Pt­(IV) photoreduction with target-specific photooxidation, enabling direct destruction of G4 DNA for precise genetic intervention in cancer.

**Scheme 1:**
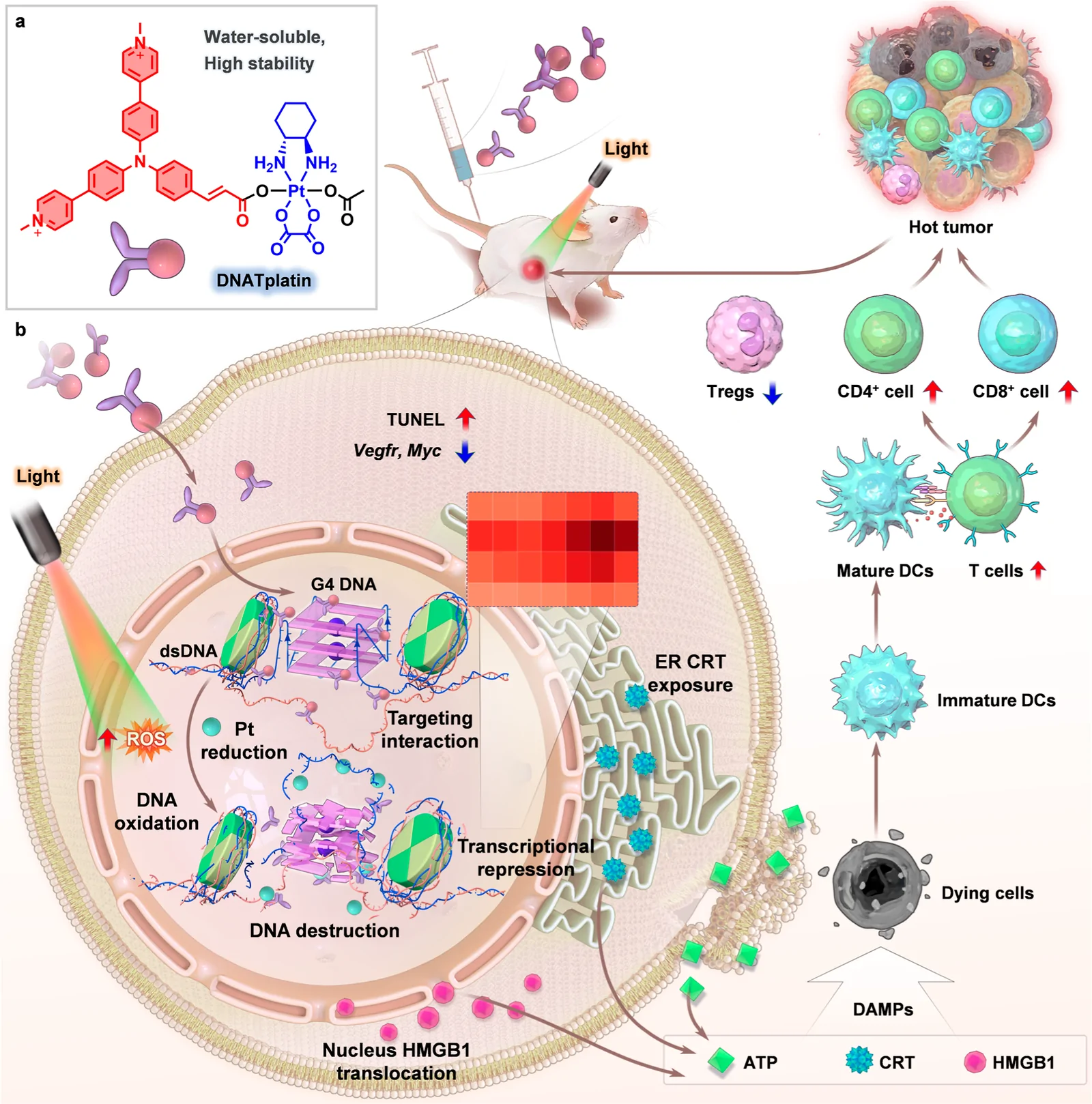
Schematic Illustration of a G4 DNA-Interacting Photooxidative Pt­(IV) Prodrug, Enabling *in Situ* Reduction to the Active Product, Oxaliplatin, While Simultaneously Oxidizing G4 DNA for Transcription Inhibition and Enhanced Immunotherapy^a^

a (a) Chemical structure of DNATplatin. (b) Following intravenous injection into tumor-bearing mice, DNATplatin selectively localizes within cancer cell DNA, particularly interacting with G4 DNA. Upon light irradiation, DNATplatin undergoes *in situ* reduction to the active product, oxaliplatin, while simultaneously oxidizing G4 DNA. This precisely suppresses the transcription of G4-associated oncogenes while simultaneously inducing an amplified immunotherapeutic effect. The combined effects of genetic regulation and immune activation lead to significant suppression of breast tumor growth in a mouse model.

## Results and Discussion

### Design and Characterization of Photoactive DNATplatin

Our study commenced with the design and synthesis of a photooxidative Pt­(IV) prodrug derived from oxaliplatin. To mitigate off-target effects and prevent uncontrolled activation, we selected a bis-*N*-methylpyridinium-modified triphenylamine ligand designed to provide both photosensitizing and DNA-interacting capabilities. We then functionalized the axial sites of the Pt­(IV) core with this ligand via Pt–O coordination. The synthesis (Scheme S1a,b) yielded the ligand (**4**) and the targeted prodrug (complex **7**, DNATplatin). Both compounds, along with key intermediates (**1**–**3**, **6**), were thoroughly characterized to confirm their structures (Figures S1–S9). The fully functionalized DNATplatin retains a high-water solubility of 18.82 mg Pt mL^–1^, which is 8.87-fold and 16.50-fold higher than oxaliplatin and dihydroxylated tetravalent oxaliplatin [HO-oxaPt­(IV)–OH], respectively (Table S1). These attributes effectively address the solubility limitations associated with certain Pt­(IV) prodrugs, thereby providing a strong foundation for the application of DNATplatin *in vivo*. Subsequent spectral analysis revealed a maximum absorption at 435 nm and a tail extending over to 530 nm (Figure 1a; blue line). Additionally, DNATplatin exhibited enhanced luminescence at 600 nm in both THF and glycerol (Figures S10 and 1a). These low-polarity and high-viscosity solvents serve as biomimetic environments, where glycerol simulates the motion-restricting rigid nuclear matrix and THF models the local hydrophobic pockets of biomolecules. Given the growing importance of two-photon excitation in biomedical imaging,^25–27^ we evaluated DNATplatin under two-photon irradiation at 880–930 nm and observed clear two-photon excited luminescence (Figure S11). The photosensitizing activity of DNATplatin was further assessed. Upon irradiation, DNATplatin induced a pronounced decrease in the absorption of 1,3-diphenylisobenzofuran (DPBF), indicating efficient generation of singlet oxygen (Figures 1b and S12). Together, these results establish the fundamental spectroscopic properties and preliminary photoreactivity of DNATplatin, providing a basis for subsequent mechanistic investigation of its light-induced chemical transformations.

**Figure 1:**
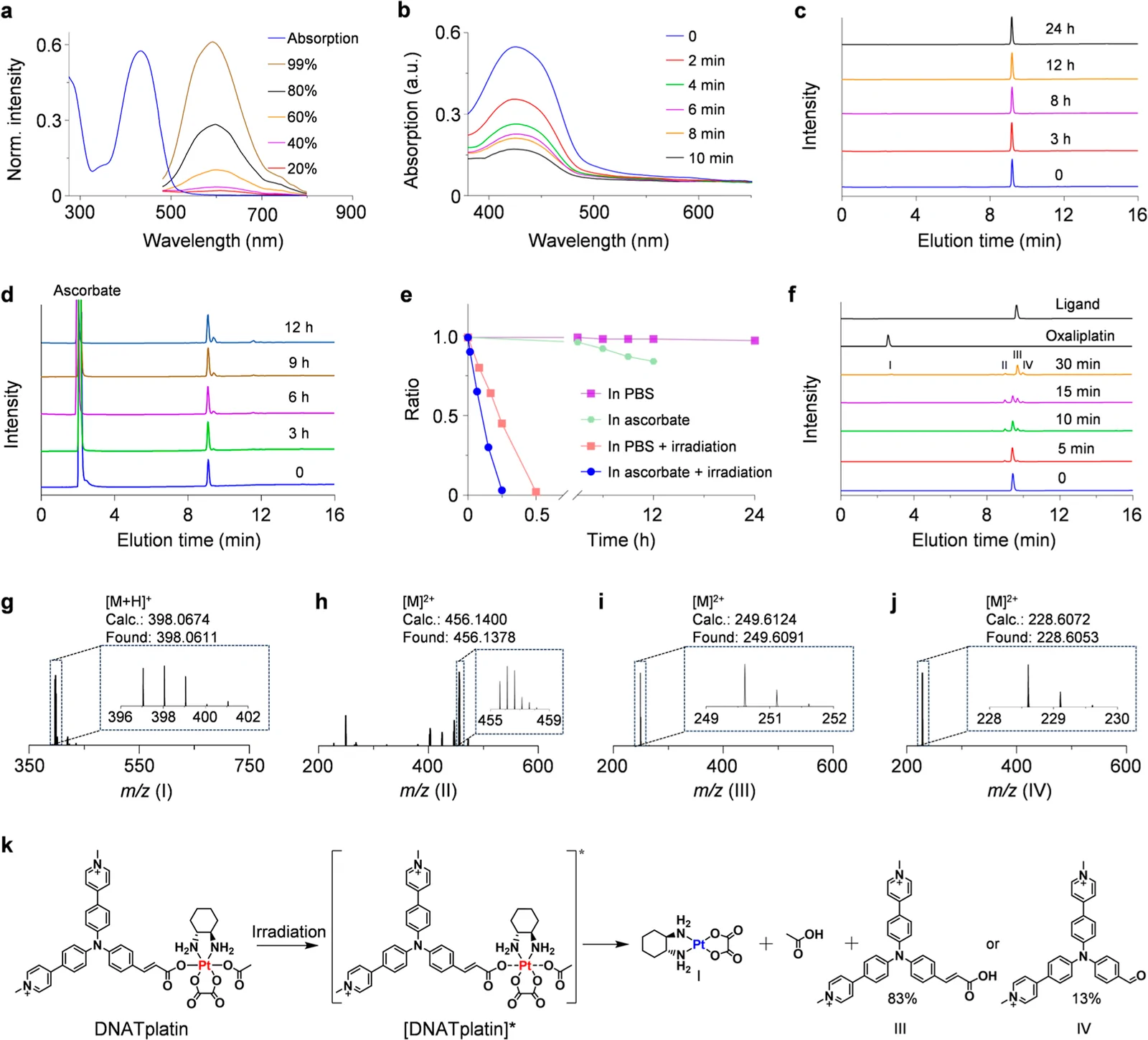
Photophysical and photochemical properties of DNATplatin. (a) Normalized absorption spectrum (blue line) of DNATplatin (5 μM) and its emission spectra in glycerol–water mixtures with glycerol volume fractions of 20%, 40%, 60%, 80%, and 99%. (b) Absorption spectra of a mixture of DPBF (30 μM) and DNATplatin (5 μM) under light irradiation, recorded every 2 min (λ_ex_ = 495 nm, 1 mW cm^–2^). (c,d) HPLC chromatograms of DNATplatin in PBS buffer (pH 7.4) alone or containing 2 mM sodium ascorbate in the dark. (e) Reduction ratio curves of DNATplatin (50 μM) in PBS buffer (pH 7.4), with or without 2 mM sodium ascorbate in the dark or under light irradiation. (f) HPLC chromatograms of DNATplatin (50 μM) in PBS buffer (pH 7.4) upon light irradiation for different durations (495 nm, 1 mW cm^–2^), newly generated peaks are labeled as I, II, III, and IV. (g–j) LC–MS analysis of the mass spectra corresponding to peaks I, II, III, and IV in (f). (k) Proposed reduction pathway for DNATplatin, inferred from the LC–MS products, along with the chemical structures of its resulting products.

The chemical stability of inert Pt­(IV) prodrugs before reaching the target site is crucial for achieving enhanced efficacy and reduced toxicity *in vivo*.^28,29^ Accordingly, we first evaluated the stability of DNATplatin under physiologically relevant conditions *in vitro* using high-performance liquid chromatography (HPLC). DNATplatin remained highly stable in PBS, showing no significant decomposition over 24 h (Figure 1c). In a reducing environment containing 2 mM sodium ascorbate, it retained 85% integrity after 12 h (Figure 1d,e). These findings indicate that DNATplatin can effectively maintain its inert Pt­(IV) state before reaching the desired target, establishing a basis for controlled, on-demand activation. Subsequently, we investigated the photoactivation behavior of DNATplatin in both PBS and ascorbate-containing buffer. Under light irradiation, DNATplatin was rapidly reduced in ascorbate-containing buffer, with the reaction completing within approximately 15 min (Figure S13). In the absence of ascorbate, the photoactivation proceeded more slowly but still occurred (Figure 1e,f), indicating that a reducing microenvironment facilitates photoactivation. These data demonstrate that DNATplatin undergoes a photolytic reduction process to release active Pt­(II) moieties. To elucidate the photoproducts, we analyzed irradiated samples by LC–MS. Before irradiation, a characteristic peak of DNATplatin appeared at 9.4 min (Figure 1f). After irradiation for 30 min, this peak gradually decreased and eventually disappeared, while four new peaks emerged at 2.8 min (I), 9.0 min (II), 9.8 min (III), and 10.0 min (IV). MS assignments identified peak I as oxaliplatin (Figure 1g), peak II as a deacetylated derivative of DNATplatin (Figure 1h and Scheme S1c), peak III as the axial ligand (Figure 1i; 83% by integral), and peak IV as an oxidized aldehyde byproduct (Figure 1j; 13%). Moreover, photooxidation in ascorbate-containing buffer did not lead to any detectable oxidized aldehyde byproducts from DNATplatin (Figure S13). Notably, the ligand alone, under the same irradiation conditions, remained largely intact, with only a minimal oxidation fraction (Figure S14). This result indicates that the presence of Pt­(IV) center enhances the photooxidative reactivity of DNATplatin relative to the free ligand. Furthermore, density functional theory (DFT) calculations showed that, in the presence of ascorbate, photoexcitation promotes DNATplatin from the ground state (set to 0 kcal mol^–1^) to an excited state at 56.0 kcal mol^–1^ (Figure S15). This reaction then proceeds downhill to generate oxaliplatin, free ligands, and dehydroascorbic acid, with an overall energy of −10.0 kcal mol^–1^. Consequently, these results reveal that DNATplatin can rapidly dissociate under light irradiation to exert a distinct photooxidation effect. Together, these results support a mechanism in which light triggers Pt–O bond cleavage in DNATplatin, thereby releasing active oxaliplatin and the axial ligands. Simultaneously, this process is accompanied by oxidation reactions driven by electron competition: 13% of the ligand undergo singlet oxygen-mediated oxidation to form an aldehyde (Figure 1k) or abstract electrons from surrounding molecules that easily lose electrons, such as ascorbic acid (Figure S13). In short, DNATplatin exhibits high stability under both physiological and reducing conditions, which supports its structural integrity before it reaches the target, and the complex undergoes efficient photoinduced activation to enable site-specific release of the active drug, also having the potential to function as a photooxidant for biomolecules–features that collectively underscore its potential for targeted phototherapy.

### Interacting and Photooxidation of G4 DNA by DNATplatin

To investigate the binding properties of DNATplatin to DNA, we first examined its interaction with short, defined-sequence dsDNA and G4 DNA. We measured the amount of Pt bound per nucleotide in the reaction and compared it to the formal ratio of Pt to nucleotide for both oxaliplatin and DNATplatin. The results indicated that DNATplatin exhibited a significantly higher binding affinity for G4 DNA compared to dsDNA, whereas oxaliplatin demonstrated a lower binding affinity than DNATplatin in both cases (Figure 2a,b). In addition, to visually confirm the binding of DNATplatin to G4 DNA, we utilized the characteristic of G4 DNA being unable to pass through a molecular weight cutoff ultrafiltration membrane. We conducted a comparative analysis of the UV–vis absorption spectra before and after ultrafiltration to observe the changes in the filtrate. Before ultrafiltration, the mixture of DNATplatin with G4 DNA exhibited a pronounced absorption band between 350 and 500 nm (Figure 2c). After ultrafiltration, this absorption band was substantially diminished in the filtrates, indicating retention of DNATplatin with DNA. Similar decreases were observed for mixtures of the ligand with G4 DNA (Figure S16a). In contrast, free DNATplatin (Figure 2d) or ligand (Figure S16b) without DNA lost only 20% of its absorption after ultrafiltration, confirming that the larger decreases in the mixtures of G4 DNA and complexes were DNA-dependent.

**Figure 2:**
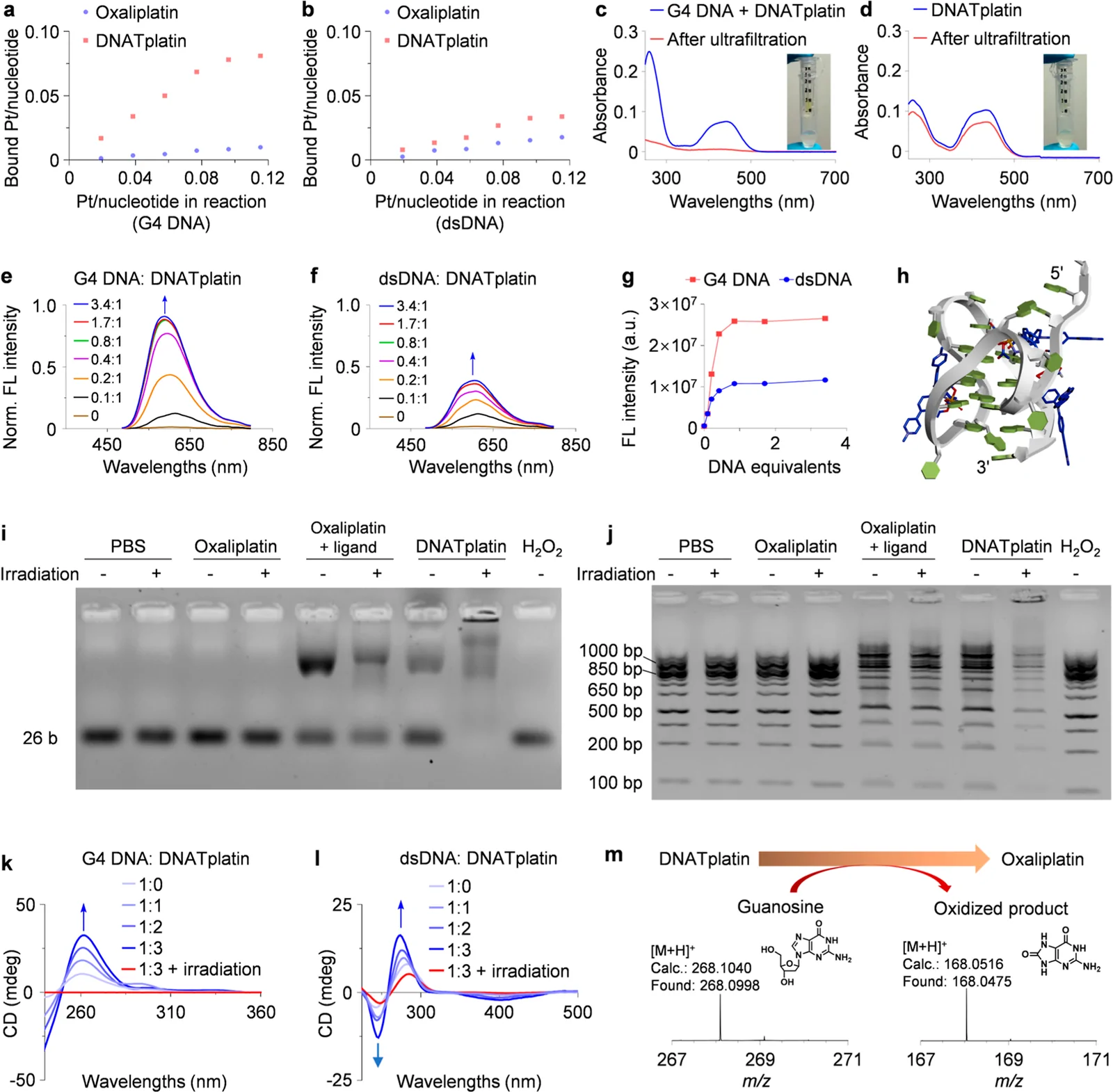
DNATplatin enables sequential and efficient interaction with and photooxidation of G4 DNA *in vitro*. Binding of DNATplatin to both Tel26-G4 DNA (a) (5′-AAAGGGTTAGGGTTAGGGTTAGGGAA-3′) and dsDNA (b) (5′-CAATCGGATCGAATTCGATCCGATTG-3′). *X*-Axis is the ratio of Pt to nucleotides in the reaction, and *y*-axis is the ratio of bound Pt per nucleotide. (c) Changes in the absorption spectra of G4 DNA upon mixing with DNATplatin before and after ultrafiltration. (d) Changes in the absorption spectra of the DNATplatin in PBS before and after ultrafiltration. Emission spectra of DNATplatin (2.0 μM) upon titration with increasing equivalents of G4 DNA (e) or dsDNA (f) in 10 mM Tris–HCl buffer containing 100 mM LiCl (pH 7.4, λ_ex_ = 465 nm). (g) The corresponding fluorescence enhancement curves. (h) Representative views of the docking model of G4 DNA binding with DNATplatin at a 1:3 molar ratio (PDB code: 6KFJ). Agarose gel electrophoresis analysis of G4 DNA (i) and a DNA ladder (j) mixed with Pt-containing complexes with or without light irradiation. CD spectra of 10 μM G4 DNA (k) and dsDNA (l) collected upon titration with increasing equivalents (from 1 to 3) of DNATplatin and following light irradiation at the third equivalent in 10 mM Tris–HCl buffer containing 100 mM LiCl, pH 7.4. (m) MS results for guanosine and its products oxidized by photoactivated DNATplatin. Irradiation conditions for photooxidation: λ_ex_ = 495 nm, 1 mW cm^–2^.

We next evaluated the binding behavior of DNATplatin toward G4 DNA and dsDNA by monitoring fluorescence responses upon interaction with diverse nucleic acid sequences under physiological conditions. DNATplatin induced significant fluorescence enhancement with all tested G4 DNA (Figures 2e and S17) and dsDNA (Figures 2f,g and S17) sequences, indicating preferential optical responsiveness to DNA structures. Furthermore, fluorescence titration assays indicated that DNATplatin exhibits efficient and selective interaction toward G4 DNA via a 1:3 cooperative binding mechanism, with an apparent dissociation constant (*K*_D_) of 21.33 ± 0.31 nM (Table S2). In comparison, its binding toward dsDNA follows a 1:1 saturation model with a *K*_D_ of 20.24 ± 3.68 nM. Notably, DNATplatin displays high selectivity for the G4 topology, similar to that of the ligand (Figure S18 and Table S2), showing a 58-fold fluorescence enhancement (*I*/*I*_0_) upon G4 DNA binding, which contrasts sharply with the weaker response (23-fold) observed with dsDNA.

To gain deeper insight into the binding modes at the atomic level, we performed molecular docking studies of DNATplatin with both types of DNA. These simulations revealed three predominant G4 binding conformations primarily located on three distinct domains of the G4 structure, stabilized by electrostatic interactions, hydrogen bonding, and π–π stacking (Figure S19).^30,31^ Notably, the conformation featuring DNATplatin oriented parallel to the G-quartet plane exhibited the lowest binding energy and the most robust π–π stacking interactions, collectively pointing to its optimal theoretical binding stability (Figure S19a). In contrast, docking with dsDNA reveals two predominant minor groove-binding modes for DNATplatin (Figure S20). In the first mode, the positively charged methylpyridinium group and Pt center face inward toward the minor groove interior, an arrangement stabilized by a combination of electrostatic interaction and hydrogen bonding (Figure S20a). In the second mode, both methylpyridinium groups point inward, with one contributing electrostatic stabilization and the other participating in π–π stacking with dsDNA (Figure S20b). Structural and simulation analyses further indicate that, compared with dsDNA, G4 DNA provides substantially greater binding space and more accessible binding sites for DNATplatin (Figures 2h and S21).^32^ Taken together, the fluorescence enhancements and docking results consistently support the higher recognition specificity of DNATplatin for G4 DNA over dsDNA.

The photoreduction of a Pt­(IV) prodrug to its active Pt­(II) species under light irradiation is fundamentally an electron-transfer process, in which surrounding molecules transfer electrons to the Pt center and are consequently oxidized. On this basis, we investigated whether DNATplatin, after its targeted binding to DNA, could trigger photoinduced redox activity and thereby cause potential oxidative damage to DNA structures. We first assessed the effects of photoactivated DNATplatin on different DNA structures using agarose gel electrophoresis.^33^ In the absence of irradiation, the binding of either the ligand or DNATplatin to G4 DNA or the dsDNA ladder produced slower-migrating bands (Figures 2i,j). Under light irradiation, control groups treated with a mixture of oxaliplatin and ligand exhibited merely moderate band attenuation across all DNA variants (Figures 2i,j). In stark contrast, the band corresponding to G4 DNA vanished completely upon irradiation in the DNATplatin-treated cohort (Figure 2i). Although the photoinduced effects on alternative dsDNA structures were less pronounced than those observed for G4 DNA, a discernible weakening of their respective bands remained evident (Figures 2j and S22a). This pronounced selectivity toward G4 DNA likely arises from its electron-rich G-tetrads and unique planar grooves, which favor DNATplatin binding and promote highly localized photooxidation, thereby making oxidative cleavage more efficient than in duplex DNA. Moreover, increasing the molar ratio of DNATplatin to DNA nucleotides progressively intensified photooxidative degradation of the dsDNA ladder bands, ultimately leading to complete band loss (Figure S22b). Taken together, these results demonstrate that DNATplatin not only binds DNA effectively but also exhibits strong photooxidative activity that is not achieved to the same extent with the mixture of oxaliplatin and ligand.

The unique asymmetric 3D structure of DNA gives rise to characteristic circular dichroism (CD) signals. Based on this property, we further employed CD spectroscopy to monitor spectral changes during the titration of dsDNA and G4 DNA with either the free ligand or DNATplatin, aiming to elucidate the DNATplatin’s capacity to modulate DNA secondary structures and assess its associated photooxidation effects. In the absence of the ligand or DNATplatin, G4 DNA exhibited a positive band near 260 nm (Figure 2k), whereas dsDNA displayed a positive CD band at 270 nm and a negative band at 245 nm (Figure 2l), consistent with their characteristic topologies.^4,34^ Without irradiation, increasing the molar ratio of either the ligand or DNATplatin to G4 DNA or dsDNA resulted in markedly enhanced characteristic CD ellipticity signals for both DNA types (Figures 2k,l and S23; blue curve), indicating selective conformational recognition and robust accommodation of their respective secondary structures upon binding.^5,34^ After light irradiation, however, the two treatments produced clearly different behaviors. In contrast to the moderate effect of the free ligand (Figure S23, red curves), DNATplatin completely flattened the ellipticity signal of G4 DNA (Figure 2k; red line) and significantly reduced dsDNA ellipticity (Figure 2l; red curves). Together, these results further demonstrate that DNATplatin exerts photooxidative damage on DNA to an extent not achieved by the ligand alone.

Although gel electrophoresis and CD analyses showed that photoactivated DNATplatin induces significant DNA damage, the underlying molecular mechanism remained unclear. To investigate this mechanism, we used guanosine, a key structural unit of G4 DNA, as a model substrate to examine its reaction with DNATplatin under light irradiation (Figure S24a). By employing LC–MS, we systematically compared the composition of the reaction mixture before and after irradiation. Upon photoactivation, the chromatographic peak corresponding to DNATplatin at position h completely disappeared, accompanied by the emergence of the ligand peak at position f (Figure S24b). Additionally, multiple new chromatographic peaks appeared at positions a, b, c, and e, which were identified by MS spectrometry as oxaliplatin, 8-OHG, guanine, and 8-oxo-dG, respectively (Figures S24b,c and 2m). In contrast, a control mixture containing only the ligand and guanine showed no significant new peaks or oxidation products under the same conditions (Figure S24d). These results demonstrate that photoactivation of DNATplatin induces both oxidative cleavage of nucleotide glycosidic bonds and oxidation of guanine at the C8 position. In comparison to the ligand, the Pt center in DNATplatin therefore serves as a critical driver of DNA photooxidation. Collectively, these findings show that DNATplatin not only anchors efficiently to DNA and stabilizes dsDNA and G4 DNA in the dark, but also, upon light irradiation, uniquely exhibits strong photooxidative activity that disrupts DNA structure. This effect is not reproduced by the ligand alone nor by oxaliplatin, highlighting the precise and light-controlled DNA damage enabled by DNATplatin.

### DNATplatin Accumulates in the DNA of Cancer Cells and Downregulates Oncogene Expression Upon Photoactivation

Positively charged therapeutics, such as cationic lipids, polyethylenimine, and cell-penetrating peptides, can bind negatively charged cell-membrane components (e.g., phospholipids and proteoglycans) through electrostatic interactions, thereby facilitating cellular capture and uptake.^35–37^ To determine whether DNATplatin is internalized by cancer cells and selectively accumulates in DNA, we assessed accumulation in 4T1 breast cancer cells using flow cytometry, CLSM imaging, and intracellular Pt quantification. Flow cytometry revealed a time-dependent increase in intracellular fluorescence, which reached a plateau at 8 h, showing that cellular uptake gradually slowed and fluorescence intensity stabilized over time (Figure 3a). CLSM analysis corroborated this trend and further revealed progressive nuclear localization of DNATplatin fluorescence (Figure 3b). In contrast, 4T1 cells treated with compound **2** (Scheme S1), which lacks the methylpyridinium moiety, displayed predominantly cytoplasmic fluorescence (Figure S25). Comparable uptake kinetics and nuclear localization were also observed in both A549 cells and Pt-resistant A549cisR cells, indicating that DNATplatin maintains efficient cellular accumulation even in drug-resistant cells (Figure S26). ICP–MS analysis revealed that after treatment of 4T1 cells with 10 μM DNATplatin for 12 h, the intracellular Pt content reached 125 ng per million cells, which was 2.8-fold higher than that observed in the oxaliplatin-treated group (Figure 3c). Moreover, DNATplatin exhibited two-photon excitation properties, and its fluorescence signal was localized almost entirely within the nucleus, further supporting its high affinity for DNA (Figure 3d). To validate the targeted accumulation of DNATplatin in DNA, we next measured Pt levels in the genomic DNA of cancer cells treated with different concentrations of Pt complexes. The Pt level in the genomic DNA of 4T1 cells treated with 2 μM DNATplatin reached 15 ng per million cells, which was 10 times higher than that measured in oxaliplatin-treated cells (Figure 3e). By comparing total cellular Pt accumulation with genomic DNA-associated Pt levels, we calculated a DNA-targeting efficiency of 63% for DNATplatin, significantly higher than the 20% observed for oxaliplatin (Figure 3f). Thus, DNATplatin exhibits efficient cellular accumulation, preferential nuclear targeting, and robust DNA enrichment, while also enabling two-photon nuclear imaging. These combined properties underscore its promise as a theranostic agent for integrated cancer imaging and targeted therapy.

**Figure 3:**
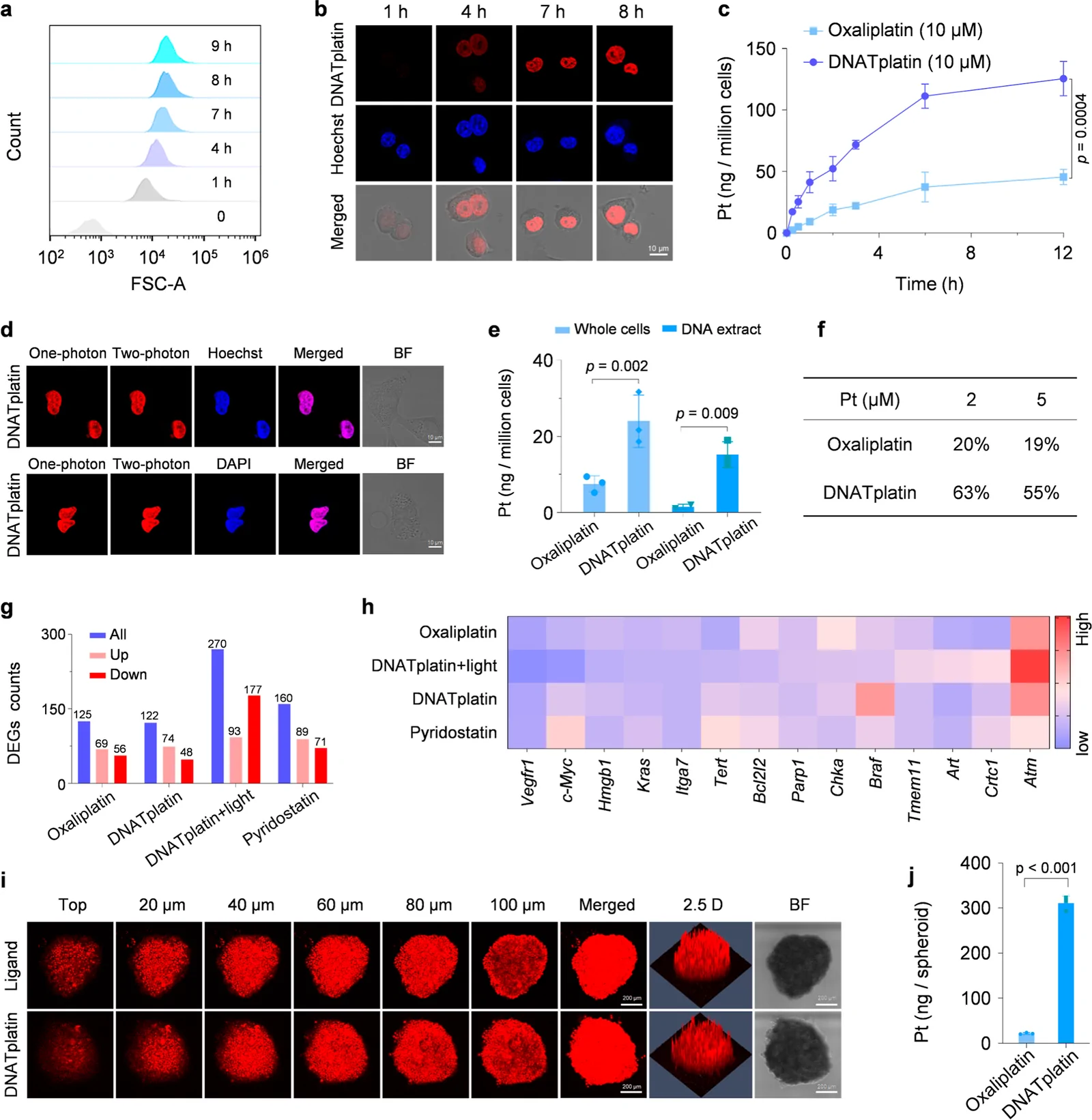
Cationic DNATplatin facilitates cellular uptake and genomic DNA accumulation, subsequently downregulating oncogene expression upon photoactivation. (a) Flow cytometry and (b) confocal images showing the accumulation of 10 μM DNATplatin in 4T1 cells at the indicated incubation time points. (c) Time-dependent cellular uptake of Pt in 4T1 cells after treatment with 10 μM DNATplatin (*n* = 3). (d) Confocal images showing the colocalization of Hoechst or DAPI with DNATplatin in A549 cells (one-photon λ_ex_ = 488 nm, two-photon λ_ex_ = 900 nm, and λ_ex_ = 405 nm for nuclear dyes; blue channel λ_em_ = 460–470 nm and red channel λ_em_ = 630–700 nm). (e) Pt levels in whole cells and the DNA extracts of 4T1 cells treated with 2 μM DNATplatin. (f) Pt targeting efficiency toward the genomic DNA of 4T1 cells treated with 2 or 5 μM DNATplatin. (g) Number of DEGs in 4T1 cells treated with 2 μM DNATplatin, with or without light irradiation for 30 min, as well as with 2 μM oxaliplatin, or pyridostatin. Irradiation conditions: λ_ex_ = 495 nm, 1 mW cm^–2^. (h) Heatmap analysis illustrating the expression levels of selected genes of interest. (i) Penetration ability of DNATplatin and ligand in 4T1 tumor spheroids. (j) Pt levels in 4T1 tumor spheroids (*n* = 3).

To elucidate the targeted regulatory effects on gene expression, particularly in genes harboring G4 motifs, we conducted RNA-seq transcriptome analysis in cancer cells under various treatment conditions. RNA-seq provides comprehensive quantification of cellular transcripts through high-throughput sequencing and is therefore well suited to sensitively detect gene expression and associated functional responses.^38,39^ Compared with PBS, oxaliplatin, DNATplatin, and the G4-stabilizer pyridostatin (PDS) each elicited distinct modifications in gene expression.^40,41^ Among these groups, the most substantial transcriptional changes were observed after treatment with DNATplatin under light irradiation (DNATplatin + light). In this group, a total of 270 significantly differentially expressed genes (DEGs) were identified, comprising 93 upregulated and 177 downregulated transcripts (Figures 3g and S27). When the analysis was focused specifically on the genes of interest, photoactivated DNATplatin was found to markedly suppress several key oncogenes, including *Vegfr-1* and *c-Myc* (Figure 3h).^42–44^ Consistent with our RNA-seq data, DNATplatin + light treatment notably reduced both the mRNA and protein expression levels of *Vegfr-1* and *c-Myc* (Figures S28). In contrast, the mixture of oxaliplatin and free ligand (with or without light) induced no significant alterations. Together, these findings support a model in which DNATplatin anchors to the G4-rich region in the dark and, upon photoactivation, primarily drives localized G4 structural destruction via photooxidation while concurrently releasing reduced active Pt­(II) moieties, thereby effectively inhibiting the expression of multiple cancer-related genes.

### Evaluating the Penetration Capability of DNATplatin in 3D Tumor Spheroids

3D tumor spheroids closely recapitulate key features of the *in vivo* tumor microenvironment and are widely used in oncology research, drug discovery, and regenerative medicine.^45^ CLSM revealed no significant decrease in fluorescence intensity up to a depth of 100 μm within tumor spheroids treated with either the free ligand or DNATplatin (Figure 3i), indicating uniform penetration. The accumulation of Pt in whole 3D spheroids following treatment with the DNATplatin was approximately 10-fold higher compared to oxaliplatin (Figure 3j). This finding demonstrates the ability of DNATplatin to efficiently penetrate the core region of 3D tumor spheroids. These results not only validate the efficacy of DNATplatin as a targeted drug delivery platform but also provide crucial preliminary justification for subsequent evaluation in animal models.

### Photoactivated DNATplatin Induces Cytotoxicity and Modulates Immune Responses in Cancer Cells

We next investigated the efficiency and mechanism of DNATplatin in killing cancer cells. Intracellular ROS levels in DNATplatin-treated 4T1 cells were measured using H_2_DCFDA.^46^ Relative to untreated cells and those kept in the dark and treated with DNATplatin, oxaliplatin, or the free ligand, a substantial increase in green fluorescence intensity was observed in cells treated with DNATplatin or ligand under irradiation, indicating elevated intracellular ROS levels (Figures 4a and S29). Notably, ROS signals induced by DNATplatin were concentrated in the nucleus, whereas the positive control menadione produced diffuse, cytoplasm-enriched fluorescence, supporting nuclear accumulation and light-triggered ROS generation by DNATplatin.

**Figure 4:**
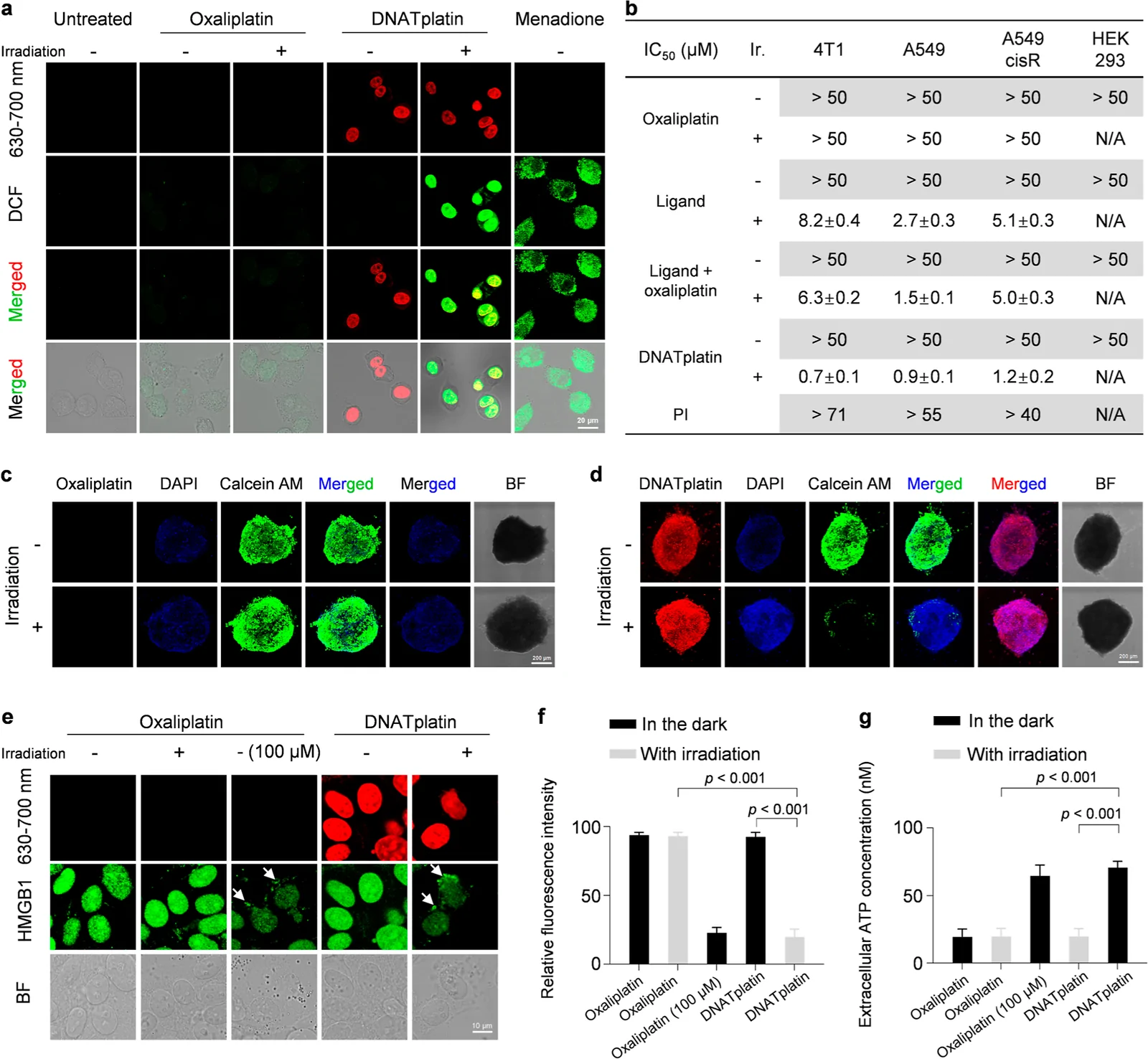
Photosensitive DNATplatin releases cytotoxic Pt­(II) and generates ROS at the DNA site to induce immunogenic cell death in cancer cells. (a) Confocal fluorescence images of H_2_DCFDA-stained 4T1 cells treated with oxaliplatin (10 μM), DNATplatin (10 μM), or menadione for 8 h, followed by green light irradiation (1 mW cm^–2^) for 2 min (λ_ex_ = 488 nm, λ_em_ = 517–527 nm). (b) IC_50_ values determined by the MTT assay at 72 h after irradiation (mean ± SD; *n* = 3). The selectivity index (PI) is defined as the ratio of the IC_50_ values of DNATplatin without and with light irradiation. (c,d) Live/dead cell staining of 4T1 tumor spheroids under different treatments. The spheroids were treated with 10 μM DNATplatin for 6 h, followed by replacement with fresh medium, irradiation with or without light for 30 min, and subsequent culturing for 24 h. Live/dead cells were stained with Calcein AM/DAPI, and spheroids were observed using confocal microscopy. (e) Confocal immunofluorescence images showing the translocation of HMGB1 from the nucleus to the cytoplasm in 4T1 cells treated with DNATplatin (2 μM), oxaliplatin (2 and 100 μM), followed by light irradiation for 30 min (λ_ex_ = 488 nm, green channel λ_em_ = 517–527 nm and red channel λ_em_ = 630–700 nm). (f) Semiquantitative analysis of HMGB1 immunofluorescence intensity. (g) ATP efflux in 4T1 cells treated with DNATplatin (2 μM), oxaliplatin (2 μM) followed by irradiation. Irradiation conditions for the cell experiments: λ_ex_ = 495 nm, 1 mW cm^–2^. *P*-Values were calculated using a two-tailed, unpaired Student’s *t*-test.

We then assessed the cytotoxicity of DNATplatin in comparison with oxaliplatin and the free ligand in 4T1, A549, and Pt-resistant A549cisR cells, with human embryonic kidney cells (HEK293) included for safety evaluation. In the dark, the IC_50_ values of oxaliplatin, the mixture of ligand and oxaliplatin, and DNATplatin were all greater than 50 μM in all four cell lines (Figure 4b), indicating low dark toxicity for these treatments. Under irradiation, oxaliplatin showed little change in cytotoxicity compared to the dark condition, suggesting that light had a minimal effect on its cytotoxicity. The mixture of ligand and oxaliplatin demonstrated IC_50_ values of 6.3, 1.5, and 5.0 μM in 4T1, A549, and A549cisR cells, respectively. In contrast, DNATplatin showed apparently stronger photocytotoxicity, with IC_50_ values as low as 0.7 μM in 4T1 cells, 0.9 μM in A549 cells, and 1.2 μM in A549cisR cells. Notably, under light exposure, the IC_50_ value of DNATplatin in 4T1 cells was only one-ninth that of the mixture of ligand and oxaliplatin, and its phototoxicity index (PI) was over 70. In 4T1 3D tumor spheroids, DNATplatin, but not oxaliplatin or the ligand, effectively induced photoactivated cell death (Figures 4c,d and S30). These results confirm that DNATplatin demonstrates excellent dark stability and low basal toxicity yet confers significantly stronger light-dependent anticancer activity, outperforming the mixture of ligand and oxaliplatin.

Anticancer therapies that induce genetic damage or inhibit cellular functions can dynamically modulate the immunogenicity of cancer cells during cytotoxic action, resulting in dual immunoregulatory outcomes, including either immunosuppression or immune enhancement.^47–49^ To probe these effects, we initially assessed the impact of the photoactivatable DNATplatin on immunogenicity at the cellular level.^50^ Immunofluorescence analysis demonstrated that 4T1 cells treated with photoactivated DNATplatin or a high concentration (100 μM) of oxaliplatin exhibited notable nuclear translocation of high-mobility group box 1 (HMGB1; Figures 4e and S31a).^51^ A significant reduction in nuclear fluorescence intensity was observed in these treatments compared to other conditions (Figure 4f). A similar pattern was observed for calreticulin (CRT), with the photoactivated DNATplatin and high-concentration oxaliplatin groups showing increased surface exposure (Figure S32).^52^ In contrast, only minimal externalization of CRT occurred following treatment with irradiated ligand or low concentrations of oxaliplatin. Consistently, ATP efflux assays confirmed that both photoactivated DNATplatin and high-concentration oxaliplatin induced a significant increase in ATP release, and the level from photoactivated DNATplatin was 3.7-fold higher than that induced by non-photoactivated DNATplatin (Figures 4g and S31b). Based on these findings, DNATplatin demonstrates low dark toxicity while exhibiting potent anticancer activity and modulating immune responses upon photoactivation, making it a promising candidate for targeted cancer therapy *in vivo*.

### *In Vivo* Tumor Inhibition and Activation of the Immune System by DNATplatin

Biosafety is a critical consideration for *in vivo* drug administration. To assess the biosafety of DNATplatin, we first evaluated its hemocompatibility using an *in vitro* hemolysis assay.^53^ Unlike the positive control Triton X-100, DNATplatin showed no significant hemolysis and performed comparably to the negative control oxaliplatin (Figure S33). Additionally, a single intravenous injection of DNATplatin (3.5 mg Pt kg^–1^ body weight) was administered to BALB/c mice; no significant decrease in body weight was observed over 10 days (Figure S34). These findings suggest that DNATplatin does not induce significant systemic toxicity and support the feasibility of *in vivo* administration.

Subsequently, we established a 4T1 tumor-bearing mouse model in BALB/c mice, which recapitulates the malignant progression and tumor microenvironment characteristics of triple-negative breast cancer. Following the timeline shown in Figure 5a, we conducted *in vivo* studies on the biodistribution, antitumor effects, and immune activation of DNATplatin. To investigate the biodistribution of DNATplatin and determine the optimal timing for postadministration irradiation, we monitored fluorescence signals at the tumor site after a single intravenous injection using an *in vivo* imaging system (Figure 5b). DNATplatin exhibited significant fluorescence accumulation at the tumor site, peaking at approximately 4.0 h postinjection (Figure 5c). The fluorescence intensity decreased markedly after 12 h, with only weak signals remaining at 24 h, indicating rapid clearance of the prodrug. To further investigate the tumor targeting of DNATplatin, we performed *ex vivo* imaging in mice. At 24 h after administration, tumor tissues exhibited the highest fluorescence intensity among all collected tissues, measuring 40-fold and 1.7-fold higher than that of the kidneys and liver, respectively (Figure 5d). When the tumors reached approximately 100 mm^3^ in size, the mice were treated intravenously with saline, oxaliplatin, a mixture of oxaliplatin and ligand (oxaliplatin + ligand), or DNATplatin on days 0, 2, and 4. After each injection, localized light irradiation was applied to both the oxaliplatin + ligand and DNATplatin groups (Figure S35). On day 10, tumor tissues were collected for immunophenotyping analysis (Figure S36). The results showed a significant increase in the proportion of mature dendritic cells (CD80^+^ and CD86^+^) in the photoactivated DNATplatin group, reaching 16%, which was 3.3 times higher than that in the oxaliplatin group (Figure 5e,f). Additionally, the infiltration rates of CD4^+^ and CD8^+^ T cells in the tumor tissue were dramatically elevated in this group (73%), significantly surpassing those in oxaliplatin (29%), the mixture of oxaliplatin and ligand (39%), and nonirradiated DNATplatin (53%) groups (Figure 5g,h). These findings suggest that photoactivated DNATplatin effectively promotes T cell proliferation and activation, thereby stimulating an antitumor immune response. Furthermore, the proportion of immunosuppressive regulatory T cells (Tregs, Foxp3^+^) among CD4^+^ T cells was significantly reduced in the photoactivated DNATplatin group (20%). In comparison, the Treg proportions in the saline, oxaliplatin, oxaliplatin + ligand, oxaliplatin + ligand + light, and nonirradiated DNATplatin groups were 40%, 33%, 28%, 23%, and 27%, respectively (Figure 5i,j). These results further confirm that photoactivated DNATplatin effectively modulates the tumor immune microenvironment, successfully eliciting a systemic adaptive immune response in 4T1 tumor-bearing mice.

**Figure 5:**
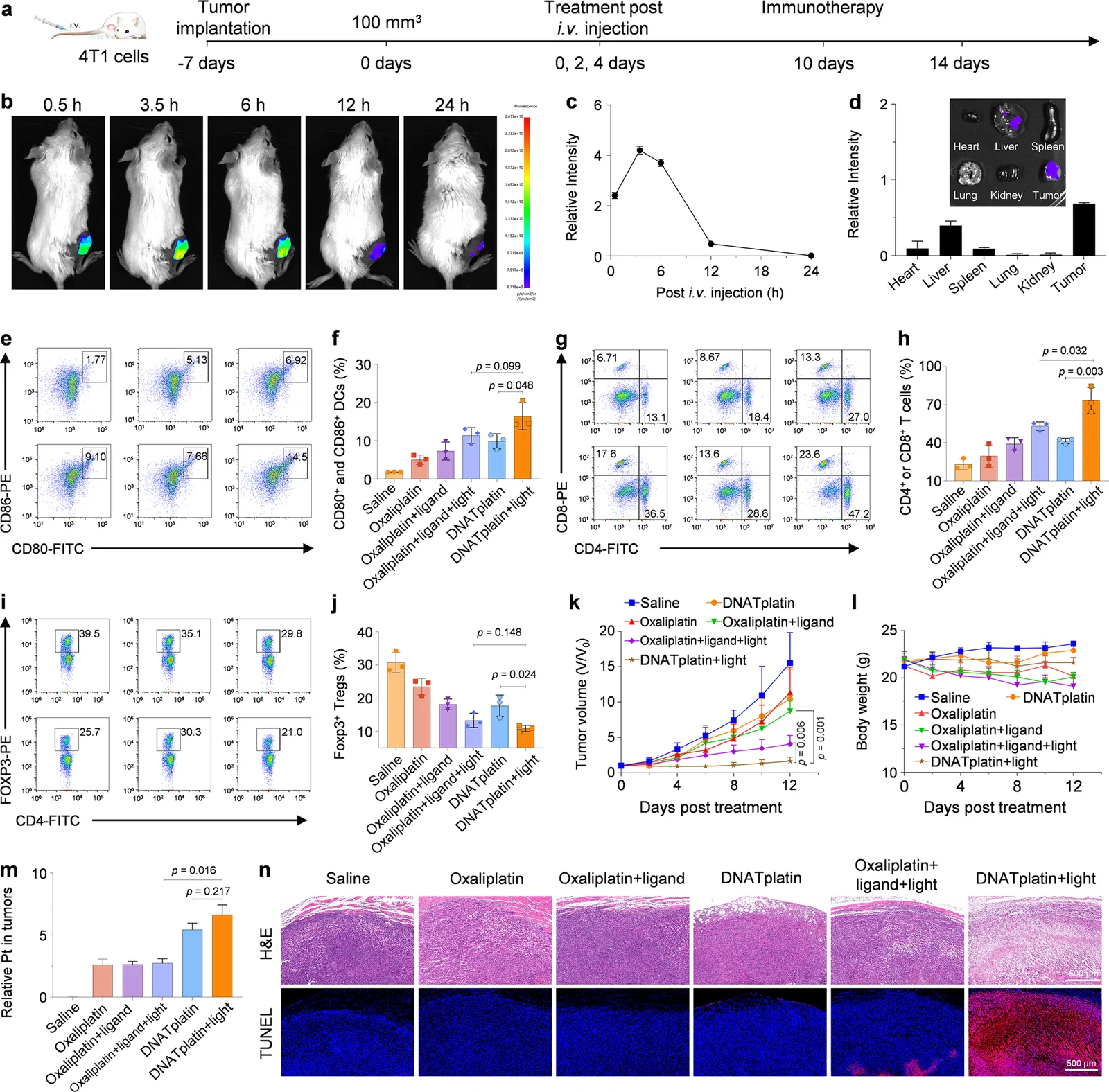
Antitumor and immunomodulatory activity of DNATplatin *in vivo*. (a) Timeline for tumor establishment, imaging, treatment, and immune cell detection. (b) *In vivo* fluorescence imaging of mice at different time points after a single tail vein injection of DNATplatin. (c) Fluorescence intensity statistical analysis of the tumor site. (d) *Ex vivo* fluorescence imaging and quantification of fluorescence intensity. Flow cytometry analysis and quantification of CD80 and CD86 positive antigen-presenting cells (e,f), CD4 or CD8 positive effector T cells (g,h), CD4 and FOXP3 positive Treg cells (i,j). The flow cytometry plots, shown from left to right and top to bottom, correspond to the following groups: saline, oxaliplatin, oxaliplatin + ligand, oxaliplatin + ligand + light, DNATplatin, and DNATplatin + light (*n* = 3). Relative tumor volume inhibition curves (k) and body weight changes (l) of 4T1 tumor-bearing mice (*n* = 5) after three treatments every 2 days at a dose of 3.5 mg Pt kg^–1^ body weight. (m) Pt levels in tumor tissue on day 12 after the mice were humanely sacrificed. (n) H&E and TUNEL staining of 4T1 tumor tissue sections from different treatment groups. Irradiation conditions for the animal experiments: λ_*ex*_ = 495 nm, 66 mW cm^–2^. *P*-values were calculated using a two-tailed, unpaired Student’s *t*-test.

Tumor suppression results showed that treatment with oxaliplatin alone or a mixture of oxaliplatin and ligand only mildly inhibited tumor growth (Figure 5k and S37) and led to body weight loss in mice (Figure 5l), whereas non-photoactivated DNATplatin also showed only mild tumor growth inhibition but without significant body weight loss. In contrast, the photoactivated DNATplatin group demonstrated a substantial antitumor effect, achieving a 90% inhibition of tumor volume without causing noticeable changes in body weight. The increased Pt accumulation in the tumor tissues of mice treated with DNATplatin and light activation further supports its antitumor efficacy (Figure 5m). Further H&E staining, TUNEL, and γ-H2AX staining assays (Figures 5n and S38) of tumor tissue sections indicated that photoactivated DNATplatin effectively induced DNA damage and cell death in tumor cells.

## Conclusions

DNA remains the central blueprint of life processes, and G4 DNA structures located in oncogene promoters represent a particularly compelling yet challenging target for precise cancer intervention. In this study, we developed DNATplatin, a pioneering Pt­(IV) complex rationally engineered to bridge the gap between high-precision DNA targeting and potent Pt-enhanced photooxidation. In addition to its robust two-photon absorption and emission properties, DNATplatin leverages bis-*N*-methylpyridinium moiety that synergistically enhances aqueous solubility and promotes the selective recognition of DNA secondary structures. Remarkably, DNATplatin exhibits a 10-fold increase in DNA binding relative to oxaliplatin, coupled with a 2.5-fold stronger emission turn-on that highlights its signaling selectivity for G4 DNA over duplex DNA, enabling superior target accumulation. Upon precise localization, DNATplatin leverages its exceptional photooxidation capability as the primary driver to photooxidatively dismantle G4 structures, abolish CD signals, and even propagate chemical stress to cleave glycosidic bonds, leading to base dissociation. This orchestrated attack triggers profound oxidative stress while silencing a spectrum of oncogenes, such as c-*Myc* and *Vegfr*, providing a controllable and highly effective therapeutic strategy to circumvent traditional Pt drug resistance.

Unlike conventional Pt-based agents and photosensitizers, our Pt­(IV) photooxidant exerts superior DNA-damaging potency that extends beyond the traditional reliance on Pt-DNA adduct formation, thereby effectively circumventing established resistance mechanisms. The therapeutic efficacy of this Pt­(IV) photooxidant was rigorously validated in a triple-negative breast cancer mouse model, where it not only suppressed tumor growth but also triggered a robust systemic antitumor immune response. This potent immunity is driven by a cooperative cascade where acute photooxidative stress, characterized by elevated intracellular ROS, serves as the primary trigger for DAMP emission to initiate ICD. Concurrently, the transcriptional silencing of *c-Myc* and *Vegfr* functionally amplifies these ICD signals by depleting Foxp3^+^ Tregs, thereby transforming the tumor microenvironment from cold to hot. Consequently, DNATplatin treatment promoted marked activation of CD4^+^ and CD8^+^ T cells while concurrently reducing the Foxp3^+^ regulatory T-cell population, showcasing a profound advantage over standard chemotherapy and classical photodynamic therapy. Collectively, this study pioneers a “target-and-destroy” oncological strategy that synergizes precision targeting with photooxidative amplification. Although the current activation wavelength limits deep tissue penetration, incorporating advanced fiber-optic technologies or further optimizing the ligand design to red-shift the absorption profile offers a promising route to maximize both treatment depth and topology-selective targeting precision. As proof of concept, our findings indicate that harnessing the synergy between targeting precision and metal-enhanced photooxidative activity may provide a promising route toward more selective and effective metal-based therapeutics.

## Supplementary Material



## Notes

Additional experimental details, materials, and methods, including photographs of the experimental setup, procedures for the synthesis of compounds, DFT calculation details, NMR spectra, and additional results (PDF)
