# Supplementary material for: G‑Quadruplex-Interacting Photooxidative Platinum(IV) Prodrug for Inhibiting Oncogene Transcription and Amplifying Antitumor Immunity
Source: J Am Chem Soc. 2026 Oct 21;148(43):47396–408. doi: 10.1021/jacs.6c16762 (PMC13741817; doi:10.1021/jacs.6c16762)
Supplement: Supplementary file 1 [file ja6c16762_si_001.pdf]

Supporting Information

G-Quadruplex-Interacting Photooxidative Platinum(IV) Prodrug for Inhibiting Oncogene Transcription and Amplifying Antitumor Immunity

Dengshuai Wei,[a, b] Yijia Chen,[a, c] Jiaqian Xu,[a, c] and Guangyu Zhu*[a, c]

[a] Department of Chemistry, City University of Hong Kong, Kowloon Tong, Hong Kong SAR, P. R. China

[b] Department of Pharmaceutics, School of Pharmacy, Qingdao University, Qingdao 266021, P. R. China

[c] City University of Hong Kong Shenzhen Research Institute, Shenzhen 518057, P. R. China

Table of Contents

Experimental Procedures……………………………………………………………………………2-16
Scheme S1. Synthetic route of target compounds and complexes………………………………………………17
Figure S1. ^{1}H NMR and ^{13}C NMR spectra of compound 2 in DMSO-d_6……………………………………18
Figure S2. Chemical structure and ESI-HRMS spectra of compound 2………………………………………19
Figure S3. ^{1}H NMR and ^{13}C NMR spectra of compound 3 in DMSO-d_6……………………………………20
Figure S4. Chemical structure and ESI-HRMS spectra of compound 3………………………………………21
Figure S5. ^{1}H NMR and ^{13}C NMR spectra of compound 4 in DMSO-d_6……………………………………22
Figure S6. Chemical structure and ESI-HRMS spectra of compound 4………………………………………23
Figure S7. ^{1}H NMR and ^{13}C NMR spectra of complex 7 in DMSO-d_6………………………………………24
Figure S8. ^{195}Pt NMR and HSQC spectra of complex 7 in DMSO-d_6…………………………………………25
Figure S9. Chemical structure (a), HPLC (b), and ESI-HRMS (c-d) spectra of complex 7…………………26
Figure S10. Normalized fluorescence emission spectra of DNATplatin in water and varying THF ratios……27
Figure S11. The normalized two-photon excited emission spectra of DNATplatin……………………………28
Figure S12. DPBF detection of ROS generation……………………………………………………………29
Figure S13. HPLC chromatograms of DNATplatin in a 2 mM sodium ascorbate PBS buffer………………30
Figure S14. The photoreaction equation for ligands and HPLC chromatograms of ligand………………31
Figure S15. Density functional theory calculation……………………………………………………………32
Figure S16. Changes in the absorption spectra of dsDNA or G4 DNA…………………………………………33
Figure S17. Fluorescence intensity curves of DNATplatin with increasing equivalents of dsDNA or G4 DNA titration…………………………………………………………………………………………………34
Figure S18. Fluorescence responses of the ligand upon titration with G4 DNA and dsDNA…………………35
Figure S19. Docking model of G4 DNA binding with DNATplatin at 1:1 ratio…………………………………36
Figure S20. Molecular docking between dsDNA and DNATplatin………………………………………………37
Figure S21. Docking model of G4 DNA binding with DNATplatin at 1:3 ratio…………………………………38
Figure S22. Images of dsDNA by agarose gel electrophoresis…………………………………………………39
Figure S23. CD spectra of dsDNA or G4 DNA upon titration with DNATplatin…………………………………40
Figure S24. DNATplatin oxidizes guanosine under light irradiation……………………………………………41
Figure S25. Confocal images for the uptake of 10 μM compound 2 by 4T1 cells……………………………42
Figure S26. Flow cytometry and confocal images for the uptake of DNATplatin by cells……………………43
Figure S27. Counts of differentially expressed genes (DEGs) in 4T1 cells………………………………………44
Figure S28. Relative mRNA expression levels determined by RT-qPCR………………………………………45
Figure S29. Fluorescent confocal images of H_2DCFDA-stained 4T1 cells……………………………………46
Figure S30. The live/dead cell staining of 4T1 tumor spheroids under different treatments………………47
Figure S31. Immunofluorescence confocal images of HMGB1…………………………………………………48

Figure S32. Immunofluorescence confocal images of CRT……………………………………………………..49
Figure S33. *In vitro* hemolysis assay of all Pt containing formulation……………………………………....50
Figure S34. Body weight changes in mice after a single intravenous injection of DNATplatin..…………….51
Figure S35. Physical picture of the laser……………………..……………………………………….….52
Figure S36. Gating strategy for flow cytometry of immune cells.……………………………………………53
Figure S37. Tumor weights of all mice were measured after three treatments on day 12...……………………54
Figure S38. *γ*-H2AX staining of 4T1 tumor tissue sections…….…………………………………………..…55
Table S1. Solubility of the complex 7 in water………………………………………………...…..……….56
Table S2. Comparative binding parameters of DNATplatin and free ligand toward DNA……………………57
References.…………………………………………………………………………………………..…..58

Experimental Procedures

Materials and Methods

All chemicals and solvents used in the experiment were sourced from commercial suppliers without further purification. The reactions were conducted under standard atmospheric conditions and were shielded from light. The cell culture media, including Dulbecco's modified Eagle medium (DMEM), Roswell Park Memorial Institute (RPMI) 1640, and phenol red-free DMEM/F-12, along with trypsin, phosphate-buffered saline (PBS, pH 7.4), and fetal bovine serum (FBS), were obtained from Life Technologies.

The light source consisted of an LED (1 mW cm^{-2}) or a laser (66 mW cm^{-2}), both equipped with a beam expander (purchased from Shenzhen Fuzhe Technology Co., Ltd., China), to yield a spot diameter of approximately 0.5 cm. The light power intensity was measured with a PM200 touchscreen power and energy meter console (Thorlabs). The LED light was used for *in vitro* testing, while the laser was employed for *in vivo* testing. NMR spectra were recorded using either a Bruker AVANCE III 400 MHz spectrometer or a Bruker Ascend AVANCE III 600 MHz spectrometer, both at room temperature. High-performance liquid chromatography (HPLC) analysis was performed using a Shimadzu Prominence System equipped with a C18 column (Phenomenex, Gemini, 5 μm, 110 Å, 4.6 mm × 250 mm). For complex purification, a Waters semi-preparative HPLC column (XBridge BEH C18 OBD Prep Column, 130 Å, 5 μm, 10 mm × 250 mm) was used. LC-MS was conducted on an HPLC coupled with a high-resolution mass spectrometer (Sciex X500R Q-TOF). Confocal images were captured with Leica SP5 or SPE laser scanning confocal microscopes. Absorption or fluorescence readings were recorded using a Molecular Devices SpectraMax ID5 Microplate Reader, while fluorescence spectra were analyzed using a spectrofluorometer system (Edinburgh FLS980). The MTT assay was conducted using the Molecular Devices SpectraMax ID5 Microplate Reader. The *in vivo* imaging was conducted using an *in vivo* imaging system (IVIS, PerkinElmer).

Reagents and antibodies

The following reagents and kits were used in this study: the GeneJET Genomic DNA Purification Kit (K0722) and the ATP Determination Kit (A22066) were obtained from Thermo Fisher Scientific. Anti-CD86 and anti-CD80 antibodies were purchased from Abcam. The terminal deoxynucleotidyl transferase dUTP nick end labeling (TUNEL) assay kit was sourced from Roche (Basel, Switzerland). Anti-CD80-PE, anti-CD86-APC, anti-CD3-APC, anti-CD4-FITC, and anti-CD8-PE antibodies were purchased from BioLegend, Inc. (San Diego, CA, USA). Additionally, anti-heme oxygenase 1 antibody (ab13248), rabbit polyclonal calreticulin antibody (ab2907), and rabbit monoclonal anti-HMGB1 antibody (ab79823), as well as goat anti-rabbit IgG H&L (FITC) secondary antibody (ab7086) and goat anti-rabbit IgG H&L (Alexa Fluor® 647) secondary antibody (ab150115), were purchased from Abcam, while the cellular reactive oxygen species (ROS) assay kit (H_2DCFDA) was sourced from

Thermo Fisher Scientific.

Synthesis and characterization

Synthesis of methyl (*E*)-3-(4-(bis(4-(pyridin-4-yl)phenyl)amino)phenyl)acrylate (**2**): To a stirred solution of 4-(bis(4-(pyridin-4-yl)phenyl)amino)benzaldehyde (**1**, 400 mg, 0.94 mmol), synthesized according to the literature method[1], in 20 mL of DMF, the Wittig reagent (methyl(triphenylphosphoranylidene)acetate, 375 mg, 1.12 mmol, 1.2 eq) was added. The reaction mixture was stirred at room temperature for 36 h. After completion, the reaction mixture was subjected to silica gel column chromatography for purification. The desired product (**2**) was obtained as a yellow solid (400 mg, 88% yield). ^{1}H NMR (600 MHz, DMSO-d_6) δ 8.65 – 8.60 (m, 4H), 7.86 – 7.80 (m, 4H), 7.74 – 7.67 (m, 6H), 7.66 – 7.59 (m, 2H), 7.60 – 7.50 (m, 1H), 7.26 – 7.19 (m, 4H), 7.10 – 7.06 (m, 2H), 6.53 (d, *J* = 16.0 Hz, 1H), 3.72 (s, 3H). ^{13}C NMR (151 MHz, DMSO-d_6) δ 167.32, 150.70, 148.82, 147.61, 146.61, 144.36, 132.72, 131.99, 131.92, 130.45, 129.28, 129.20, 128.70, 125.29, 123.67, 121.19, 116.51, 51.86. HRMS *m/z*: $[M+2H]^{2+}$ Calcd. for $C_{32}H_{27}N_3O_2^{2+}$, 242.6046; found 242.6014; $[M+H]^+$, Calcd. for $C_{32}H_{26}N_3O_2^{+}$, 484.2020, found, 484.1982.

Synthesis of (*E*)-4,4'-(((4-(3-methoxy-3-oxoprop-1-en-1-yl)phenyl)azanediyl)bis(4,1-phenylene))bis(1-methylpyridin-1-ium) (**3**): Compound **2** (400 mg, 0.83 mmol) was dissolved in 10 mL of DMF. To this solution, methyl iodide (MeI, 258 μL, 4.15 mmol, 5.0 eq) was added. The mixture was stirred at 80°C for 12 h. Then, the mixture was cooled and poured into 45 mL of diethyl ether. The desired precipitate (**3**) was then collected and dried to afford a red solid (458 mg, 73% yield). ^{1}H NMR (600 MHz, DMSO-d_6) δ 8.95 (d, *J* = 6.7 Hz, 4H), 8.47 – 8.44 (m, 4H), 8.13 – 8.10 (m, 4H), 7.80 (d, *J* = 8.5 Hz, 2H), 7.69 (d, *J* = 16.0 Hz, 1H), 7.32 – 7.28 (m, 4H), 7.21 (d, *J* = 8.6 Hz, 2H), 6.63 (d, *J* = 16.0 Hz, 1H), 4.31 (s, 6H), 3.74 (s, 3H). ^{13}C NMR (151 MHz, DMSO-d_6) δ 167.20, 153.54, 149.73, 147.63, 145.84, 144.06, 131.99, 131.22, 130.70, 130.28, 128.58, 125.94, 124.65, 123.60, 117.74, 51.98, 47.40. HRMS *m/z*: $[M]^{2+}$, Calcd. for $C_{34}H_{31}N_3O_2^{2+}$, 256.6203; found, 256.6177.

Synthesis of (*E*)-4,4'-(((4-(2-carboxyvinyl)phenyl)azanediyl)bis(4,1-phenylene))bis(1-methylpyridin-1-ium) (**4**): Compound **3** (200 mg) was dissolved in 1 mL of MeOH. To this solution, 1 mL of a 1 N aqueous lithium hydroxide (LiOH) solution was added. The mixture was stirred at room temperature for 4 h. Subsequently, the mixture was purified by preparative HPLC. For HPLC purification, a C18 column was used with a mobile phase gradient: A phase – 0.1% formic acid in H_2O, B phase – 0.1% formic acid in CH_3CN. The gradient was as follows: at 0.01 min, 95% A and 5% B; at 7.00 min, 51% A and 49% B; at 13.00 min, 45% A and 55% B; at 14.00 min, 95% A and 5% B; and at 18.00 min, 95% A and 5% B. Finally, the product was characterized by appropriate analytical techniques. Red solid, 80% yield. ^{1}H NMR (600 MHz, D_2O) δ 8.50 (d, *J* = 6.5 Hz, 4H), 7.93 (d, *J* = 6.4 Hz, 4H), 7.65 (d, *J* = 8.4 Hz, 4H), 6.98 (dd, *J* = 11.1, 8.2 Hz, 6H), 6.80 – 6.70 (m, 3H), 5.78 (d, *J* = 16.0 Hz, 1H), 4.19 (s, 6H). ^{13}C NMR (151 MHz, D_2O) δ 173.23, 154.12, 149.31, 146.11, 144.57, 139.83, 131.48, 129.21, 129.14, 127.80, 125.78, 123.65,

123.31, 122.18, 47.00. HRMS *m/z*: $[M]^{2+}$, Calcd. for $C_{33}H_{29}N_3O_2^{2+}$, 249.6124; found, 249.6120.

Synthesis of (*E*)-3-(4-(bis(4-(pyridin-4-yl)phenyl)amino)phenyl)acrylic acid (**6**): Compound **2** (400 mg) and a sodium hydroxide solution (300 mg in 5 mL of water) were added to 10 mL of MeOH, and the mixture was stirred at room temperature for 4 h. Subsequently, the solvent was removed by rotary evaporation. The residual sodium hydroxide was removed by washing with water, followed by freeze-drying. The product (6) was finally isolated by column chromatography to yield a solid (363 mg, 97%). ^{1}H NMR (400 MHz, DMSO-d_6) δ 8.65 – 8.52 (m, 4H), 7.85 – 7.74 (m, 4H), 7.71 – 7.64 (m, 4H), 7.53 (d, *J* = 8.3 Hz, 2H), 7.25 (d, *J* = 15.9 Hz, 1H), 7.22 – 7.12 (m, 4H), 7.07 (d, *J* = 8.4 Hz, 2H), 6.39 (d, *J* = 15.9 Hz, 1H). HRMS *m/z*: $[M+2H]^{2+}$ Calcd. for $C_{31}H_{25}N_3O_2^{2+}$, 235.5968; found 235.5984; $[M+H]^+$ Calcd. for $C_{31}H_{24}N_3O_2^+$, 470.1863; found 470.1922.

Synthesis of DNATplatin (**7**): Compound 5 (47 mg), synthesized according to our previous work,[2] was dissolved in 3 mL of DMF and reacted with HATU (59 mg, 1.5 eq) and DIPEA (30 μL, 1.5 eq). The mixture was stirred at room temperature for 0.5 h, followed by the addition of compound 6 (43 mg, 1.0 eq). After being stirred at 50°C for 12 h, the crude product was directly used for the next step. The crude product (50 mg) and methyl iodide (50 μL) were then dissolved in 1 mL of DMF, and the mixture was stirred at 70°C for 12 h. Subsequently, the mixture was poured into ether, and the precipitate was collected to obtain the crude product, which was subjected to preparative HPLC separation. For HPLC purification, a C18 column was used with a mobile phase gradient: A phase – 0.1% formic acid in H_2O, B phase – 0.1% formic acid in CH_3CN. The gradient was as follows: at 0.01 min, 95% A and 5% B; at 7.00 min, 51% A and 49% B; at 13.00 min, 45% A and 55% B; at 14.00 min, 95% A and 5% B; and at 18.00 min, 95% A and 5% B. Finally, the product was characterized by appropriate analytical techniques. Red solid, 60% yield. ^{1}H NMR (600 MHz, DMSO-d_6) δ 8.95 (s, 4H), 8.48 (d, 8H), 8.10 (d, J = 8.4 Hz, 4H), 7.71 (d, J = 8.2 Hz, 2H), 7.44 (d, J = 15.8 Hz, 1H), 7.28 (d, J = 8.0 Hz, 4H), 7.19 (d, J = 8.2 Hz, 2H), 6.50 (d, J = 15.9 Hz, 1H), 4.30 (s, 6H), 2.64 – 2.50 (m, 2H), 2.14 (d, J = 12.0 Hz, 2H), 1.97 (s, 3H), 1.48 (m, 4H), 1.16 (q, J = 14.3 Hz, 2H). ^{13}C NMR (151 MHz, DMSO-d_6) δ 189.85, 178.43, 174.01, 165.33, 163.61, 153.09, 149.32, 146.60, 145.47, 141.20, 131.20, 129.84, 129.78, 128.01, 125.66, 124.05, 123.12, 120.45, 69.78, 61.37, 61.18, 46.88, 30.88, 23.61, 23.54, 22.98. ^{195}Pt NMR (129 MHz, DMSO-d_6) δ 1611.05. HRMS *m/z*: $[M]^{2+}$, Calcd. for $C_{43}H_{45}N_5O_8Pt^{2+}$, 477.1452; found, 477.1448.

Cell culture

A549 (Cat#CCL-185), 4T1 (Cat#CRL-2539), and HEK293 (Cat#CRL-3216) cells were obtained from ATCC. The A549cisR cell line was derived from A549 cells following a previous protocol.[3] Human lung carcinoma A549 cells, cisplatin-resistant A549cisR cells, 4T1 cells, and HEK293 cell lines were cultured in DMEM supplemented with 10% FBS and 100 μg mL^{-1} penicillin/streptomycin.

Solubility determination *via* ICP-OES analysis

To determine solubility, the saturated aqueous solutions of various complexes (DNATplatin, oxaliplatin, HO-oxaPt(IV)-OH, and Ac-oxaPt(IV)-OH) were first centrifuged to remove any undissolved solids at laboratory temperature (22°C). Subsequently, a volume (5 μL) of each clarified supernatant was transferred into a 1.5 mL tube containing 330 μL of concentrated nitric acid for an overnight digestion. Following digestion, the mixture was diluted to 1 mL with Milli-Q water, thoroughly mixed, and the Pt content was analyzed by ICP-OES.

HPLC-based stability evaluation of DNATplatin

In PBS buffer (pH=7.4): A 90 μL of DNATplatin (55 μM) in water was mixed with 10 μL of 10× PBS. The stability of the resulting solution was evaluated over 24 h using HPLC with a gradient mobile phase consisting of (A) 0.1% formic acid in H_2O and (B) 0.1% formic acid in CH_3CN. The gradient elution program was as follows: 0-0.01 min, 95% A and 5% B; 7.00 min, 51% A and 49% B; 13.00 min, 45% A and 55% B; 14.00 min, 95% A and 5% B; 18.00 min, 95% A and 5% B.

In sodium ascorbate buffer (pH = 7.4): A 90 μL solution of DNATplatin (55 μM) in water (Solution A) was prepared. Separately, 4 mg of sodium ascorbate (final concentration 20 mM) was dissolved in 1 mL of 10 × PBS (Solution B). Then, 10 μL of Solution B was added to 90 μL of Solution A. The stability of the resulting mixture was assessed by HPLC.

HPLC analysis of photo-induced reduction

In PBS buffer (pH 7.4) supplemented with 2 mM sodium ascorbate: A 90 μL aliquot of 55 μM DNATplatin aqueous solution was mixed with 10 μL of 10 × PBS containing 20 mM sodium ascorbate. The resulting mixture was irradiated at 495 nm, and the reaction progress was monitored *via* HPLC.

In PBS buffer (pH 7.4): A 90 μL aliquot of 1 mM DNATplatin aqueous solution was mixed with 10 μL of 10 × PBS. The resulting mixture was irradiated at 495 nm, and the reaction progress was monitored *via* HPLC.

Fluorescence spectroscopy in different solvents and viscosity systems

Briefly, a defined amount of DNATplatin was dissolved in water, and the UV-Vis absorption spectrum of the stock solution was measured before it was diluted to a maximum absorbance (OD)$\leqslant$1. A first series of DNATplatin solutions with a uniform final maximum absorbance of ~0.1 (5 μM), prepared using solvent systems of 100% H_2O, 20% THF/80% H_2O, 40% THF/60% H_2O, and 80% THF/20% H_2O, was subjected to fluorescence spectroscopy. A second series of DNATplatin solutions with the same final absorbance of ~0.1, prepared using solvent systems of 100% H_2O, 20% glycerol/80% H_2O, 40% glycerol/60% H_2O, 80% glycerol/20% H_2O, and 99% glycerol/1% H_2O, was also subjected to fluorescence spectroscopy detection.

Measurement of photo-induced ROS production

A stock solution of 1,3-diphenylisobenzofuran (DPBF) was prepared by dissolving 2.7 mg of DPBF powder in 2 mL of methanol to obtain a ~5 mM DPBF solution. The stock solution was diluted with water to prepare a working solution with a maximum absorbance (OD) value of 0.6. DNATplatin was introduced into the mixture to reach a final concentration of 5 μM. The final mixed solution containing DPBF (30 μM) and DNATplatin (5 μM) was placed in a standard 1 cm quartz cuvette. The solution was then subjected to continuous light irradiation at 495 nm using a light source with an irradiance of 1 mW cm^{-2}. The UV-Vis absorption spectra of the mixture were recorded at regular intervals (every 2 minutes) to monitor the characteristic absorbance decay of DPBF. Control experiments using DPBF alone under identical light irradiation conditions were performed to calibrate the self-photolysis background.

Preparation and annealing of oligonucleotides

The oligonucleotides were purchased from Sangon (Shanghai, China) with HPLC purification and prepared by an annealing process (heating at 95°C for 5 min and then slowly cooling to room temperature overnight) in a buffer solution (10 mM Tris-HCl buffer, pH 7.4).

Determination of Pt per nucleotide binding stoichiometry

A quantity of 2 μg of dsDNA or G4 DNA was treated with various amounts of oxaliplatin or DNATplatin (6, 12, 24, 36, 42, and 48 nmol) in 50 μL of 10 mM HEPES buffer (pH 7.4, adjusted with HNO_3) for 16 h at 37°C. After incubation, unreacted Pt complexes were removed *via* ultrafiltration using a 3 kDa molecular weight cut-off membrane, followed by washing three times with 400 μL of the same HEPES buffer. The retained DNA was recovered and reconstituted to 30 μL in TE buffer (10 mM Tris-HCl, 2 mM EDTA, pH 7.4). The DNA concentration was determined using a NanoDrop UV-Vis spectrophotometer at 260 nm. Subsequently, aliquots were digested with 20% HNO_3 overnight at 65°C and Pt concentrations were ascertained by ICP-MS.

Gel electrophoresis assay for DNA photooxidation

To evaluate DNA photooxidation, 300 ng of dsDNA (3 μL) or G4 DNA was mixed with one of the following reagents in PBS buffer (pH 7.4): 2 μL of PBS, 2 μL of oxaliplatin (10 μM), 2 μL of oxaliplatin + ligand (10 μM), 2 μL of DNATplatin (10 μM), or 2 μL of H_2O_2 (10 μM). The mixture was then irradiated. Afterward, 1 μL of DNA loading buffer was added to the mixture, which was then loaded into the gel. The gel electrophoresis was run at 100 V for 20 min. In addition, we set up a gradient of the molar ratio of different base pairs in the dsDNA ladder to DNATplatin.

Determination of DNA-binding efficiency *via* ultrafiltration

A 1 mL DNA stock solution (10 μmol) and a 1 mL DNATplatin stock solution (1.0 μmol, 1 mM) were prepared in PBS. Subsequently, 10 μL of the DNATplatin solution was mixed with 4.2 μL of the DNA stock solution (0.42 eq.), and the mixture was diluted with PBS to a final volume of 1 mL. The resulting solution was subjected to

ultrafiltration using a 3 kDa molecular weight cut-off (MWCO) filter tube. Finally, the UV-Vis absorbance of both the retentate (post-ultrafiltration solution) and the filtrate (ultrafiltration liquid) was measured.

Fluorescence response triggered by dsDNA and G4 DNA anchoring

A 1 mL DNA stock solution (1.0 μmol, 1 mM) and a 1 mL DNATplatin or ligand stock solution (1 μmol, 1 mM) were prepared in PBS. Subsequently, 10 μL of the DNATplatin stock solution was mixed with varying volumes of the DNA stock solution, and each mixture was diluted with PBS to a final volume of 1 mL. For both dsDNA and G4 DNA, the added volumes of DNA stock solution and their corresponding molar equivalents (relative to DNATplatin) were: 1 μL (0.10 eq.), 2.1 μL (0.21 eq.), 4.2 μL (0.42 eq.), 8.5 μL (0.85 eq.), 17 μL (1.70 eq.), 34 μL (3.40 eq.), 68 μL (6.80 eq.), and 101 μL (10.10 eq.). Finally, the fluorescence intensity of the resulting mixtures was recorded. The experimental fluorescence intensity F data as a function of DNA concentration [DNA] were quantified using nonlinear regression analysis based on the following specific mathematical models:

For dsDNA: The titration profiles fitted well to a standard 1:1 saturation binding model (Eq. 1):

$$F = F_0 + \frac{(Fmax - F_0) \cdot [dsDNA]}{K_D + [dsDNA]}$$

For G4 DNA: The titration profiles displayed a pronounced sigmoidal character, signifying multi-site binding with cooperative character. Consequently, the data were fitted using the Hill equation (Eq. 2):

$$F = F_0 + \frac{(Fmax - F_0) \cdot [G4\ DNA]^n}{K_D + [G4DNA]^n}$$

Where F_0 is the initial fluorescence intensity, $Fmax$ is the maximum intensity at complete saturation, K_D is the dissociation constant, and n represents the Hill coefficient (binding stoichiometry/cooperativity). The association constants K_a, in (M^{-1}) were calculated *via* ($K_a = 1 / (K_D \times 10^{-9})$).

Confocal fluorescence cellular imaging

A549 and 4T1 cells were seeded into a glass-bottom dish at a density of 4×10^3 cells per dish and incubated with 10 μM DNATplatin for 6 h. After removing the culture medium, the cells were fixed with 4% paraformaldehyde solution and stained with ~1 µg mL^{-1} DAPI (or Hoechst). Finally, cell images were captured using a CLSM (Leica SP5 equipped with an IR laser).

Molecular Docking Studies

Molecular docking was performed using AutoDock 4.2 to investigate the non-covalent interactions between DNATplatin and DNA (PDB IDs: 6KFJ and 5JU4). Water molecules were removed, polar hydrogens were added, and Kollman charges were assigned to the DNA using AutoDockTools. The structure of DNATplatin was optimized, and Gasteiger charges were assigned. Conformations were searched using the Lamarckian Genetic Algorithm over 100 independent runs. The optimal binding poses were selected based on the lowest binding free energies.

DFT calculations

Software: Gaussian 16 RevC.01

B3LYP/def2-SVP (SCRF, solvent = water) for geometry optimization and frequency calculation.

B3LYP (SCRF, solvent = water)/def2-TZVP for energy calculation (energy = E2)

TD-B3LYP (SCRF, solvent = water)/def2-TZVP for energy calculation of the excited states based on the optimized ground state geometry.

MTT assay

A549, A549cisR, 4T1, and HEK293 cells were seeded into 96-well plates at a density of 4×10^3 cells per well and incubated for 48 h. The cells were treated with varying concentrations of oxaliplatin, ligand, or DNATplatin (50, 10, 2, 0.4, and 0.04 μM) for 8 h. Two plates were then transferred to phenol red-free medium; one plate was irradiated with 495 nm light (1 mW cm^{-2}) for 30 min. After replacing the medium with complete medium, the cells were incubated for another 64 h. Following incubation, the culture medium was aspirated, and MTT solution was added for 4 h. Finally, the medium was removed, and 100 μL of DMSO was added to all wells.

Cellular uptake of DNATplatin detected by Flow cytometry

A549, A549cisR, and 4T1 cells were seeded into 24-well plates at a density of 2×10^4 cells per well. After incubation with 10 μM DNATplatin for various time intervals (1, 4, 7, 8, and 9 h), the culture medium was aspirated. The cells were then harvested and collected for subsequent flow cytometry measurements.

Cellular uptake of DNATplatin detected by CLSM

A549, A549cisR, and 4T1 cells were seeded into glass-bottom dishes at a density of 2×10^4 cells per dish. The cells were incubated with 10 μM DNATplatin for 1, 4, 7, and 8 h, and then the culture medium was removed. Afterward, the cells were fixed with 4% paraformaldehyde and stained with Hoechst (~1 µg mL^{-1}). Finally, fluorescence images were captured using a Leica SPE confocal laser scanning microscope.

Cellular uptake of DNATplatin detected by ICP-MS

4T1 cells were seeded into 6-well plates at a density of 2×10^4 cells per well. The cells were incubated with 10 μM oxaliplatin, oxaliplatin+ligand, or DNATplatin for various time periods (1, 2, 4, 6, 8, 10, 12, and 24 h). Afterward, the culture medium was removed, and the cells were washed twice with PBS. The cells were then harvested into 2 mL tubes and recounted. The resulting cell pellets were digested overnight at 50°C with a mixture of 65% HNO_3 (0.33 mL) and 15% H_2O_2. Finally, the cell lysates were diluted with Milli-Q water to a final volume of 1 mL for ICP-MS measurements.

Circular dichroism study on the binding of G4 DNA to DNATplatin

Preparation of 1 mL G4 DNA stock solution in PBS (containing 10 nmol DNA) was carried out. A 50 μL DNATplatin PBS stock solution (containing 5 μmol DNATplatin at 5 mM) was then prepared. Titration of 1–8 μL DNATplatin stock solution into the G4 DNA stock solution yielded complexes with DNATplatin-to-G4 DNA molar ratios ranging from 1:1 to higher ratios. A similar titration procedure was performed for dsDNA, achieving the corresponding DNATplatin-to-dsDNA molar ratios.

For circular dichroism spectroscopy, preparation of 1 mL G4 DNA or dsDNA stock PBS solutions (10 nmol DNA content) was performed. A 50 μL DNATplatin PBS stock solution (5 μmol total, 5 mM concentration) was prepared. Addition of 6 μL DNATplatin stock solution (3:1 molar ratio to DNA) to the DNA stock solution was carried out. Irradiation of the resulting mixture for 30 min was then conducted. Subsequent circular dichroism spectroscopy measurements on the irradiated samples were performed.

Intracellular ROS detection

4T1 cells were seeded into glass-bottom dishes at a density of 1 × 10^6 cells per dish and incubated for 6 h with either 10 μM DNATplatin, 10 μM ligand, or 10 μM oxaliplatin. For the positive control group, cells were incubated with 100 μM Menadione for 1 h, while the blank control group was maintained in complete medium without any treatment and served as the negative control. After treatment, the culture medium was removed, and 5 μM H_2DCFDA was added to the dishes and incubated for 30 min. Following incubation, the staining solution was removed and the dishes were washed three times with PBS. The cells were then irradiated with 495 nm light at an irradiance of 1 mW cm^{-2} for 2 min. After irradiation, the cells were fixed with 4% paraformaldehyde solution, and cell images were collected using CLSM.

Quantification of Pt in genomic DNA

4T1 cells were seeded into a 6-well plate at a density of 1 × 10^6 cells per well and incubated for 24 h. After incubation, the cells were treated with 2 μM or 5 μM oxaliplatin or DNATplatin (n = 3) for 6 h. Following treatment, the culture medium was removed and the cells were washed twice. After washing, the cells were counted again and 1×10^6 cells were collected into a 2 mL centrifuge tube. Genomic DNA was extracted from the collected cells following the manufacturer's protocol of the extraction kit. Both the extracted DNA samples and control cell samples were digested with 0.33 mL of 65% nitric acid and 15% H_2O_2 overnight at 50°C. After digestion, the samples were diluted to a final volume of 1 mL for ICP-MS analysis.

$$\text{DNA} - targeting\ efficiency = \frac{Pt\ content\ in\ the\ genomic\ \text{DNA}}{Pt\ content\ in\ total\ cell}$$

Quantification of Pt in 3D spheroids

4T1 cells were seeded into ultra-low attachment plates at a density of 1 × 10^3 cells per well. The cells were then incubated for approximately 7 days until they formed spheroids with a diameter of approximately 600 μm. The formed spheroids were subsequently incubated with 10 μM DNATplatin or ligand for 10 h.

After incubation with the respective compounds, the spheroids were transferred to glass-bottom dishes. They were then washed three times with PBS to remove any residual compounds. Following washing, confocal imaging was performed on the spheroids to observe their morphology and fluorescence signals. The platinum content within the 3D spheroids was determined using ICP-MS.

Live and dead assay

4T1 cells were seeded into ultra-low attachment plates at a density of 1 × 10^3 cells per well and cultured for 7 days until 3D tumor spheroids were formed. The mature spheroids were incubated with 10 μM DNATplatin, ligand, oxaliplatin, or PBS for 6 h. After incubation, the culture medium was replaced with phenol red-free medium. The spheroids were irradiated at 495 nm with an irradiance of 1 mW cm^{-2} for 30 min. Following irradiation, the medium was replaced with complete culture medium and the spheroids were further incubated for an additional 24 h. The spheroids were then stained with a mixed staining solution containing 5 μM DAPI and 5 μM Calcein AM for 20 min. After staining, the spheroids were transferred to glass-bottom dishes, washed three times with PBS, and subjected to confocal imaging.

Immunofluorescence staining for HMGB1

4T1 cells were seeded into glass-bottom dishes at a density of 1 × 10^5 cells per well and cultured for 24 h. Afterward, the cells were incubated with oxaliplatin (2 or 100 μM), ligand (2 μM), or DNATplatin (2 μM) for 6 h. Following incubation, the cells were washed three times with PBS, then irradiated with or without light (495 nm, 1 mW cm^{-2}) for 30 min, and incubated for an additional 24 h. After the second incubation, the cells were washed three times with PBS and fixed with 4% paraformaldehyde (PFA) in PBS solution for 10 min. The cells were then washed again with PBS three times, permeabilized with 0.25% Triton X-100 for 10 min, and blocked with 5% bovine serum albumin (BSA) solution at 37°C for 30 min. The cells were incubated with the primary antibody (anti-HMGB1, diluted 1:200) for 90 min at 37°C, washed three times with PBS, and then incubated with the FITC-conjugated secondary antibody (diluted 1:200) at 37°C for 60 min. After washing the cells three times with PBS, confocal imaging was performed.

Immunofluorescence staining for CRT

4T1 cells were seeded into glass-bottom dishes at a density of 1 × 10^5 cells per well and cultured for 24 h. Afterward, the cells were incubated with oxaliplatin (2 μM or 100 μM) or DNATplatin (2 μM) for 6 h. Following incubation, the cells were washed three times with PBS, irradiated with or without light (495 nm, 1 mW cm^{-2}) for 30 min, and

incubated for an additional 24 h. After the second incubation, the cells were washed three times with PBS and fixed with 4% paraformaldehyde (PFA) in PBS solution for 10 min. The cells were then washed three more times with PBS, permeabilized with 0.25% Triton X-100 for 10 min, and blocked with 5% bovine serum albumin (BSA) solution at 37°C for 30 min. The cells were incubated with the primary antibody (anti-CRT, diluted 1:200) for 90 min at 37°C, washed three times with PBS, and then incubated with the FITC-conjugated secondary antibody (diluted 1:200) at 37°C for 60 min. After washing the cells three times with PBS, confocal imaging was performed.

Measurement of ATP release

4T1 cells were seeded into glass-bottom dishes at a density of 1×10^5 cells per well and cultured for 24 h. Afterward, the cells were incubated with oxaliplatin or DNATplatin (2 μM) for 6 h. Following incubation, the cells were washed three times with PBS, irradiated with or without light (495 nm, 1 mW cm^{-2}) for 30 min in phenol red-free medium, and incubated for an additional 24 h. After the second incubation, the ATP content in the medium was determined using an ATP assay kit.

RNA-Seq analysis of G4-related and marker gene expression after drug treatment

4T1 cells (1×10^5 cells/well) were seeded into 6-well plates and cultured for 24 h. Afterward, the cells were incubated with oxaliplatin (2 μM), DNATplatin (2 μM), or PDS (positive control for stable G4) for 6 h. Following incubation, the cells were washed three times with PBS. For the DNATplatin-treated groups, the cells were irradiated with or without light (495 nm, 1 mW cm^{-2}) for 30 min and incubated for an additional 24 h. After the second incubation, the cells were collected and frozen for RNA-Seq analysis. RNA-Seq was performed by Novo Gene.

Real-time quantitative PCR (RT-qPCR)

RT-qPCR experiments were performed in the 4T1 cell line. 4T1 cells (1×10^5 cells) were seeded in 6-well dishes for 24 h and treated with oxaliplatin+ligand (2 μM), DNATplatin (2 μM), oxaliplatin (2 μM), or pyridostatin (2 μM) for 6 h. After replacing with fresh medium, cells in the oxaliplatin+ligand and DNATplatin groups were either exposed to light (495 nm, 1 mW cm^{-2}) or kept in the dark for 30 min. Total RNA was isolated using the Beyotime RNA Purification Kit. Subsequently, genomic DNA removal and reverse transcription were performed in a single step using the Color All-in-one Reverse Transcription Kit. The qPCR assay was prepared by mixing the cDNA template, specific primers, and nuclease-free water with the color qPCR Master Mix. Real-time quantitative PCR (RT-qPCR) was executed in triplicate on a CFX96 Real-Time PCR Detection System (Bio-Rad Laboratories, Hercules, CA, USA). The following mouse-specific primers were used: c-*Myc* (forward: 5'-GCGACTCTGAAGAAGAGCAAG-3'; reverse: 5'-GCCTCGGGATGGAGATGAG-3'), *Vegfr-1* (forward: 5'-TGGCTCACAGGCAACATCGTGA-3'; reverse: 5'-GGTGATGTATGGTCTTCGCAAG-3'), and *Gapdh* (forward: 5'-AGGTCGGTGTGAACGGATTTG-3'; reverse: 5'-TGTAGACCATGTAGTTGAGGTCA-3'). Melting curves

were used to detect and verify the specificity of PCR products. The $2^{-\Delta\Delta ct}$ method was used to calculate relative gene expression levels, where the amounts of *Myc* mRNA and *Vegfr-1* mRNA were normalized to an endogenous reference *Gapdh*, and C_t represents the threshold cycle.

Western blot

4T1 cells (2×10^5 cells) were seeded in 6-well dishes for 24 h and treated with oxaliplatin+ligand (2 μM), DNATplatin (2 μM), oxaliplatin (2 μM), or pyridostatin (2 μM) for 6 h. After replacing with fresh medium, cells in the oxaliplatin+ligand and DNATplatin groups were either exposed to light (495 nm, 1 mW cm^{-2}) or kept in the dark for 30 min. All groups were then incubated for another 24 h before analysis. Cells were washed with cold PBS, lysed, and quantified by a BCA assay. Samples were mixed with Laemmli buffer. Whole-cell lysates were separated by SDS-PAGE at 70 V for 150 min and transferred to PVDF membranes at 120 V for 60 min. Membranes were blocked with 5% nonfat milk in TBST for 2 h at room temperature and incubated with primary antibodies against VEGFR1, c-MYC, and GAPDH overnight at 4°C. After washing with 4% milk-TBS 3–5 times, membranes were incubated with secondary antibody for 1 h and washed with TBST 3 times. Signals were developed with ECL reagent (Bio-Rad) for 10 min and captured on a Bio-Rad ChemiDoc™ Touch Imaging System.

Ethical statement

All mice were purchased from the City University of Hong Kong and maintained under pathogen-free conditions. All animal experiments were conducted in accordance with the City University of Hong Kong's guidelines for the care and use of laboratory animals, with approval from the university's animal ethics committee. (reference number: AN-STA-00000308).

Determination of maximum tolerated dose

Three mice per group received a single dose of DNATplatin *via* the tail vein at the following doses: 3.5 mg Pt kg^{-1} body weight, 2.5 mg Pt kg^{-1} body weight, or 1.5 mg Pt kg^{-1} body weight.

Hemolysis assay

Blood was collected from BALB/c mice into an anticoagulant tube. A 400 μL aliquot of whole blood was transferred to a 1.5 mL EP tube, mixed with 800 μL of saline, and centrifuged at 3000 rpm for 10 min. The supernatant was discarded, and the cells were resuspended. This washing process was repeated three times until the supernatant became colorless. A 2% red blood cell suspension was then prepared by mixing 500 μL of the washed blood cells with 25 mL of saline. To 950 μL of this suspension, 50 μL of samples (oxaliplatin, ligand, DNATplatin, or oxaliplatin+ligand) at a concentration of 50 nM was added, and the mixture was incubated at 37°C for 2 h. After incubation, the mixture was centrifuged at 3000 rpm for 10 min, and the supernatant was transferred to a 96-well

plate. Absorbance was measured at 542 nm using a microplate reader, and images were taken before the supernatant transfer.

$$Hemolysis\ ratio = \frac{OD\ (sample) - OD\ (saline)}{OD\ (Triton\ X-100) - OD\ (Saline)}$$

Establishment of 4T1 breast tumor model

A total of 1×10^7 4T1 cells were dispersed in 100 μL of PBS and subcutaneously implanted into 6-week-old female BALB/c mice to establish a tumor-bearing model. One-week post-implantation, the tumors grew to approximately 100 mm^3 in size.

Biodistribution of fluorescent DNATplatin

A 4T1 tumor-bearing mouse with a tumor volume of approximately 300 mm^3 was selected and injected via the tail vein with DNATplatin at a dose of 3.5 mg Pt kg^{-1} body weight. The Pt concentration was determined using ICP-OES. Fluorescence changes in the tumor were monitored at multiple time points post-injection (0.5 h, 3.5 h, 6 h, 12 h, and 24 h), after which the mice were sacrificed and the internal organs were imaged *ex vivo*. (λ_{ex} = 495 nm, λ_{em}= 680 nm)

Detection of antigen-presenting cells and effector T cells

Tumor tissue (with a diameter of 0.3 cm to 1.0 cm) was collected and washed in ice-cold PBS. The tissue was then placed in 5 mL of digestion solution (complete medium without penicillin-streptomycin, 1 mg mL^{-1} collagenase IV, and 100 μg mL^{-1} DNase I) and minced with scissors until it could be pipetted with a 1 mL pipette without using an extended blue tip. The tissue was digested at 37°C for 45 min. If digestion was incomplete, the process was continued. After digestion, the reaction was stopped by adding PBS containing 1% FBS (twice the volume of the digestion solution). The mixture was centrifuged at 3000 rpm for 5 min, and the supernatant was discarded. The cells were resuspended in 1 mL of PBS in a 1.5 mL EP tube, counted, and 4×10^6 cells per sample were obtained. The cells were then centrifuged at 3000 rpm for 5 min, and the supernatant was discarded to collect the cell pellet. Fluorescent antibodies for APCs (APC-CD11c, CD86-PE, and CD80-FITC) or effector T cells (CD3-APC, CD8-PE, and CD4-FITC) were added at a 1:100 dilution (total volume 50 μL), mixed by pipetting, and stained at 4°C for 30 min. Afterward, 200 μL of 1% FBS was added, the mixture was centrifuged, and the supernatant was discarded. The cells were resuspended in 200 μL of FACs buffer (PBS containing 0.5% FBS) and analyzed by flow cytometry.

Detection of $Foxp3^+$ Treg cells

After preparing a single-cell suspension, 200 μL of PBS was added, followed by centrifugation at 3000 rpm for 5 min. The supernatant was discarded, and 200 μL of permeabilization fixative (50 μL black + 150 μL white) was added. The cells were stained for 20 min at 4°C. Following staining, 200 μL of 1X wash buffer was added, and the

cells were centrifuged at 5500 rpm for 5 min. The supernatant was discarded, and intracellular staining was performed with 50 μL of intracellular antibody (Foxp3) for 30 min at 4°C. After staining, 200 μL of 1X antibody diluent buffer was added, the cells were centrifuged at 3000 rpm for 5 min, and the supernatant was discarded. Finally, the cells were resuspended in 200 μL of FACs buffer (PBS containing 0.5% FBS) and analyzed by flow cytometry.

***In vivo* antitumor efficacy evaluation**

When the subcutaneous 4T1 tumors reached approximately 100 mm^3, the mice were evenly divided into six groups, with five mice per group. The drugs were administered *via* intravenous injection every two days for a total of three injections. The treatment groups were: 1) Saline (200 μL), 2) oxaliplatin (3.5 mg Pt kg^{-1} body weight), 3) oxaliplatin + ligand (3.5 mg Pt kg^{-1} body weight), 4) oxaliplatin + ligand + laser (3.5 mg Pt kg^{-1} body weight), 5) DNATplatin (3.5 mg Pt kg^{-1} body weight), and 6) DNATplatin + laser (3.5 mg Pt kg^{-1} body weight). Laser treatment was performed 3.5 h after the drug was intravenously injected into the mice. Body weight and tumor volume were recorded every other day. Tumor volume was calculated using the formula: Tumor volume = $1/2 \times LW^2$, where "L" is the long diameter and "W" is the short diameter of the tumor. The relative tumor volume (Vt/V0) was calculated, where Vt is the mean tumor volume at a specific time point and V0 is the mean initial tumor volume.

Statistical analysis

All results were expressed as mean ± SD where applicable. GraphPad Prism 8 software (GraphPad Software) and OriginPro 2017 (OriginLab) were used for statistical analysis *$p < 0.05$; **$p < 0.01$; ***$p < 0.001$.

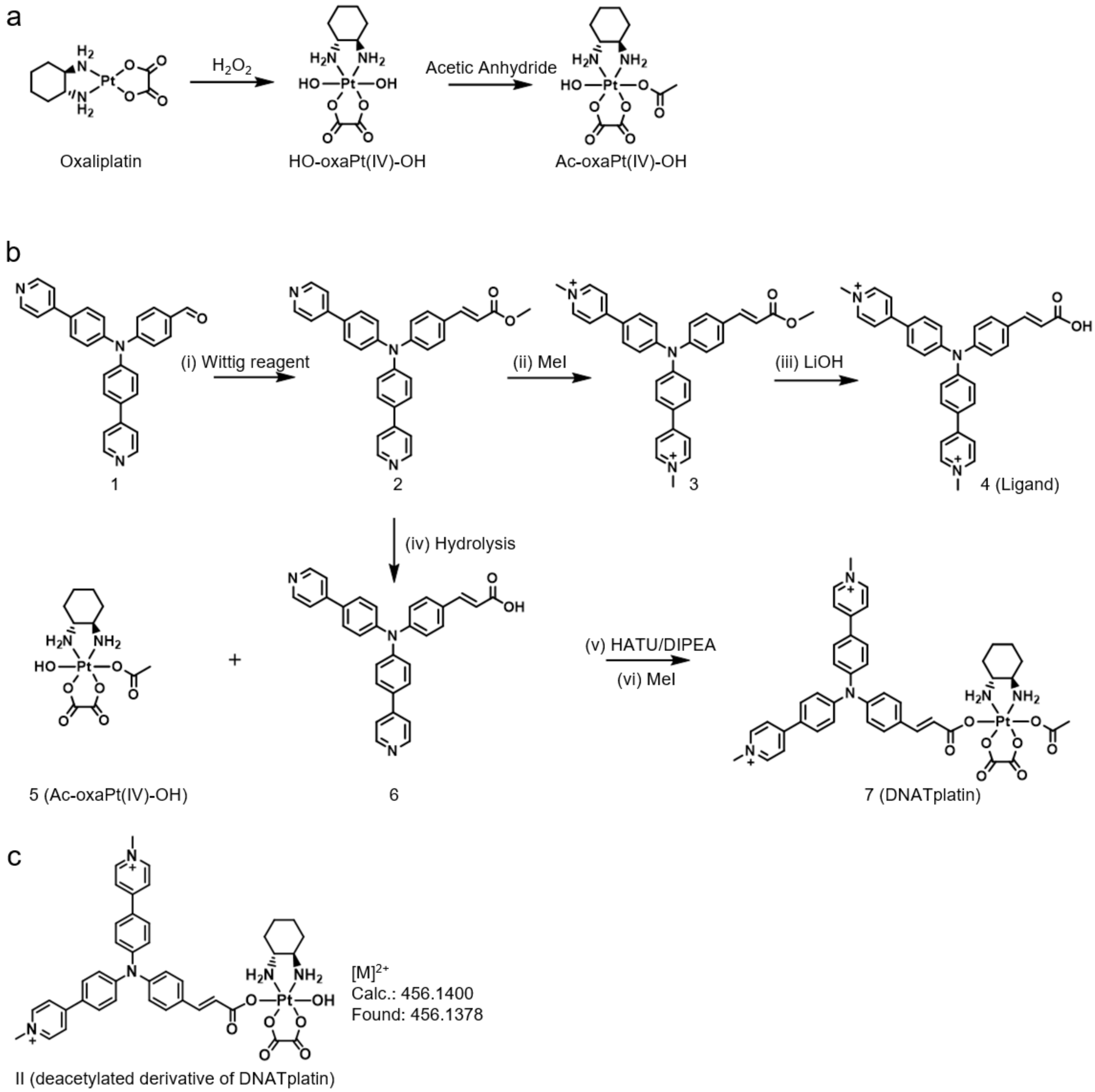

Scheme S1. (**a**) Synthesis of Ac-oxaPt(IV)-OH following previously reported methods.[2] (**b**) Synthetic routes for DNATplatin, ligand, and reagents required for each reaction. (i) Methyl(triphenylphosphoranylidene)acetate, DMF; (ii) Methyl iodide, DMF; (iii) LiOH, H_2O, MeOH; (iv) LiOH, H_2O, MeOH; (v) HATU, DIPEA, DMF; (vi) Iodomethane, DMF. (**c**) Chemical structure of peak II in the main text of Figure 1h (deacetylated derivative of DNATplatin), $[M]^{2+}$ Calc.: 456.1400; Found: 456.1378.

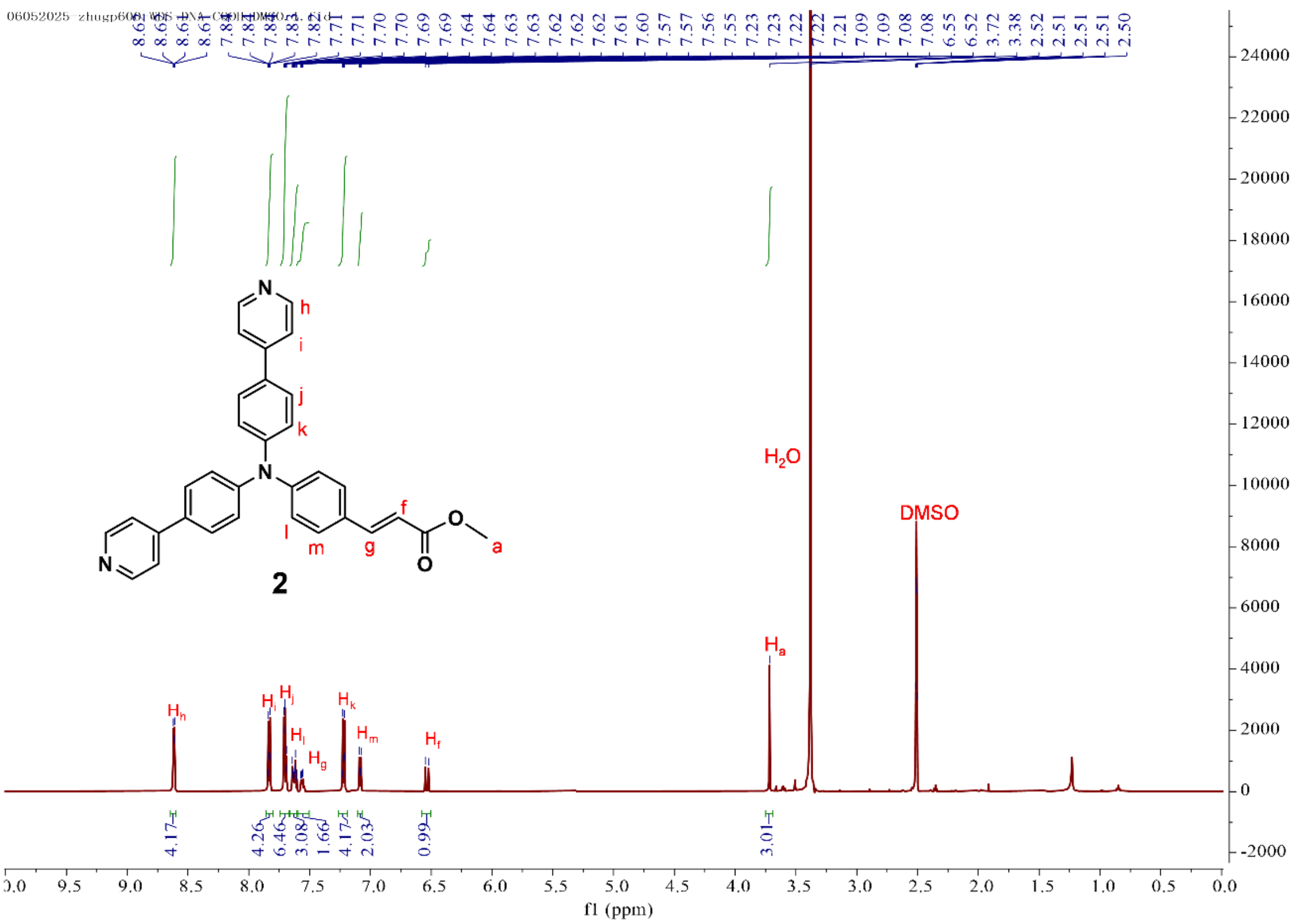

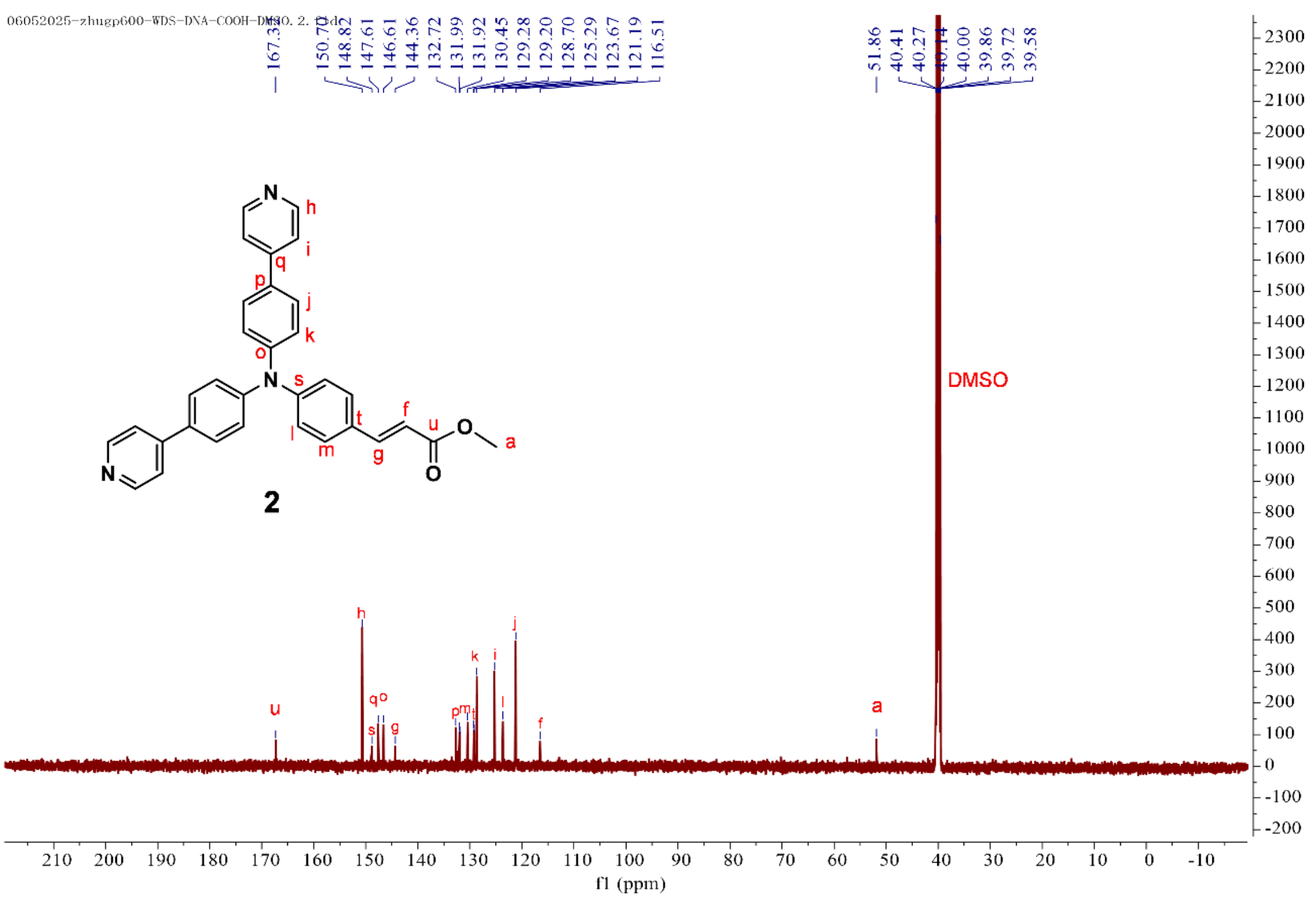

Figure S1. ^{1}H NMR and ^{13}C NMR spectra of compound **2** in DMSO-d_6.

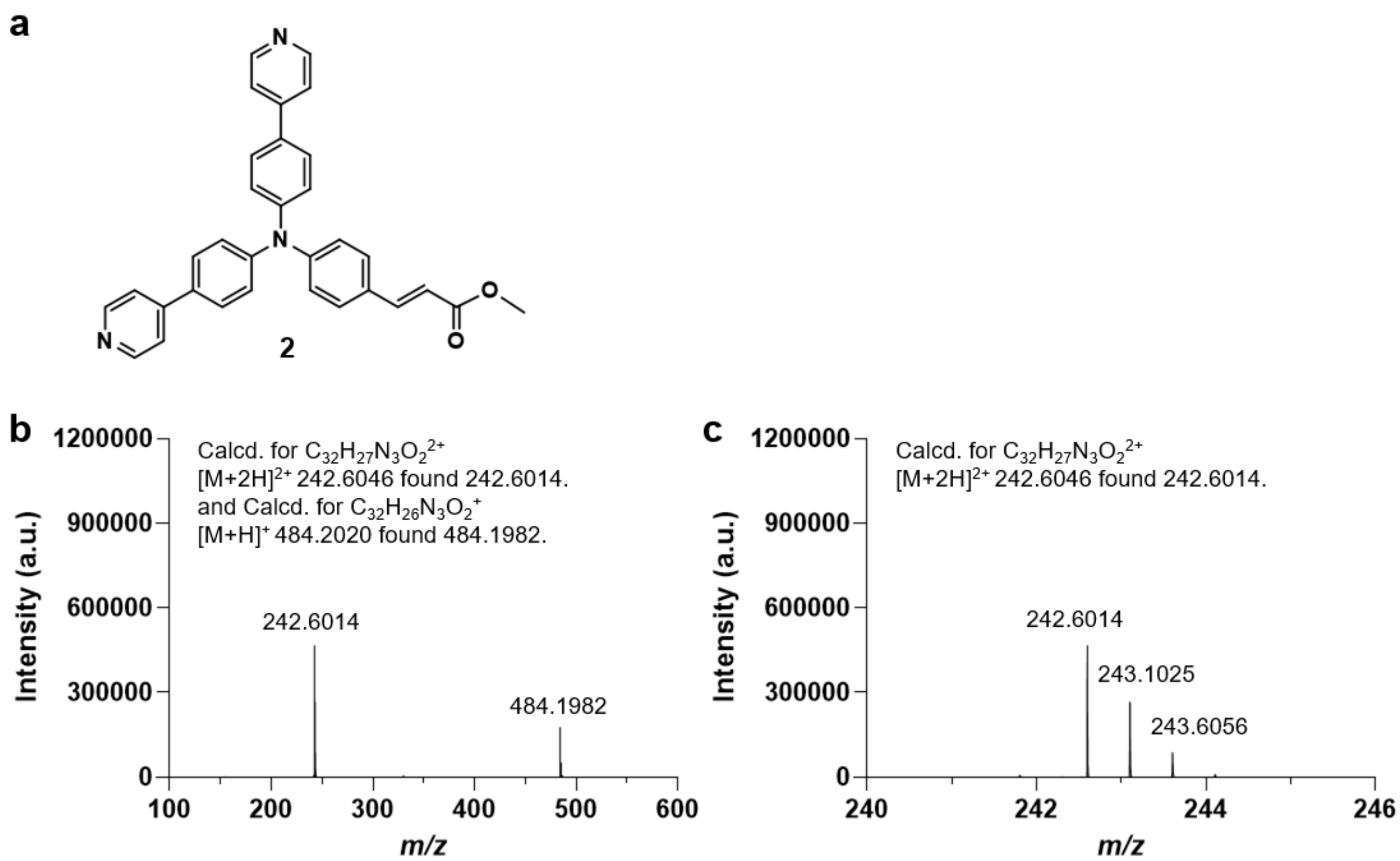

Figure S2. Chemical structure (a) and ESI-HRMS spectra (b-c) of compound **2**.

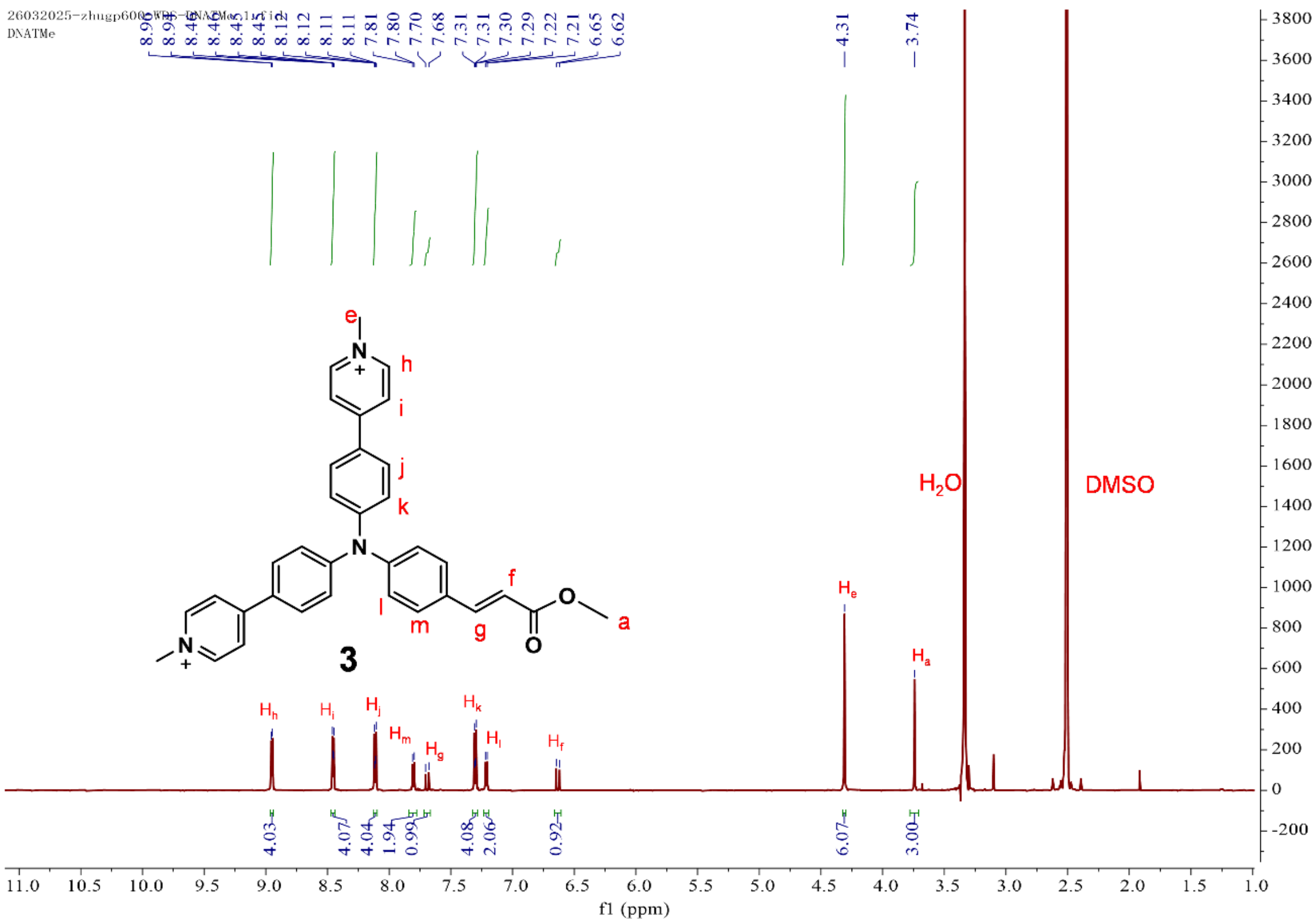

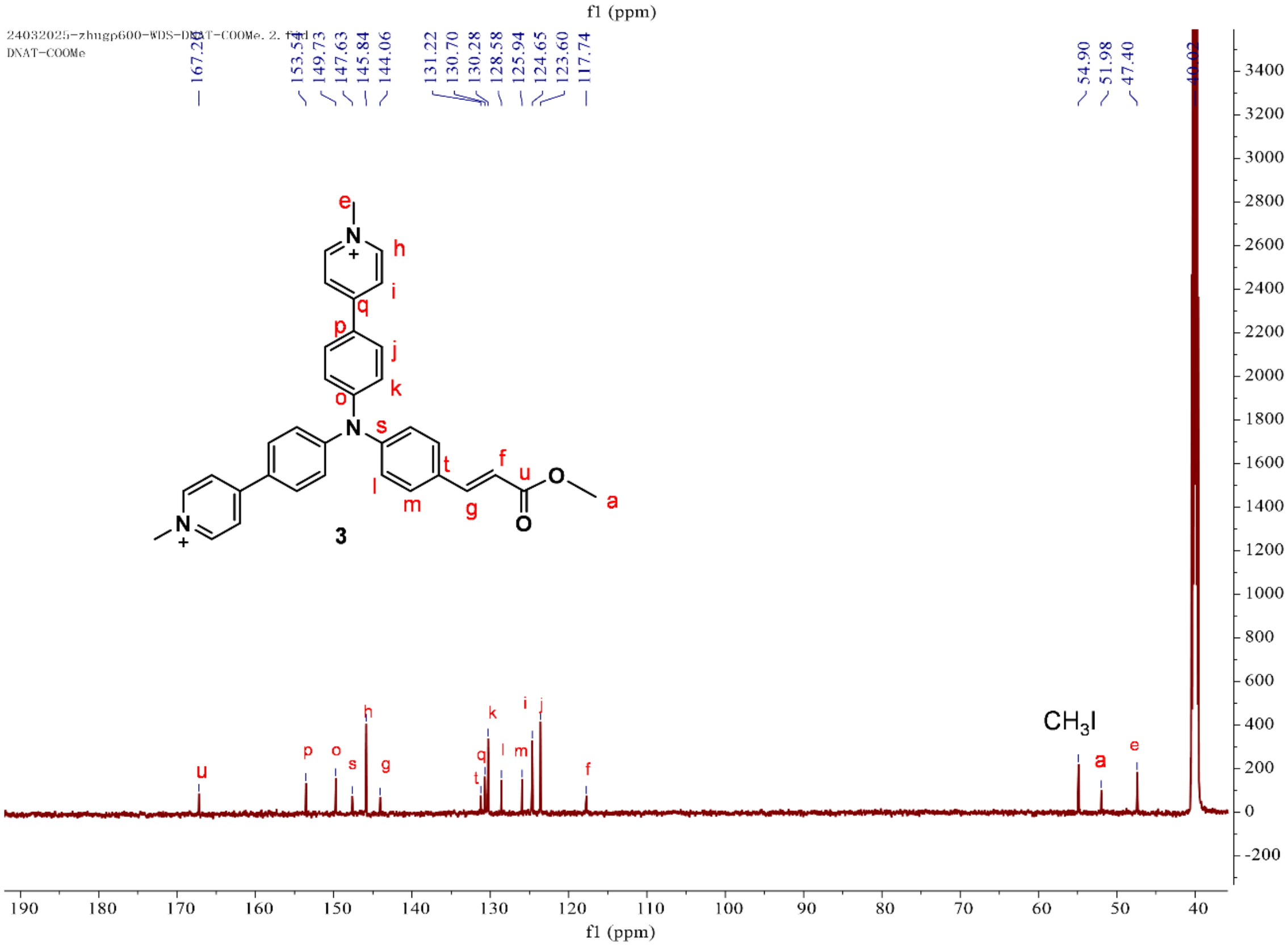

Figure S3. ^{1}H NMR and ^{13}C NMR spectra of compound **3** in DMSO-d_6.

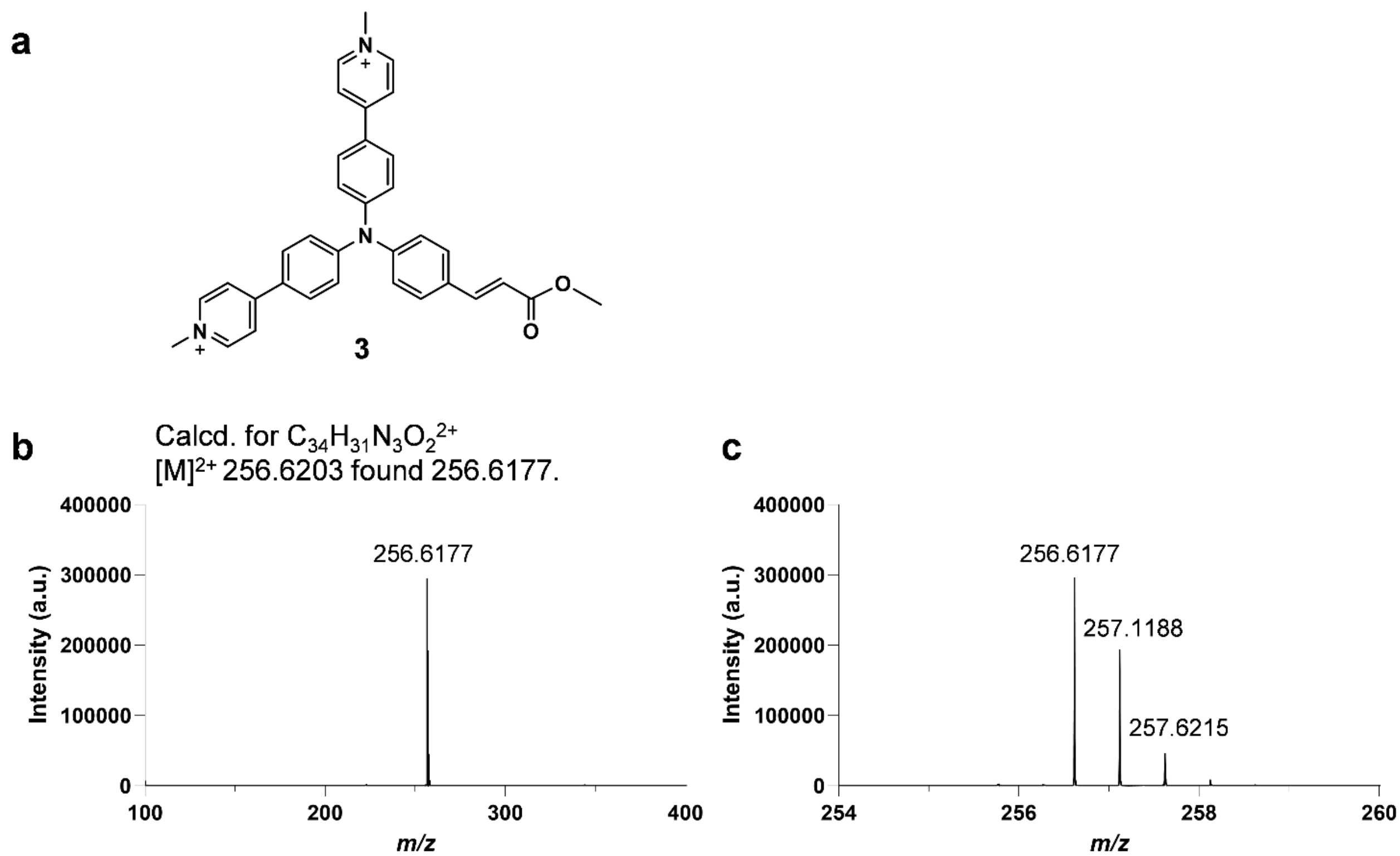

Figure S4. Chemical structure (a) and ESI-HRMS spectra (b, c) of compound **3**.

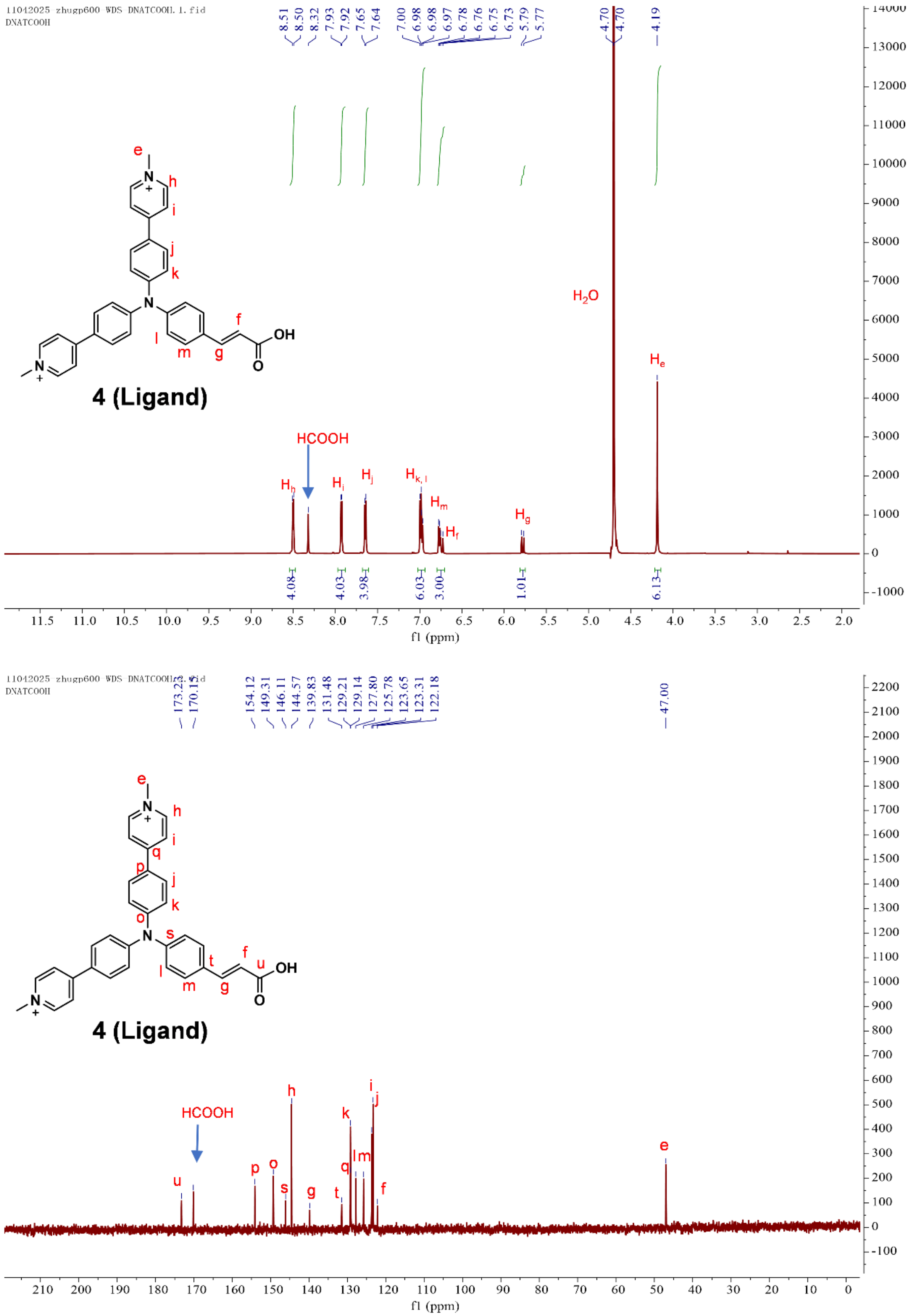

Figure S5. ^{1}H NMR and ^{13}C NMR spectra of compound **4** in D_2O

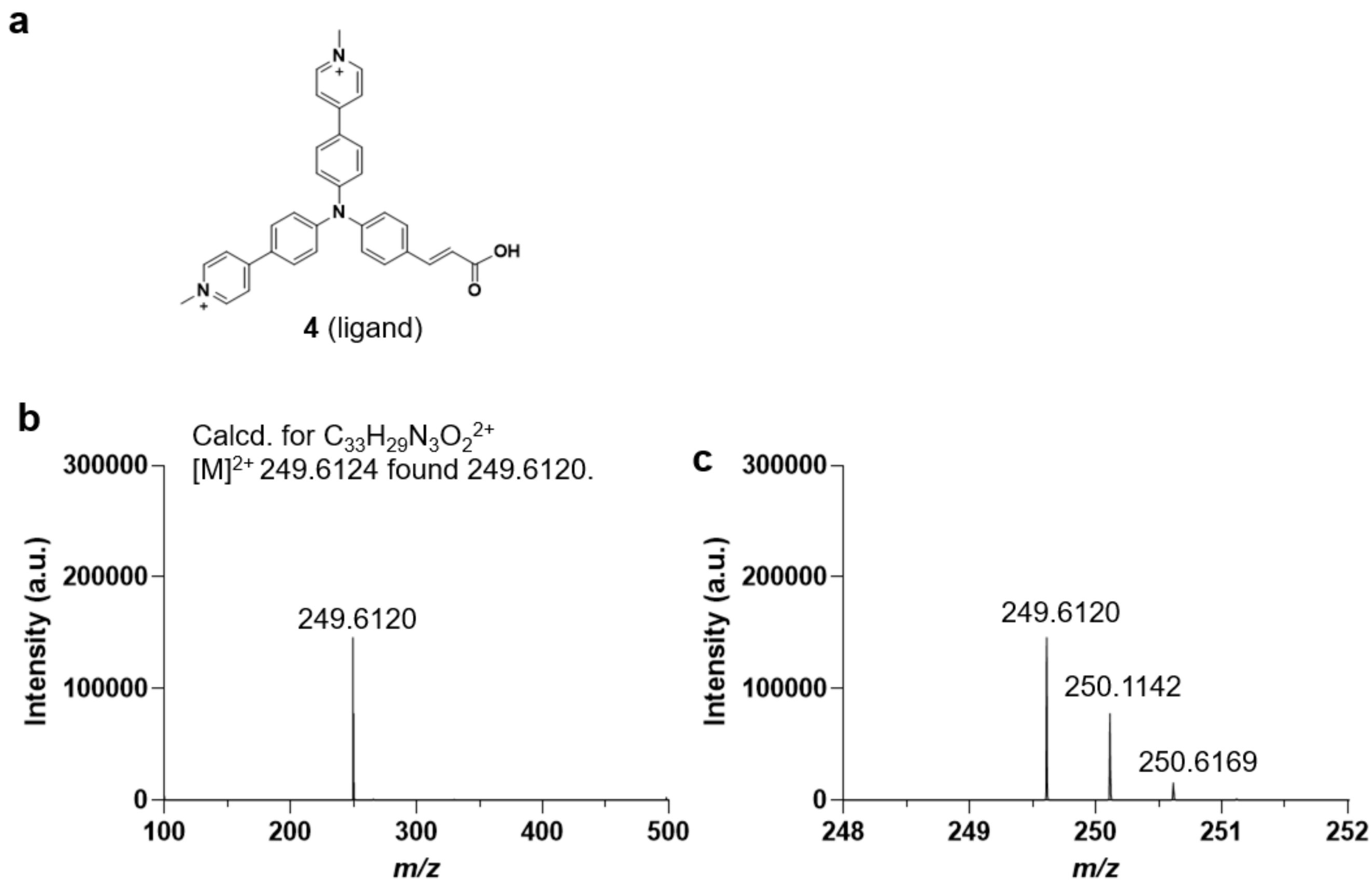

Figure S6. Chemical structure and ESI-HRMS spectra of ligand **4**.

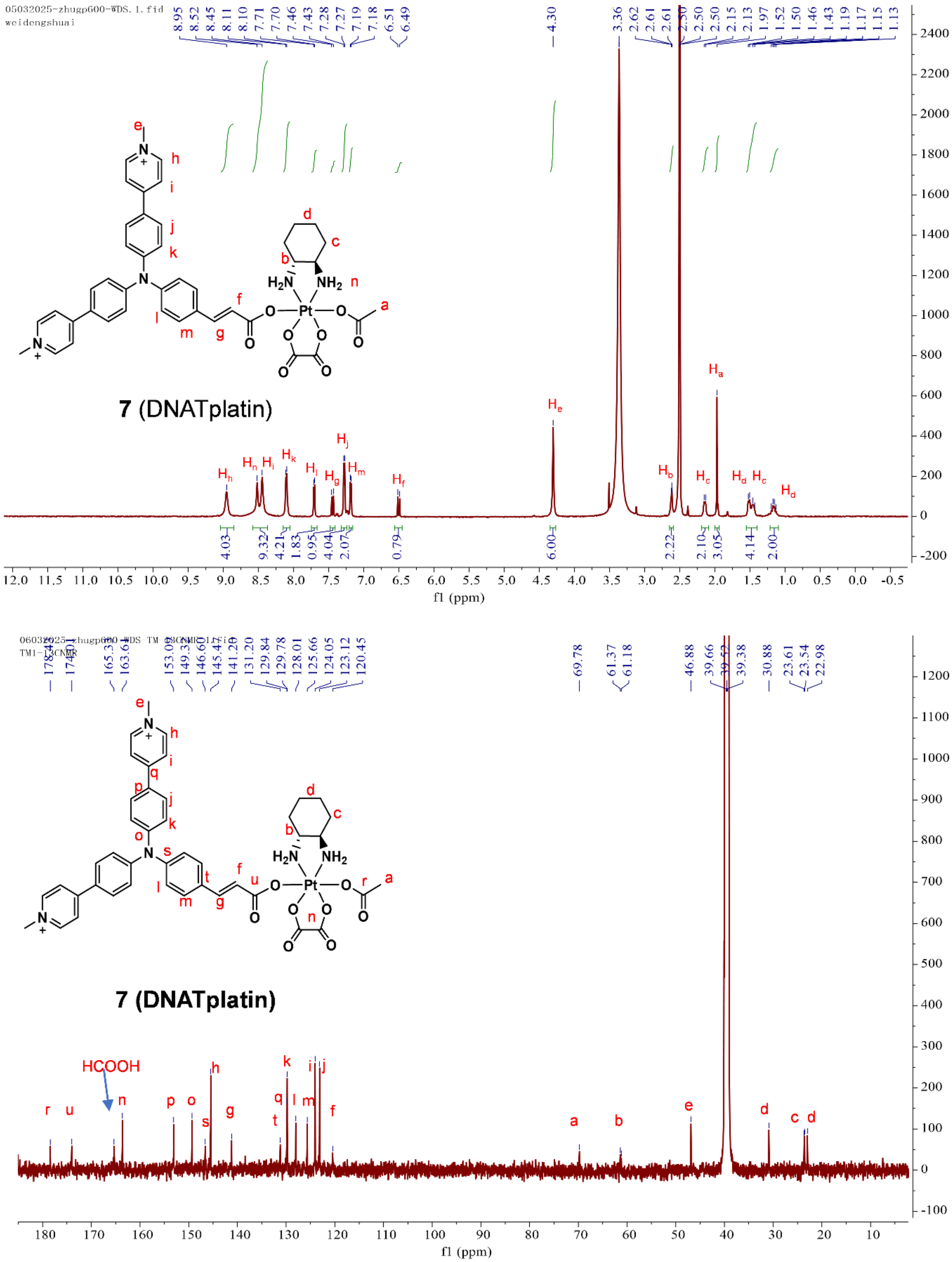

Figure S7. ^{1}H NMR and ^{13}C NMR spectra of complex **7** in DMSO-d_6.

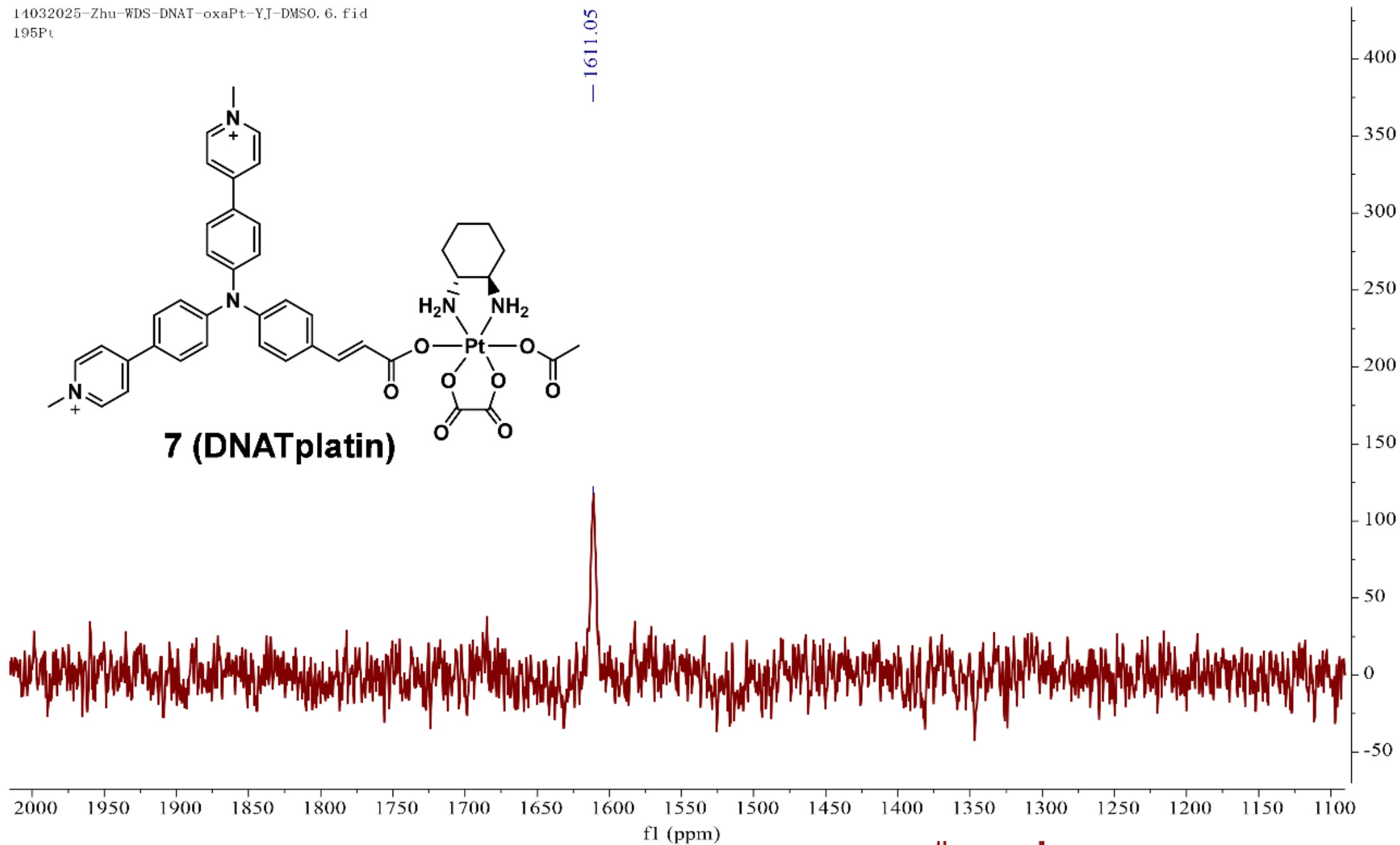

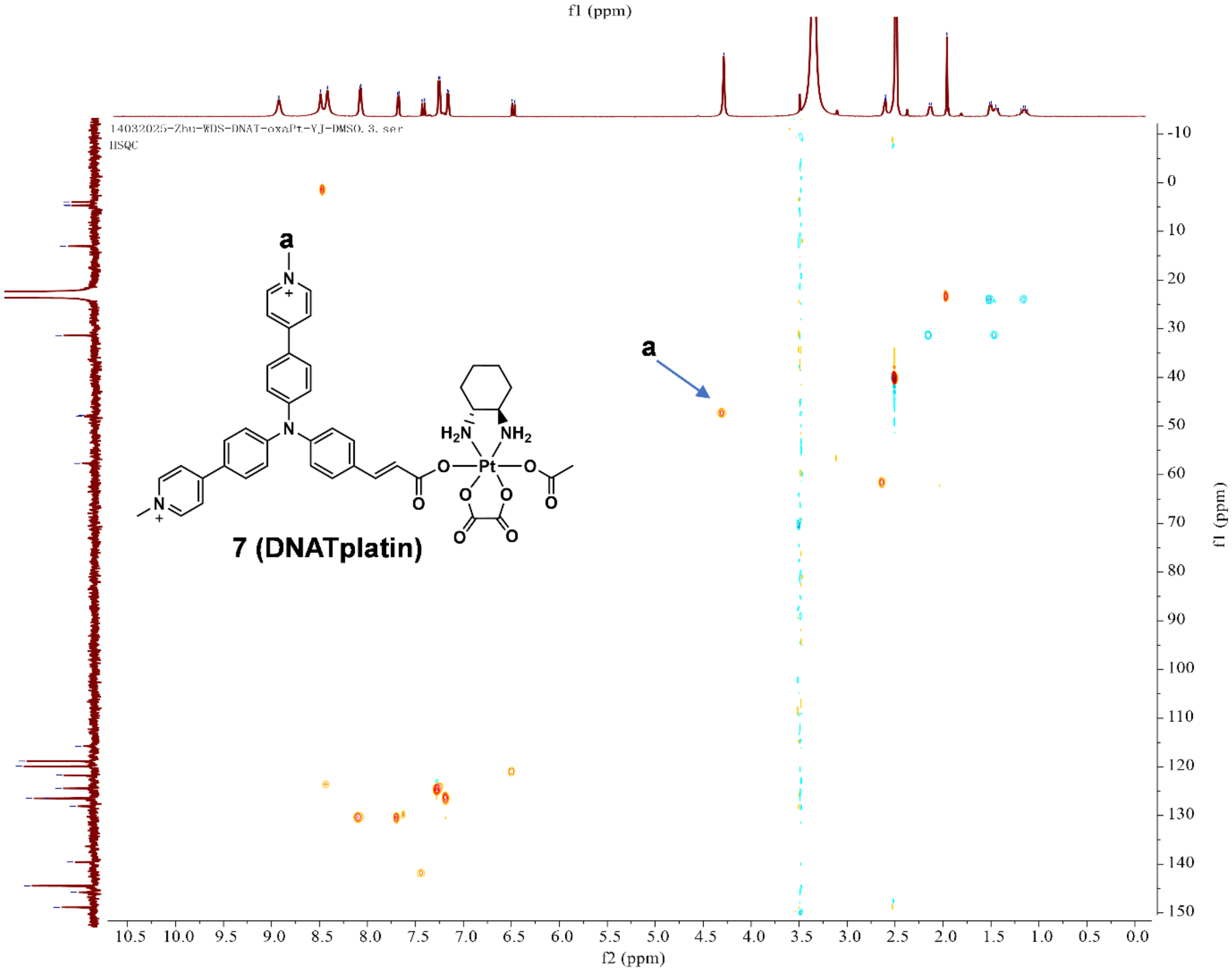

Figure S8. ^{195}Pt NMR and HSQC spectra of complex **7** (DNATplatin) in DMSO-d_6.

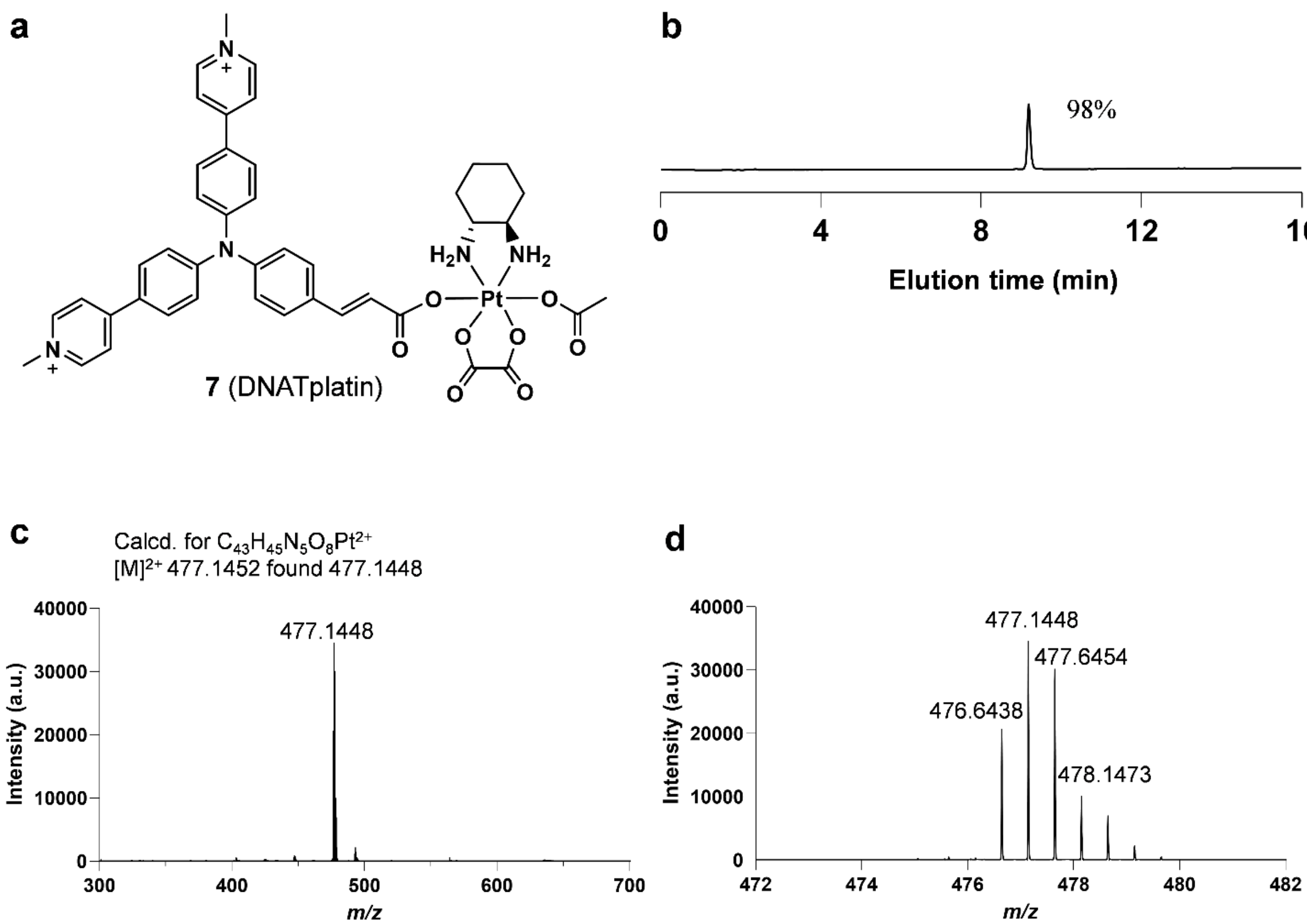

Figure S9. Chemical structure (a), HPLC (b), and ESI-HRMS (c-d) spectra of complex **7** (DNATplatin).

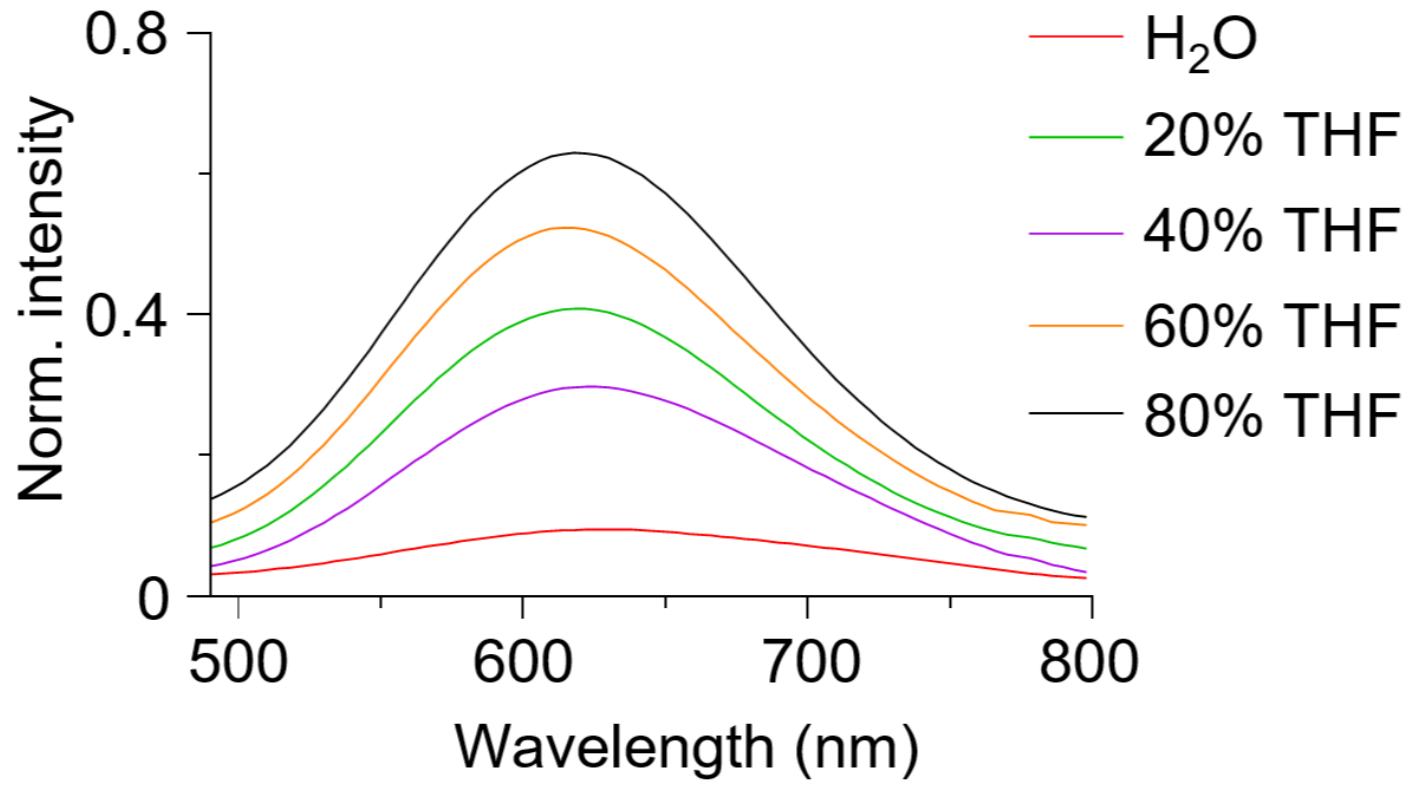

Figure S10. Normalized fluorescence emission spectra of DNATplatin in water and varying THF ratios, $\lambda_{ex} = 465$ nm.

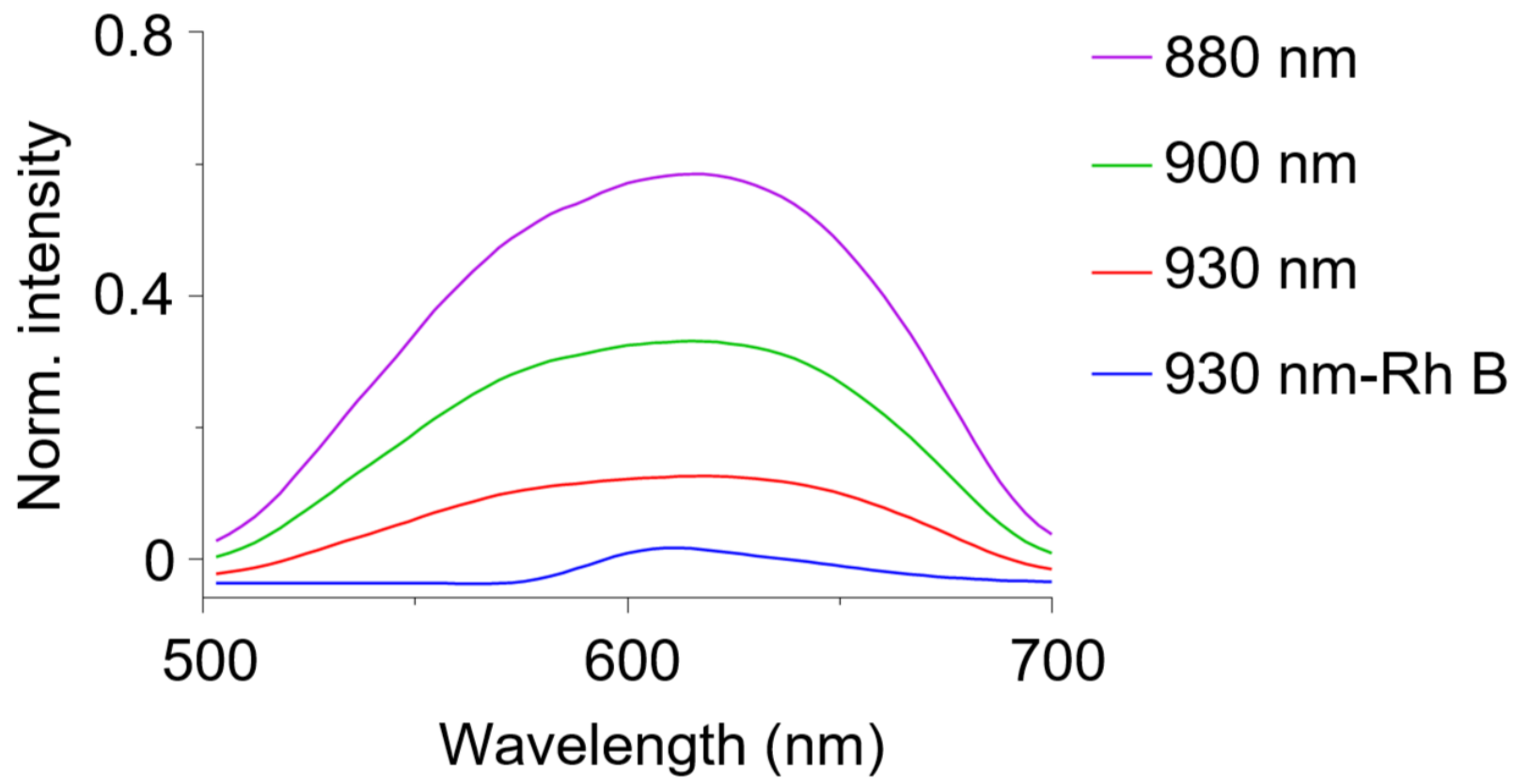

Figure S11. The normalized two-photon (880 nm, 900 nm, 930 nm) excited emission spectra of DNATplatin in 99% glycerol and rhodamine B (Rh-B) in MeOH. Two-photon excitation and emission detection were performed using a Leica confocal microscope (SP5).

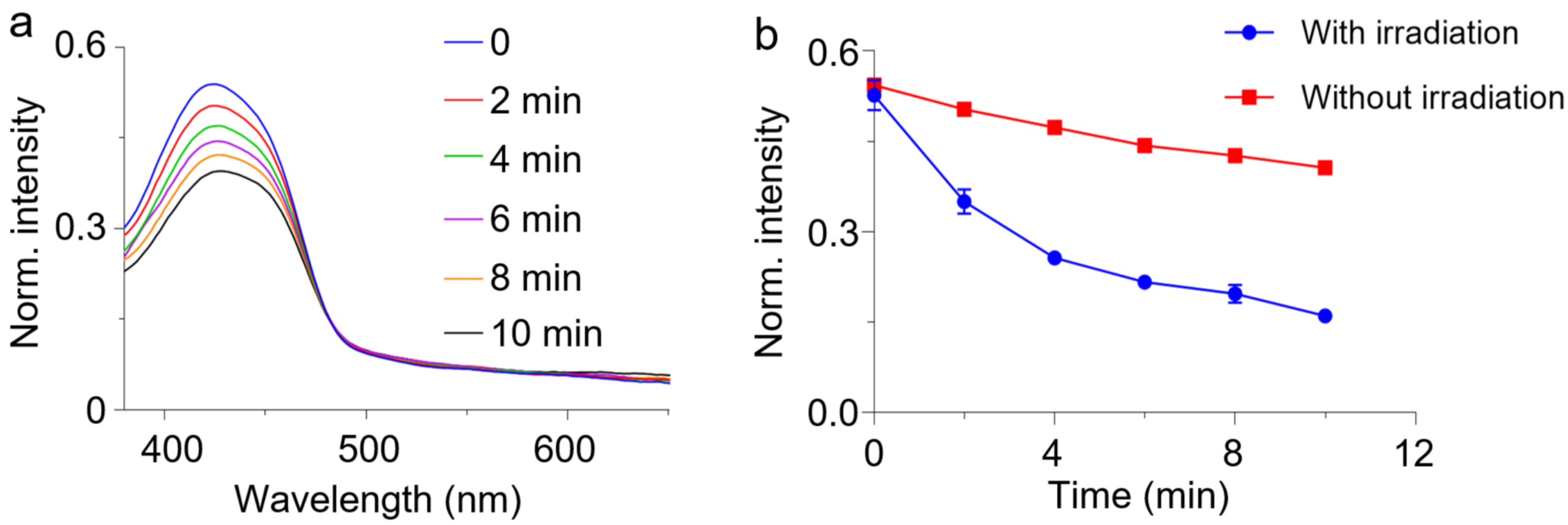

Figure S12. Absorption spectra change curves (a) and the variation curves of the maximum absorption value (b) for the mixture of DPBF and DNATplatin, with or without light irradiation.

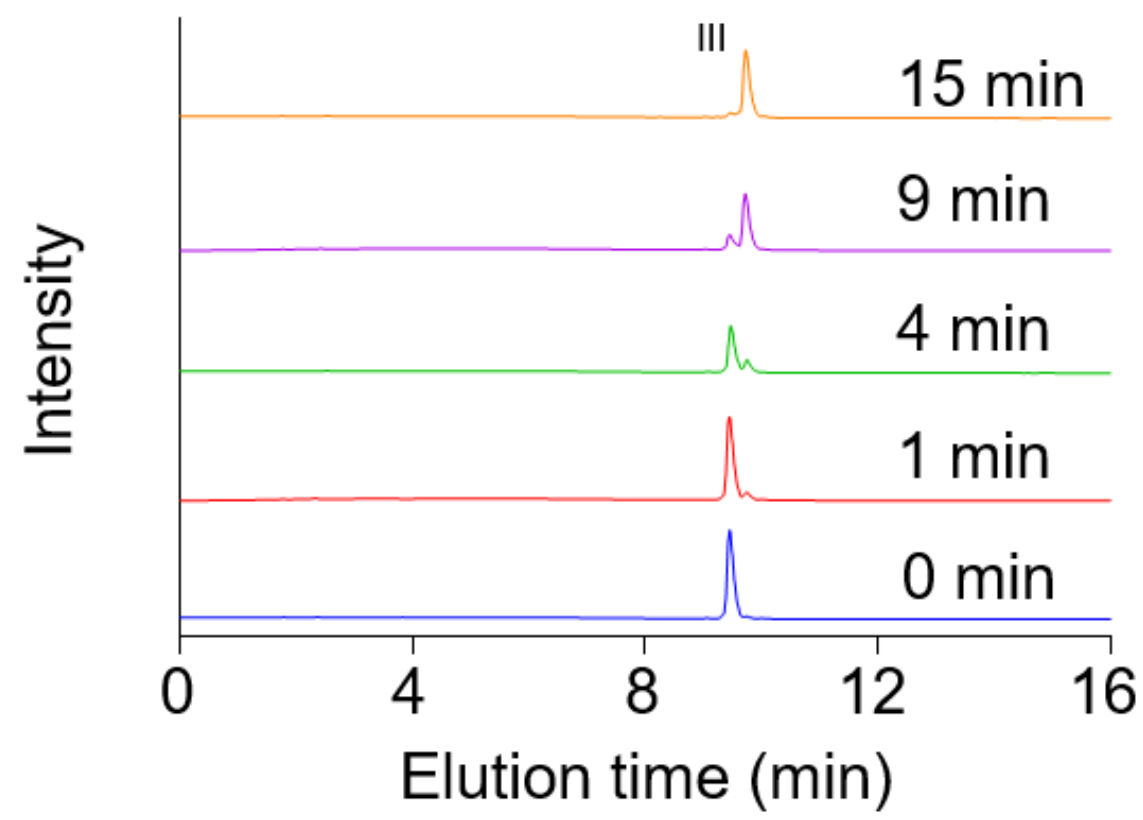

Figure S13. HPLC chromatograms (detection wavelength: 365 nm) of DNATplatin in PBS buffer containing 2 mM sodium ascorbate (pH 7.4) under light irradiation at 495 nm (1 mW cm^{-2}) for different durations.

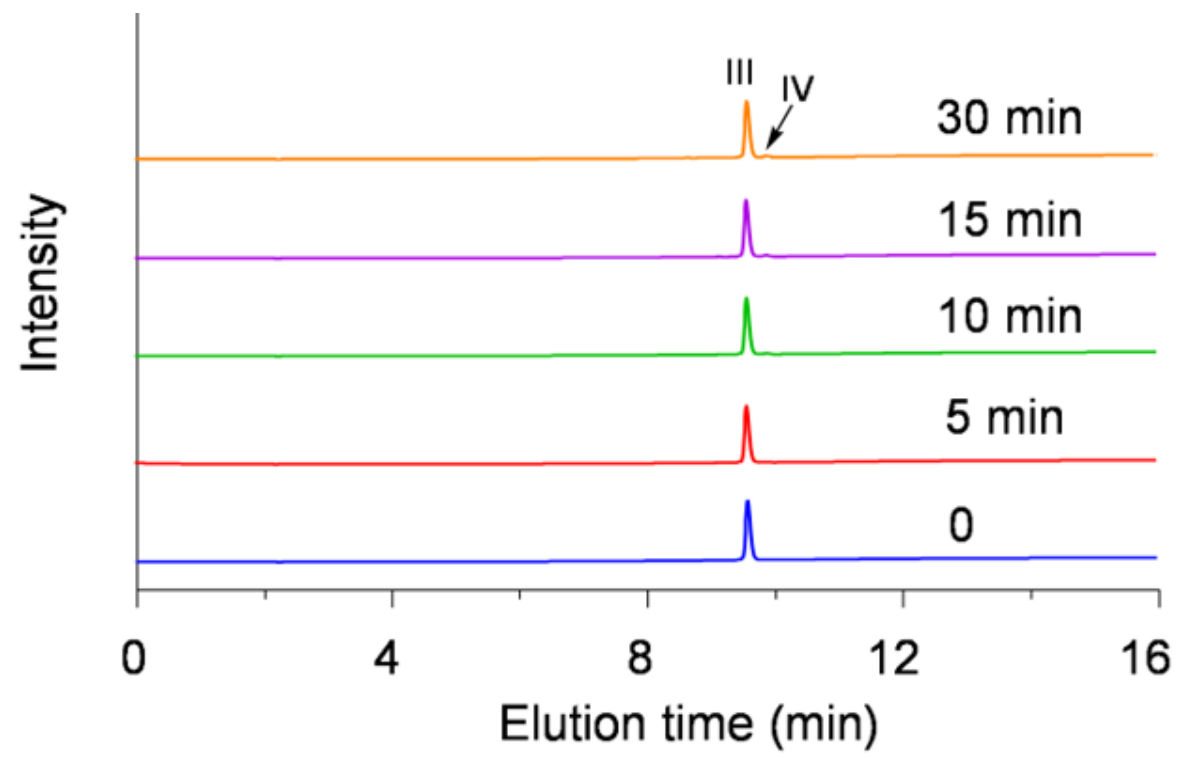

Figure S14. HPLC chromatographs of ligand in PBS buffer (pH 7.4) upon light irradiation for different periods of time, and the newly generated peaks were labeled.

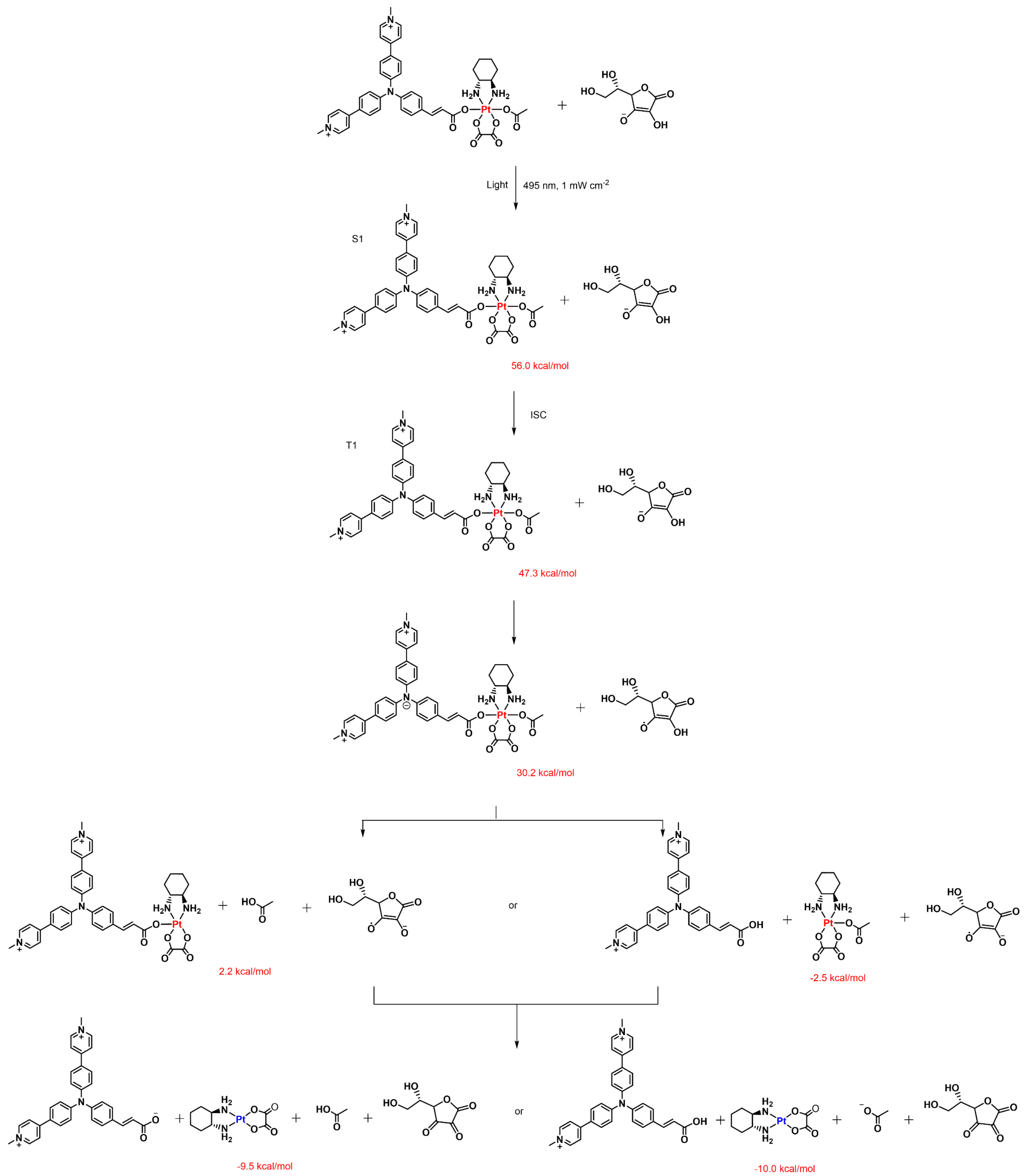

Figure S15. Density functional theory was employed to calculate the energy evolution during the photo-oxidation of ascorbate by DNATplatin. The corresponding energy is labeled above the structural formula.

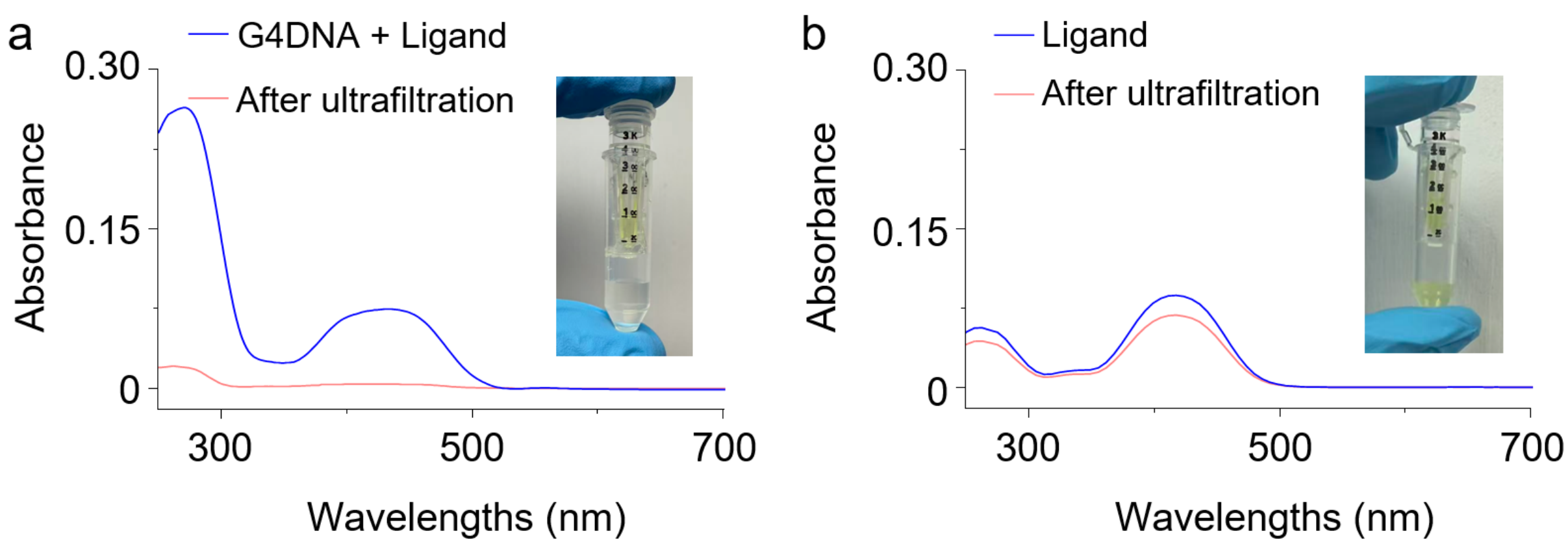

Figure S16. (**a**) Changes in the absorption spectra of G4 DNA with the binding solution containing ligand before and after ultrafiltration. (**b**) Changes in the absorption spectra of the ligand in PBS before and after ultrafiltration.

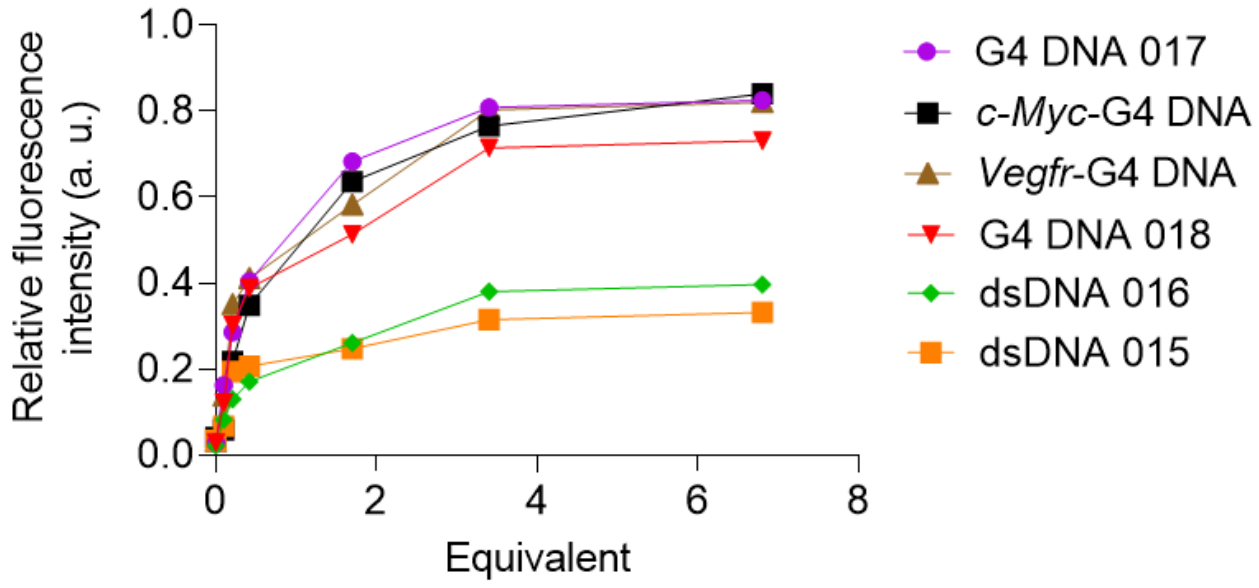

G4 DNA 017	5'-ATTGGGAAAGGGAAAGGGAAAGGGTT-3'
Myc161-G4 DNA	5'-TGGGGAGGGTGGGGAGGGTGGGGAA-3'
Vegfr-G4 DNA	5'-GGGTACCCGGGTGAGGTGCGGGGT-3'
G4 DNA 018	5'-ATAGGGATAGGGAAAGGGATAGGGTT-3'
dsDNA 016	5'-GGATCTGAATACCTCGGAAACATAGA-3'
dsDNA 015	5'-GGATCTGAATTCATCGCGATCCTAGA-3'

Figure S17. (a) Fluorescence intensity curves of DNATplatin (2.0 μM) upon titration with increasing equivalents of dsDNA or G4 DNA in a 10 mM Tris-HCl buffer (100 mM LiCl, pH 7.4), with λ_{ex} = 465 nm. (b) Corresponding gene sequences. G4 DNA 017 and 018 are randomly designed.

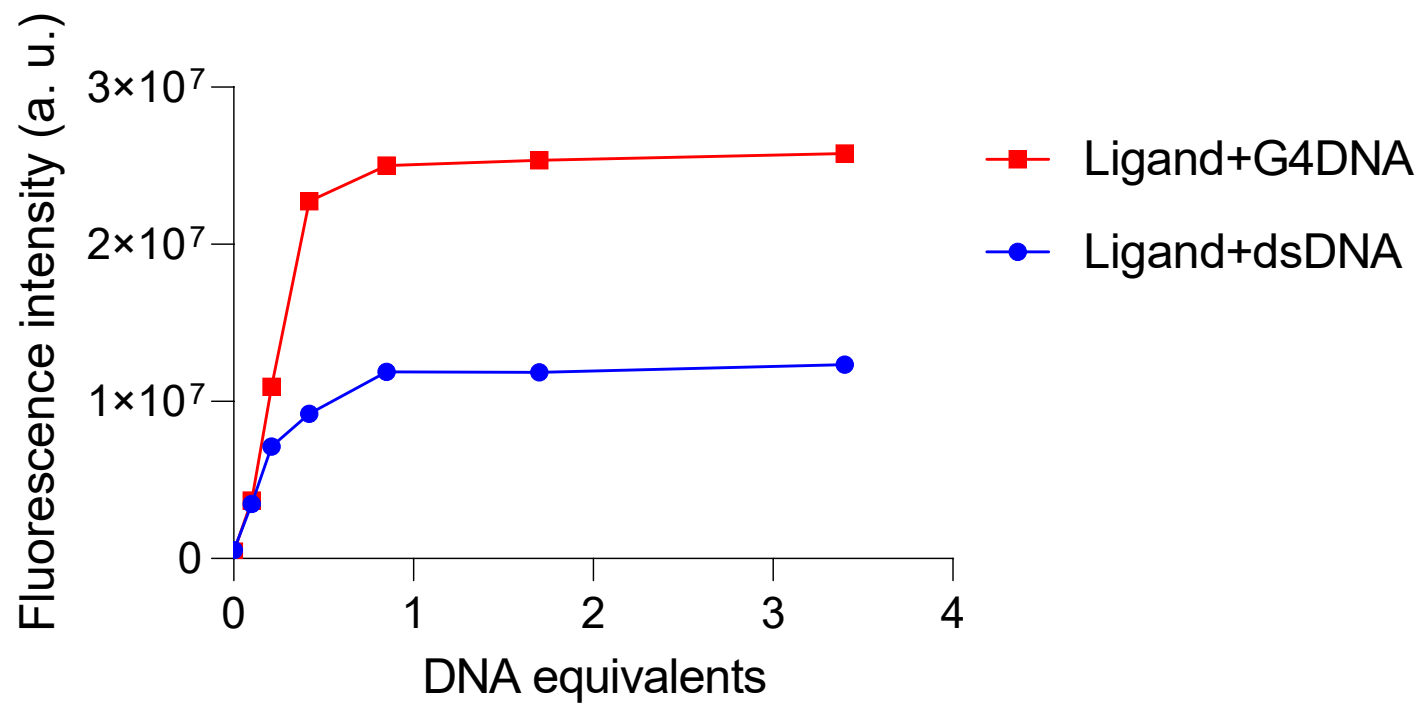

Figure S18. The fluorescence enhancement curves of ligand (2.0 μM) upon titration with increasing equivalents of G4 DNA (e) or dsDNA (f) in 10 mM Tris-HCl buffer containing 100 mM LiCl (pH 7.4, λ_{ex} = 465 nm). G4 DNA (5'-AAAGGGTTAGGGTTAGGGTTAGGGAA-3') and dsDNA (5'-CAATCGGATCGAATTCGATCCGATTG-3').

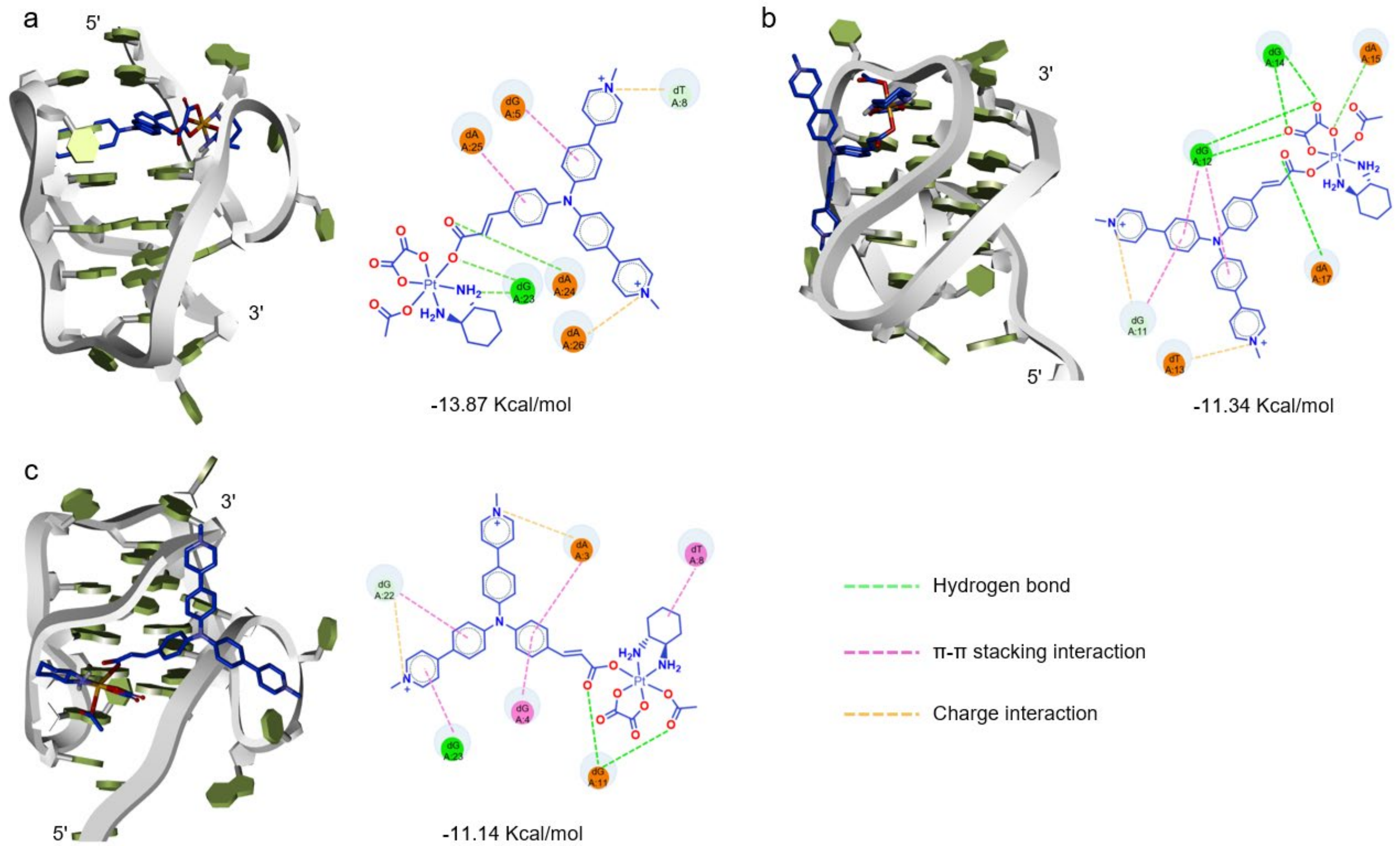

Figure S19. Molecular docking simulations of G4 DNA with DNATplatin (PDB: 6KFJ) at a 1:1 molar ratio. **(a–c)** Representative binding poses and corresponding detailed 2D interaction maps highlighting key residues, hydrogen bonds, and hydrophobic interactions. The detailed interactions show that DNATplatin binds to the deoxyadenosine (dA) and deoxyguanosine (dG) residues of the DNA receptor.

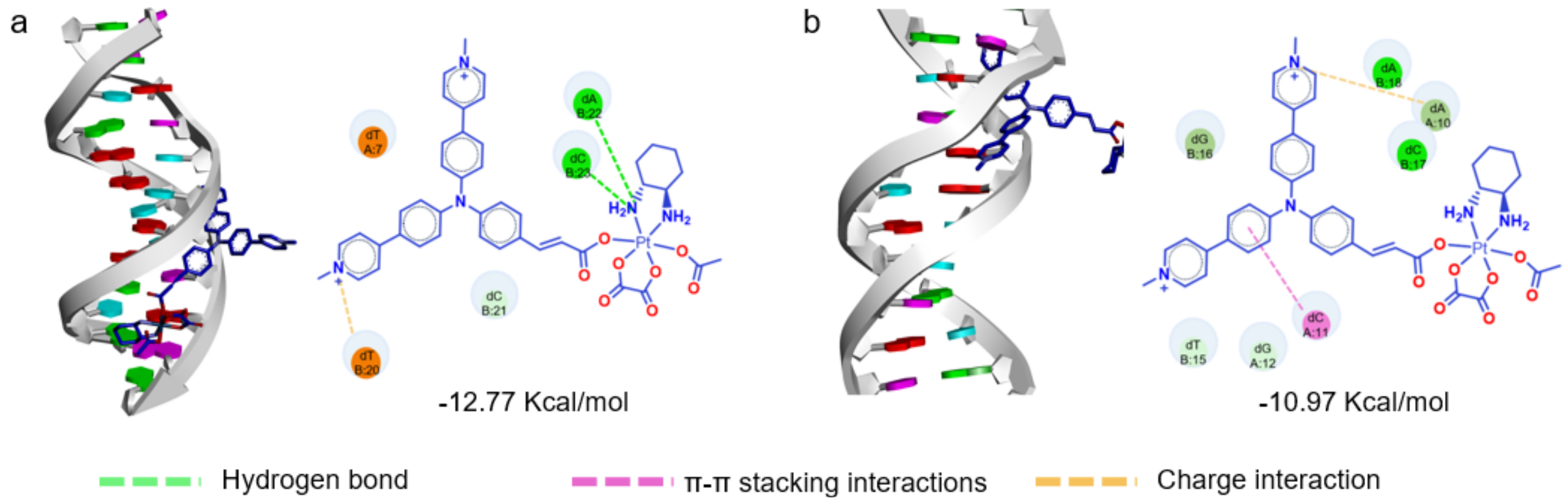

Figure S20. Molecular docking analysis of the interactions between dsDNA and DNATplatin at a 1:1 molar ratio. Representative binding poses and detailed 2D interaction maps highlighting key residues, hydrogen bonds, and hydrophobic interactions. DNATplatin binds electrostatically within the minor groove of dsDNA (PDB code: 5JU4).

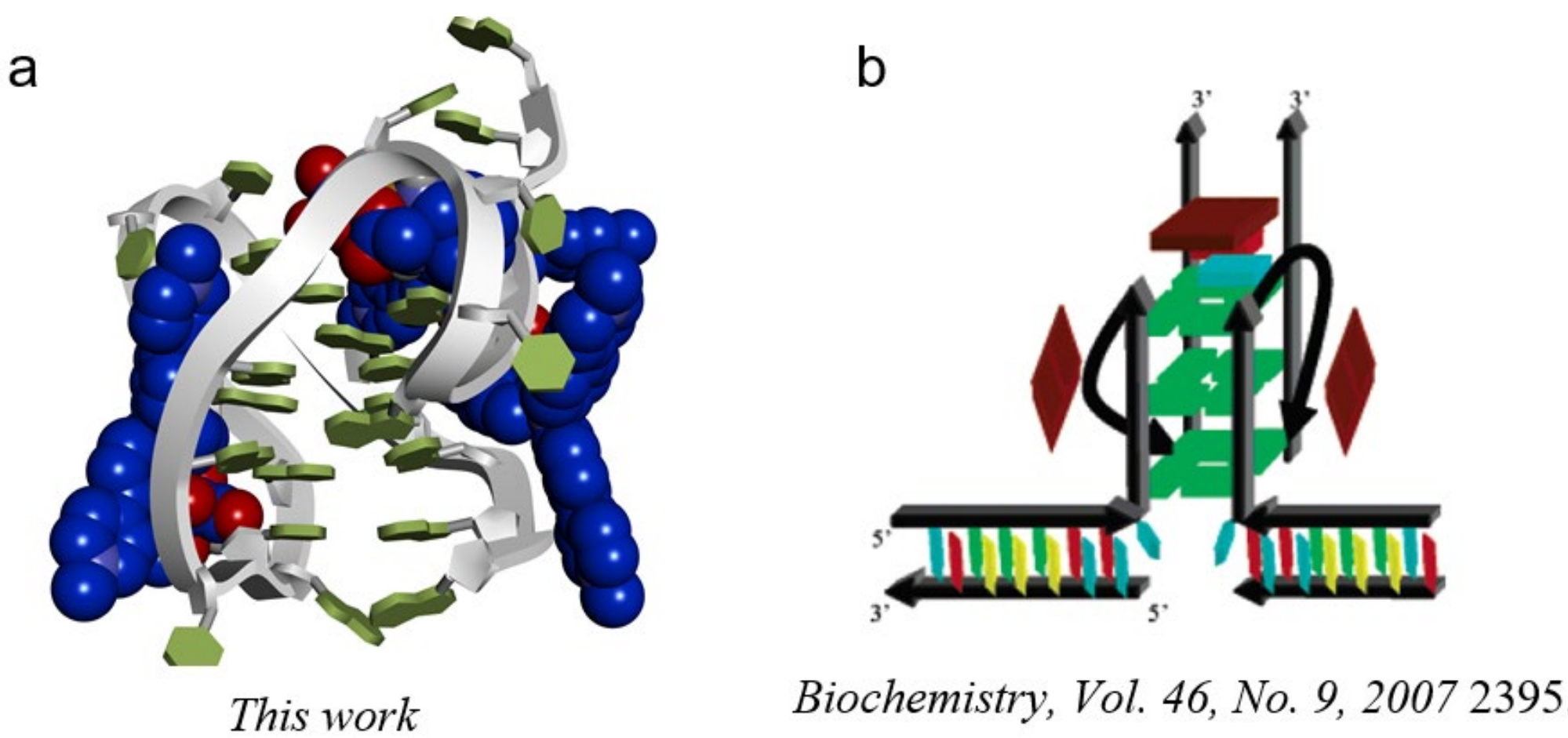

Figure S21. (**a**) Representative view of the docking model of G4 DNA binding with DNATplatin at a 1:3 molar ratio (PDB code: 6KFJ). (**b**) Schematic diagram of the binding between the binder and the G4 structure, as described in the literature. (Biochemistry, Vol. 46, No. 9, 2007, 2395).

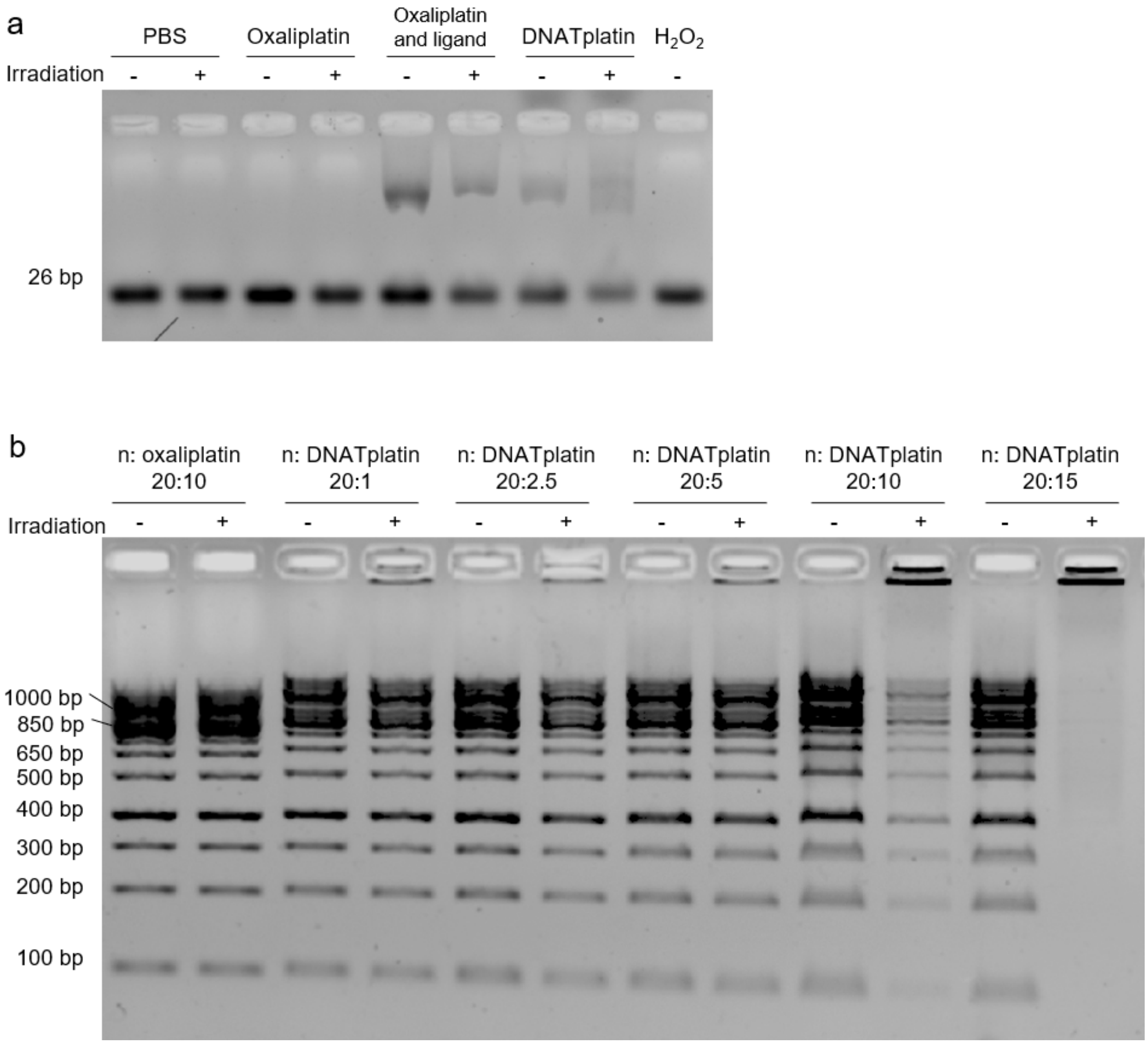

Figure S22. (**a**) Agarose gel image of dsDNA mixed with Pt-containing complexes with light or without light irradiation, obtained by agarose gel electrophoresis. (**b**) Agarose gel image of DNA ladder mixed with DNATplatin at different nucleotides (n) to DNATplatin ratios, with light or without light irradiation, obtained by agarose gel electrophoresis.

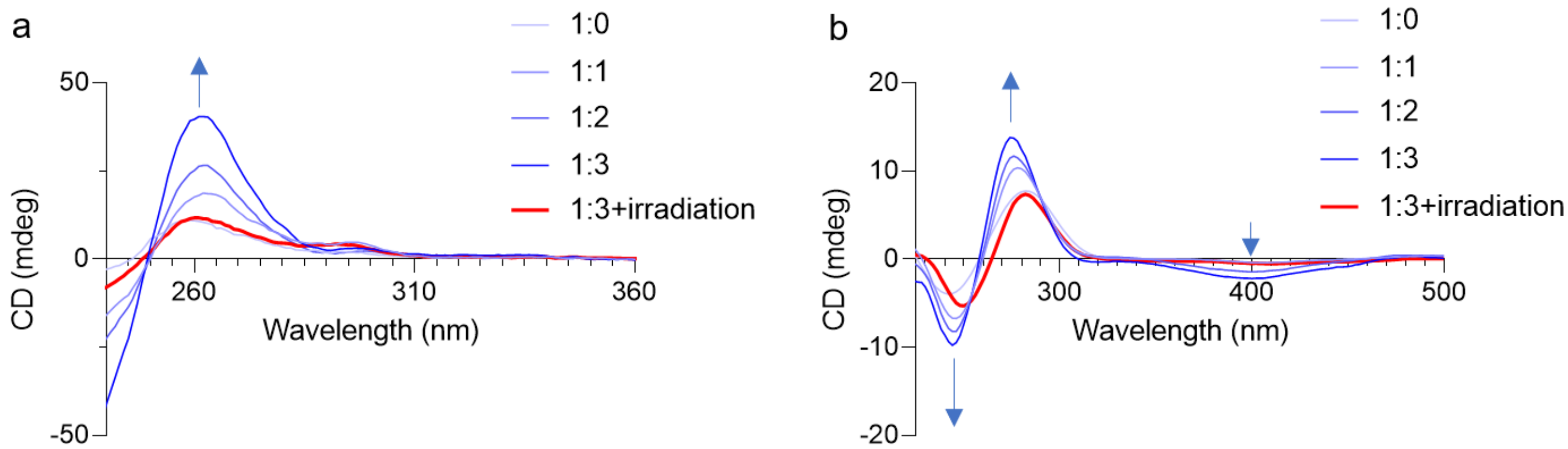

Figure S23. CD spectra of G4 DNA (a) (5'-AAAGGGTTAGGGTTAGGGTTAGGGAA-3') and dsDNA (b) (5'-CAATCGGATCGAATTCGATCCGATTG-3') in 10 mM Tris-HCl buffer (100 mM LiCl, pH 7.4) upon titration with ligand at molar ratios (dsDNA: ligand or DNATplatin) ranging from 1:1 to 1:3. The ligand + irradiation or the DNATplatin+irradiation group (red line) was measured at the molar ratio of 1:3.

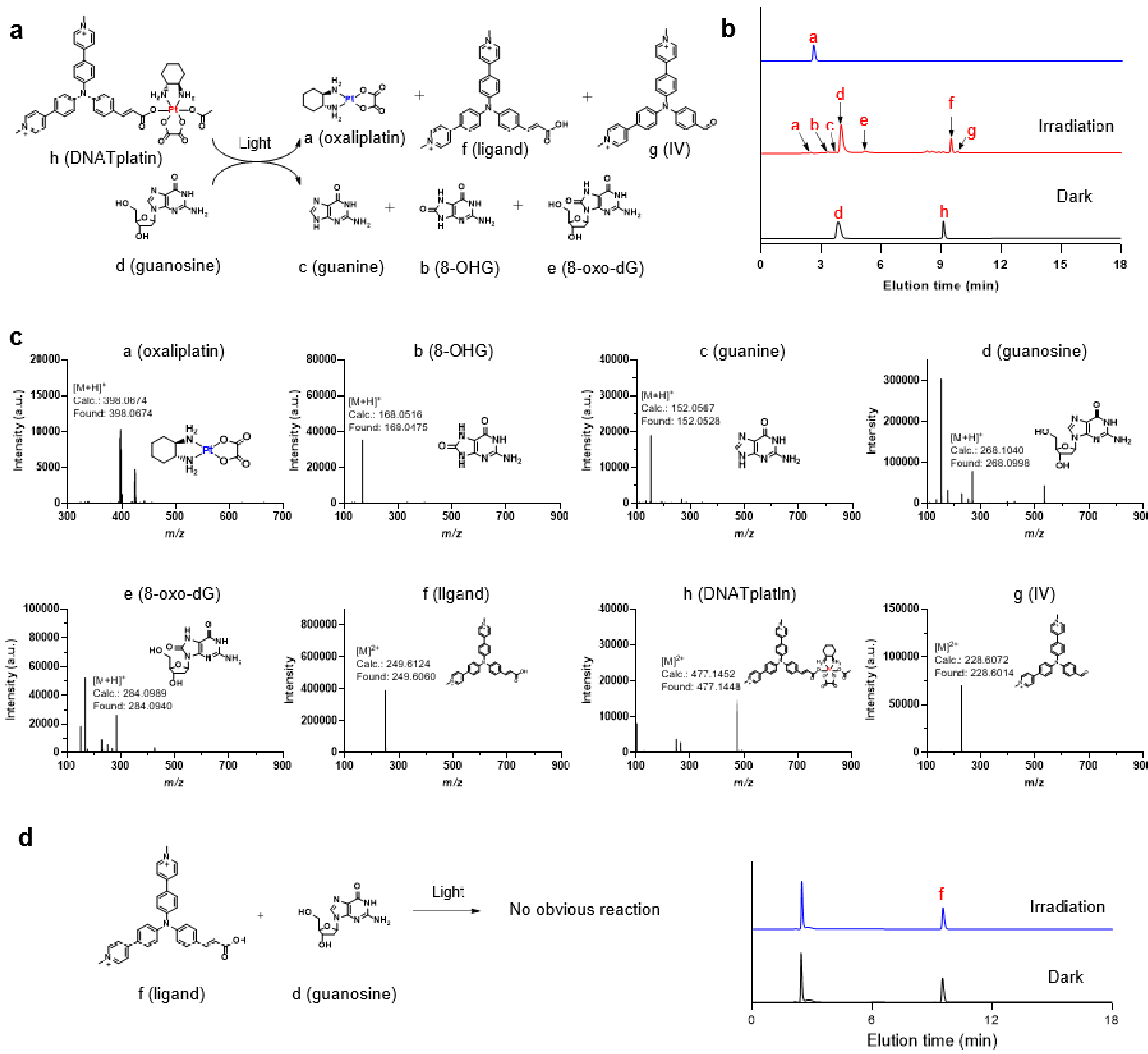

Figure S24. DNATplatin oxidizes guanosine under light irradiation. (**a**) The reaction of DNATplatin to oxidize guanosine under light irradiation. (**b**) The HPLC chromatograph of the guanosine (10 μM) and DNATplatin (2 μM) mixture in H_2O before and after light irradiation for 30 min. Eluent program: 0.01 min 95% A, 5% B; 7.00 min 51% A, 49% B; 13.00 min 45% A, 55% B; 14.00 min 95% A, 5% B; 18.00 min 95% A, 5% B. Solution A is H_2O + 0.1% formic acid. Solution B is acetonitrile + 0.1% formic acid. (**c**) The LC-MS analysis results of a (oxaliplatin), b (8-OHG), c (guanine), d (guanosine), e (8-oxa-dG), f (ligand), h (DNATplatin), g (IV) from **b**. (**d**) The ligand and guanosine showed no obvious reaction under light irradiation and the HPLC chromatograph of the guanosine (10 μM) and ligand (2 μM) mixture in H_2O before and after light irradiation. In **d**, the advanced retention time of guanosine stems from the localized protonation effect induced by pre-mixing with the carboxyl-bearing ligand.

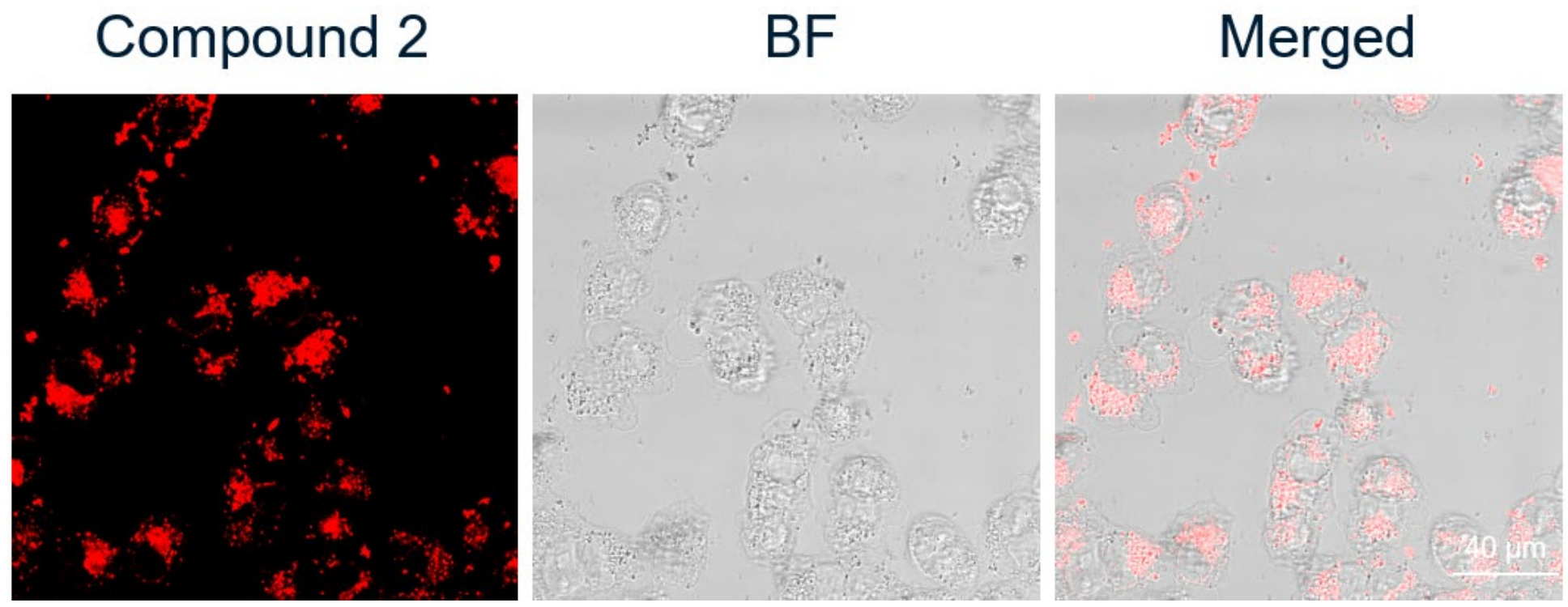

Figure S25. Confocal images for the accumulation of 10 μM compound **2** in 4T1 cells.

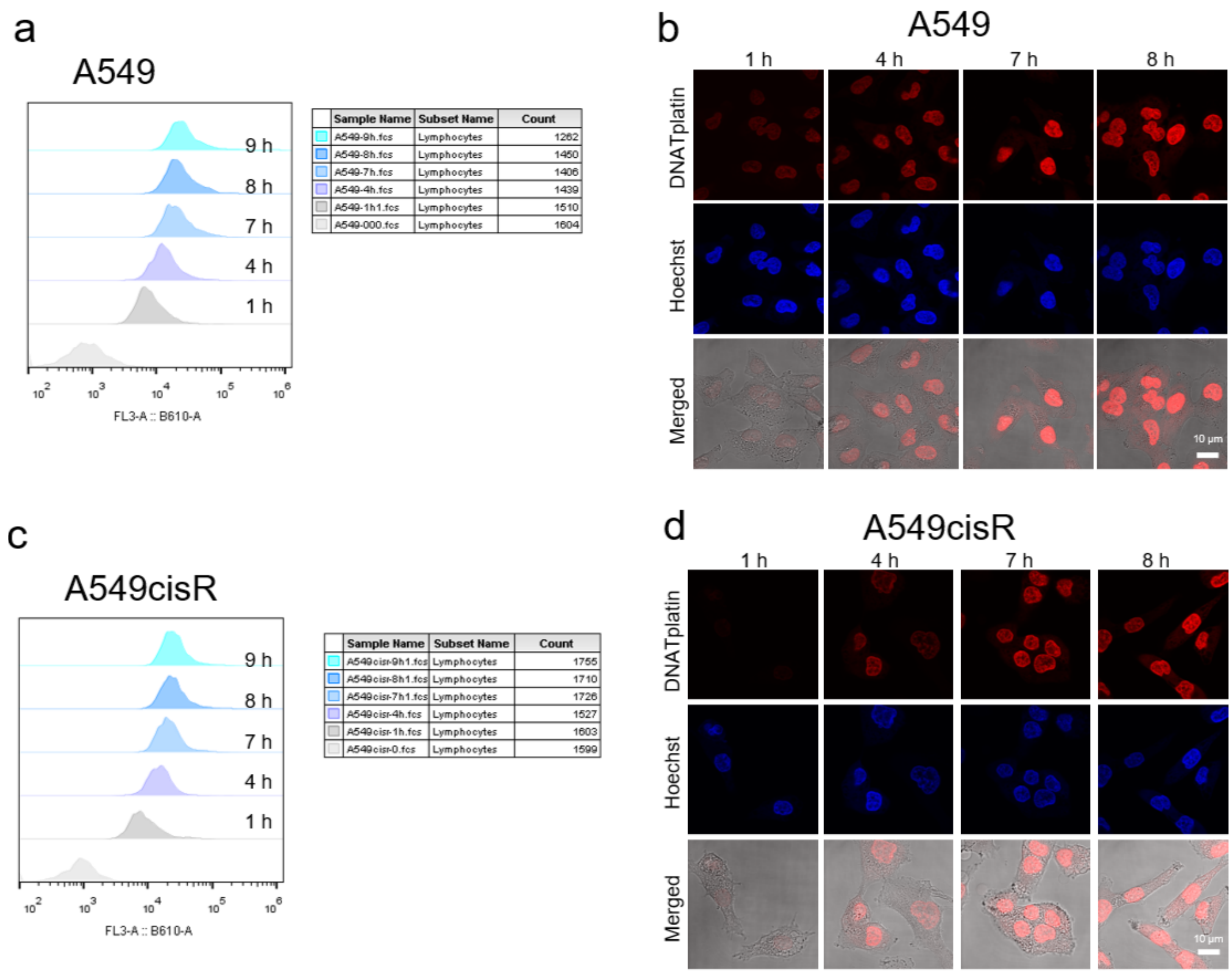

Sample Name	Subset Name	Count
A549-9h.fcs	Lymphocytes	1262
A549-8h.fcs	Lymphocytes	1450
A549-7h.fcs	Lymphocytes	1406
A549-4h.fcs	Lymphocytes	1439
A549-1h1.fcs	Lymphocytes	1510
A549-000.fcs	Lymphocytes	1604

Sample Name	Subset Name	Count
A549cisr-9h1.fcs	Lymphocytes	1755
A549cisr-8h1.fcs	Lymphocytes	1710
A549cisr-7h1.fcs	Lymphocytes	1726
A549cisr-4h.fcs	Lymphocytes	1527
A549cisr-1h.fcs	Lymphocytes	1603
A549cisr-0.fcs	Lymphocytes	1599

Figure S26. Time-dependent uptake of 10 μM DNATplatin by A549 (a, b) and A549*cis*R (c, d) cells, assessed by flow cytometry and confocal imaging. Confocal images were taken 8 h post-incubation, showing predominant nuclear localization of the drug (red) in both cell lines.

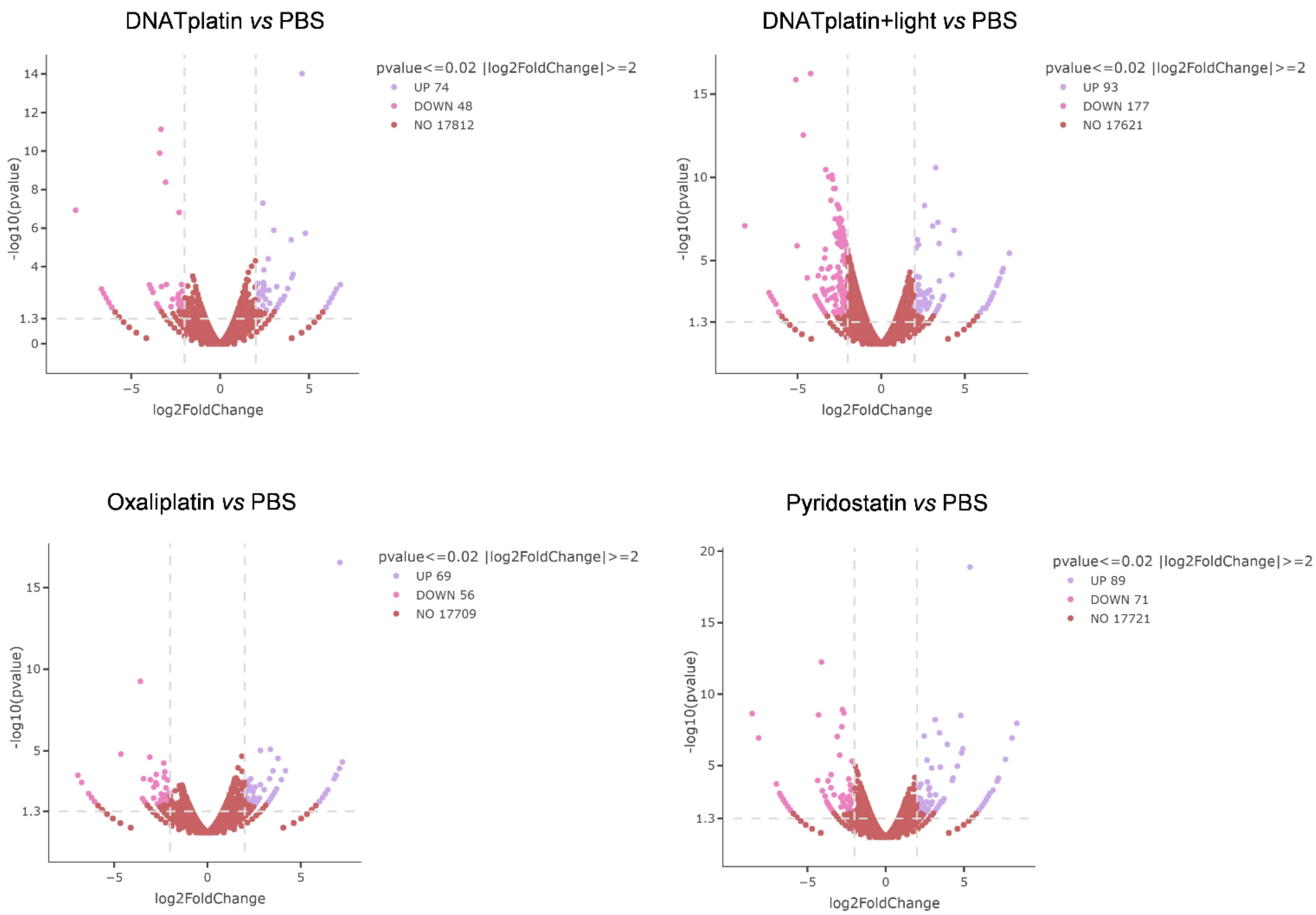

Figure S27. Counts of differentially expressed genes (DEGs) in 4T1 cells treated with 2 μM DNATplatin, with or without light irradiation, as well as 2 μM oxaliplatin and pyridostatin.

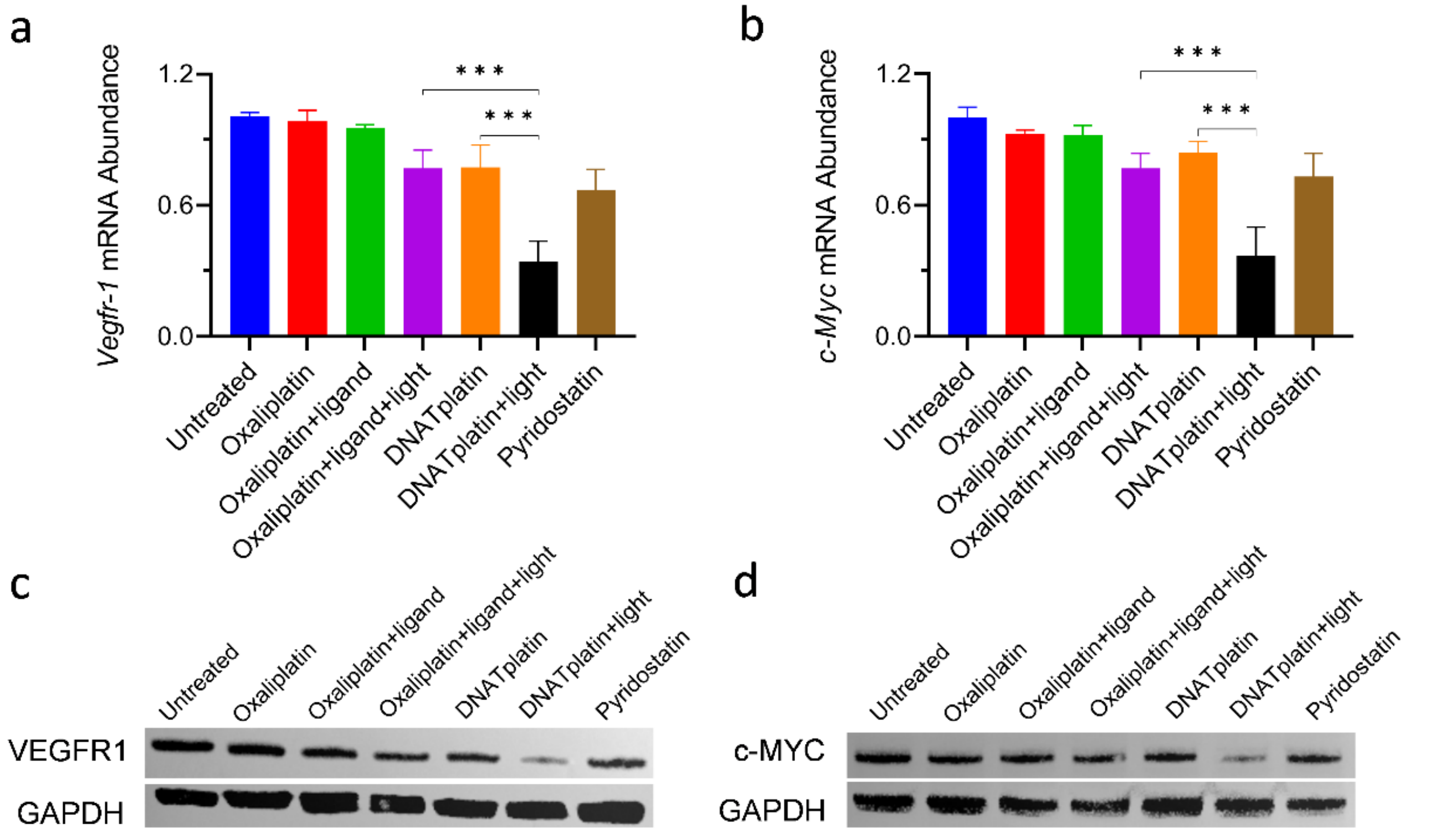

Figure S28. The mRNA expression levels of *Vegfr-1* (a) and *c-Myc* (b) were measured by RT-qPCR alongside the corresponding western blot analysis of VEGFR1 (c) and c-MYC (d) proteins in 4T1 cells treated with oxaliplatin+ligand (2 μM) with or without light irradiation, DNATplatin (2 μM) with or without light irradiation, as well as 2 μM oxaliplatin and pyridostatin. GAPDH was used as the internal control.

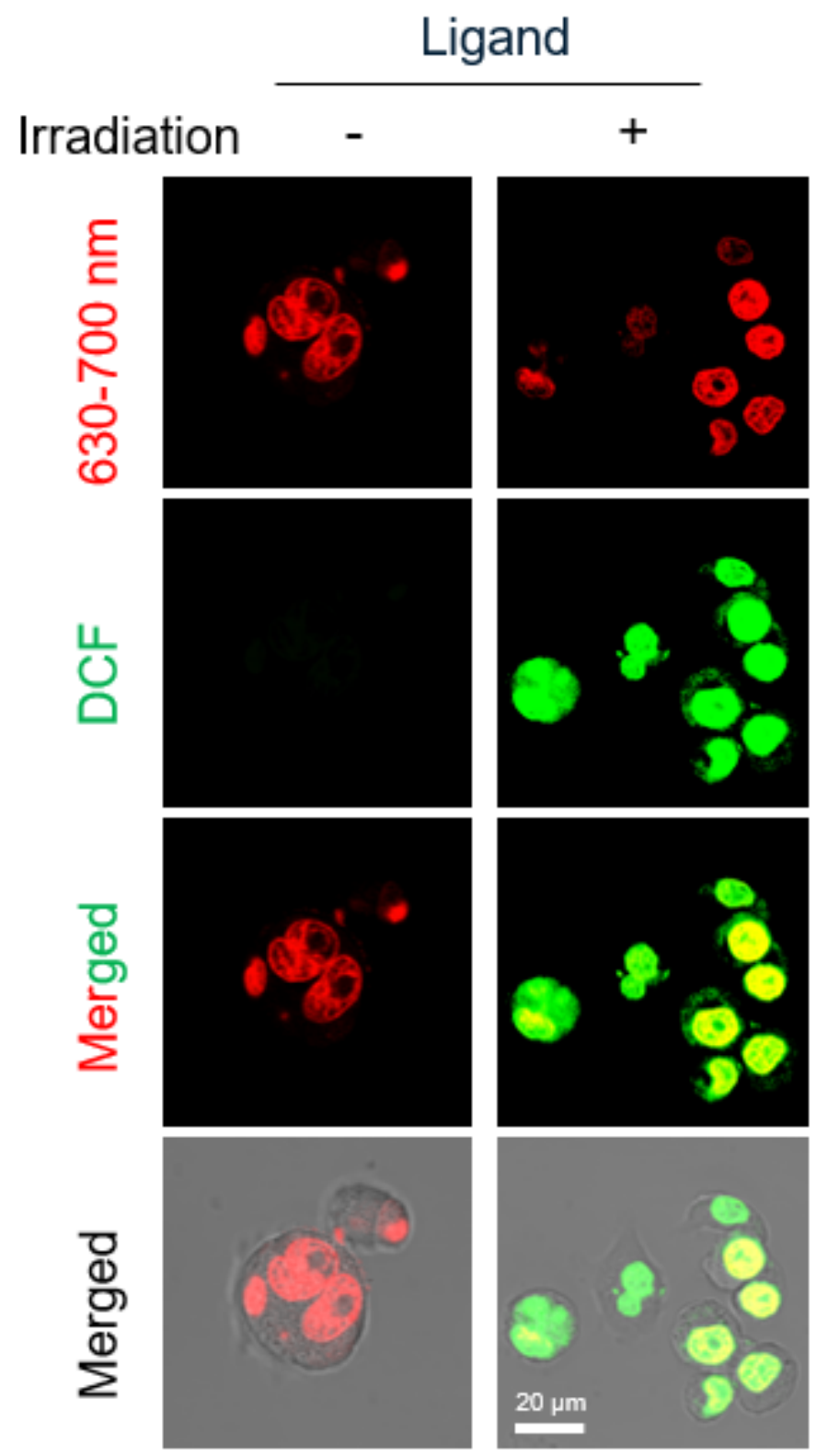

Figure S29. Fluorescent confocal images of H_2DCFDA-stained 4T1 cells treated with the ligand (10 μM) for 8 h followed by green light irradiation (1 mW m^{-2}) for 2 min (λ_{ex} = 488 nm, λ_{em}= 517–527 nm).

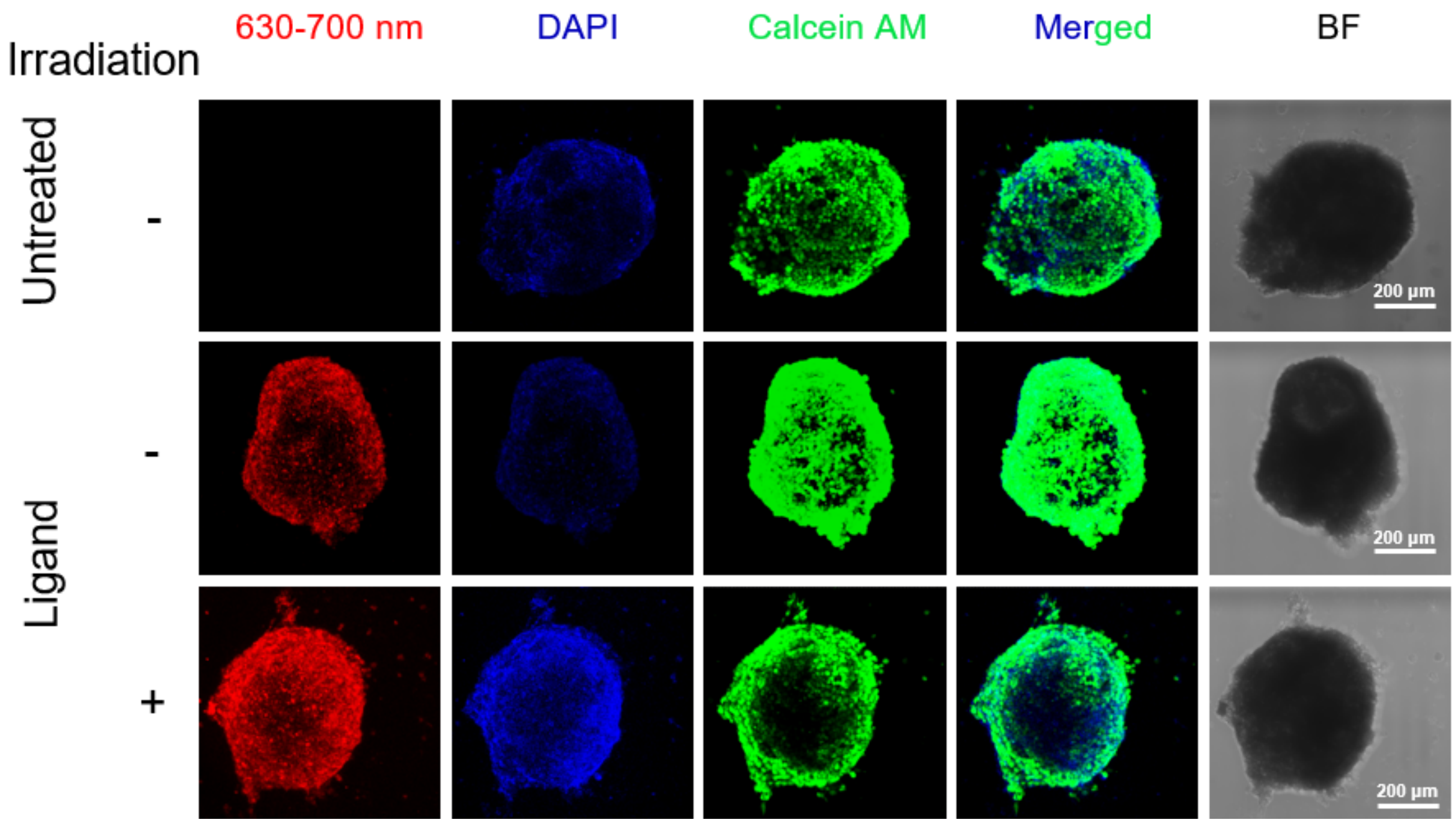

Figure S30. The live/dead cell staining of 4T1 tumor spheroids under different treatments was assessed, with spheroids treated with 10 μM ligand and PBS for 6 h, followed by fresh medium replacement, irradiation with or without light for 30 min, and subsequent culturing for 24 h. Live/dead cells were stained with Calcein AM/DAPI, and spheroids were observed using confocal microscopy.

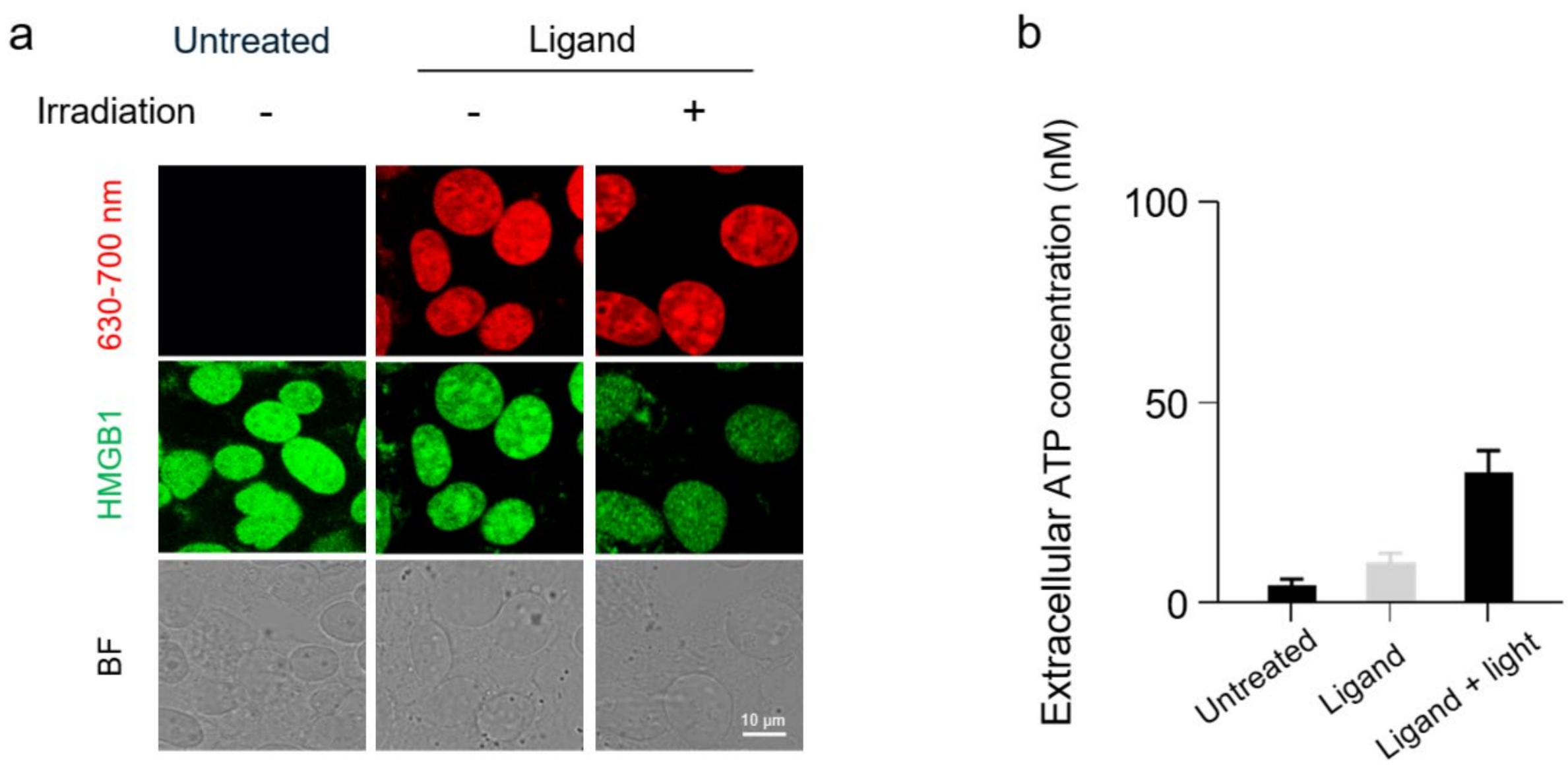

Figure S31. (**a**) Immunofluorescence confocal images of HMGB1 translocation from the nucleus to the cytoplasm and (**b**) ATP efflux in 4T1 cells treated with ligand (2 μM) followed by green light irradiation (1 mW cm^{-2}) for 30 min (λ_{ex} = 488 nm, green channel λ_{em}= 517-527 nm and red channel λ_{em} = 630-700 nm).

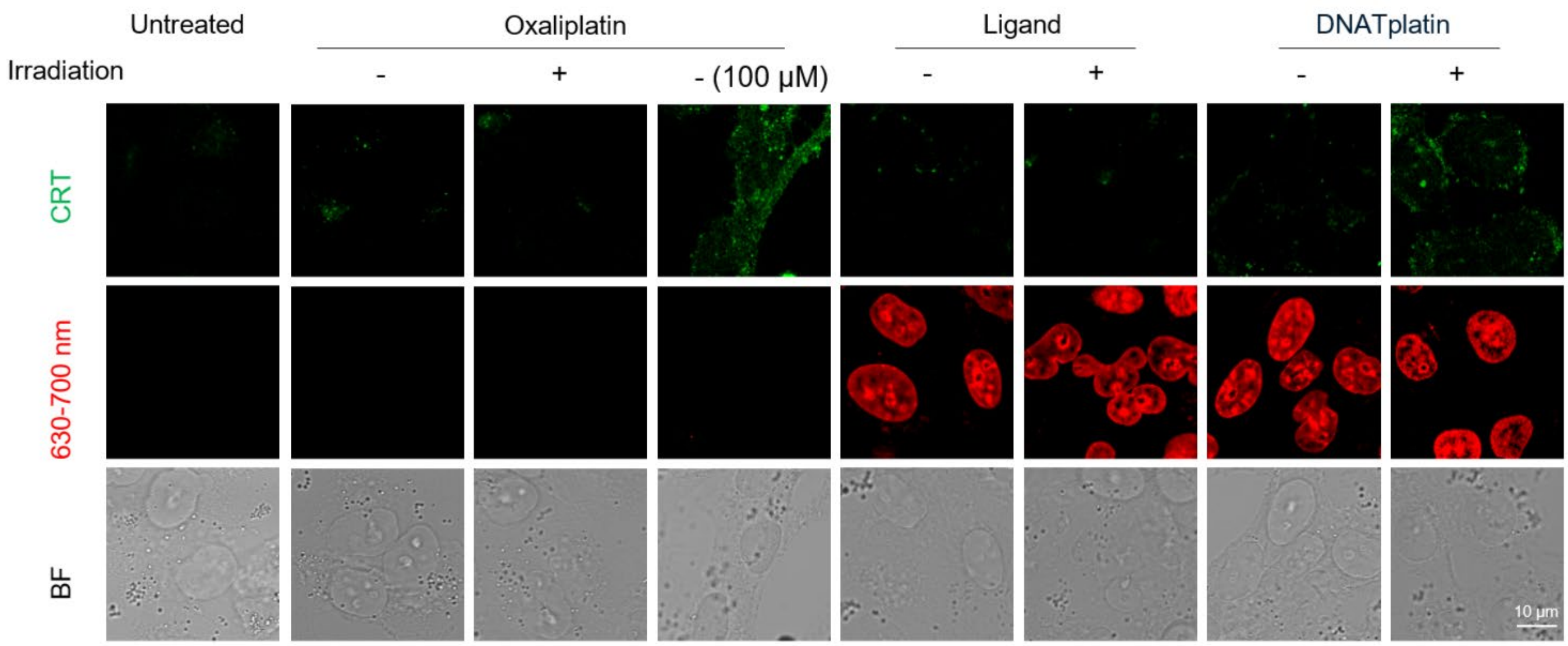

Figure S32. Immunofluorescence confocal images of CRT exposure in 4T1 cells treated with oxaliplatin (2 and 100 μM), ligand (2 μM), and DNATplatin (2 μM) followed by light irradiation (1 mW cm^{-2}) for 30 min (λ_{ex} = 488 nm, green channel λ_{em}= 517–527 nm, and red channel λ_{em} = 630–700 nm).

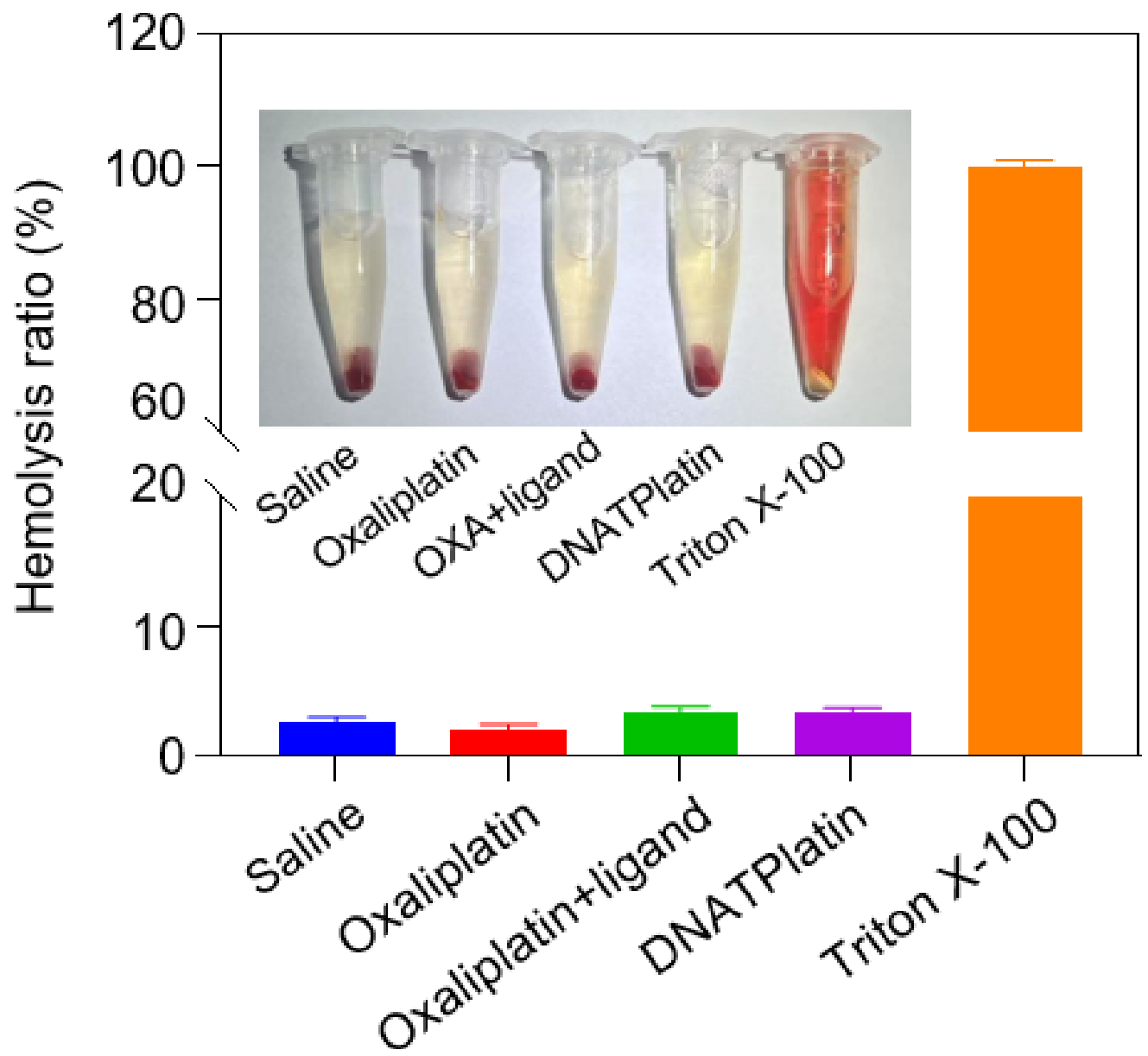

Figure S33. *In vitro* hemolysis assay of all Pt-containing formulations, and physical photos after testing.

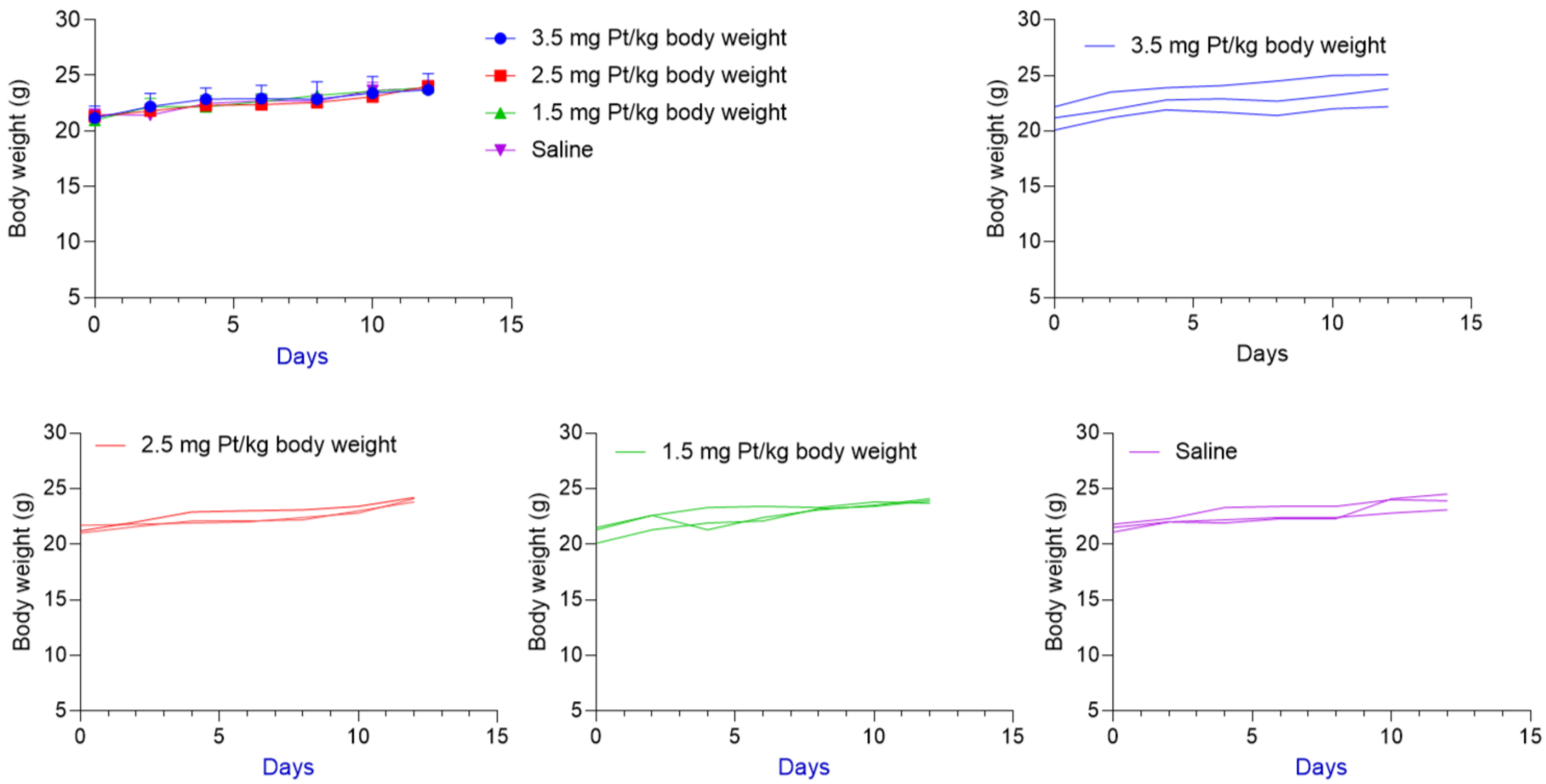

Figure S34. Body weight changes in mice after a single intravenous injection of DNATplatin. The doses administered were 3.5, 2.5, 1.5 mg Pt kg^{-1} body weight. (n = 3)

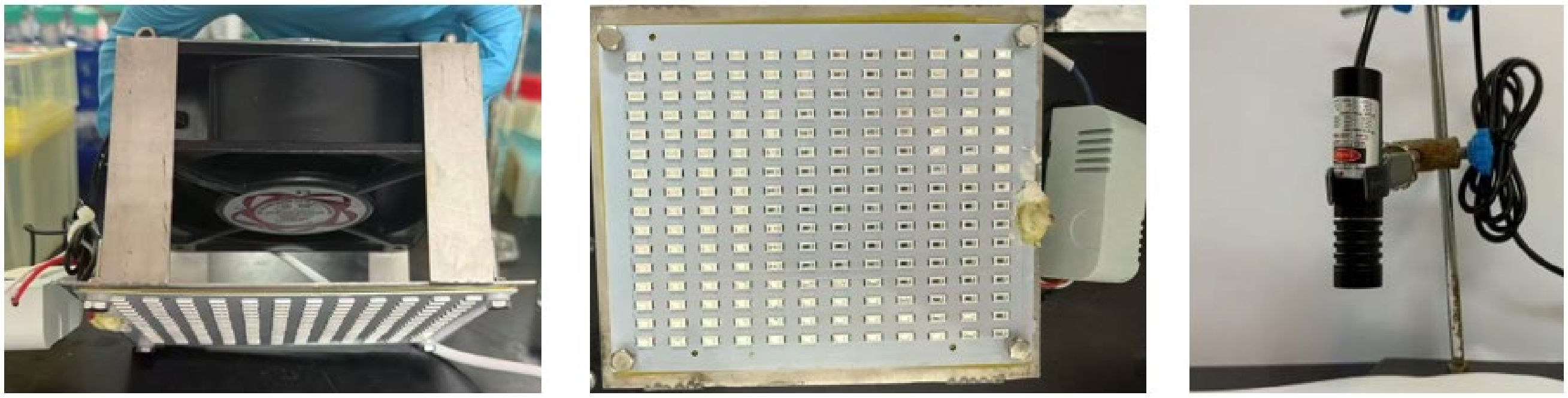

Figure S35. Physical picture of the light.

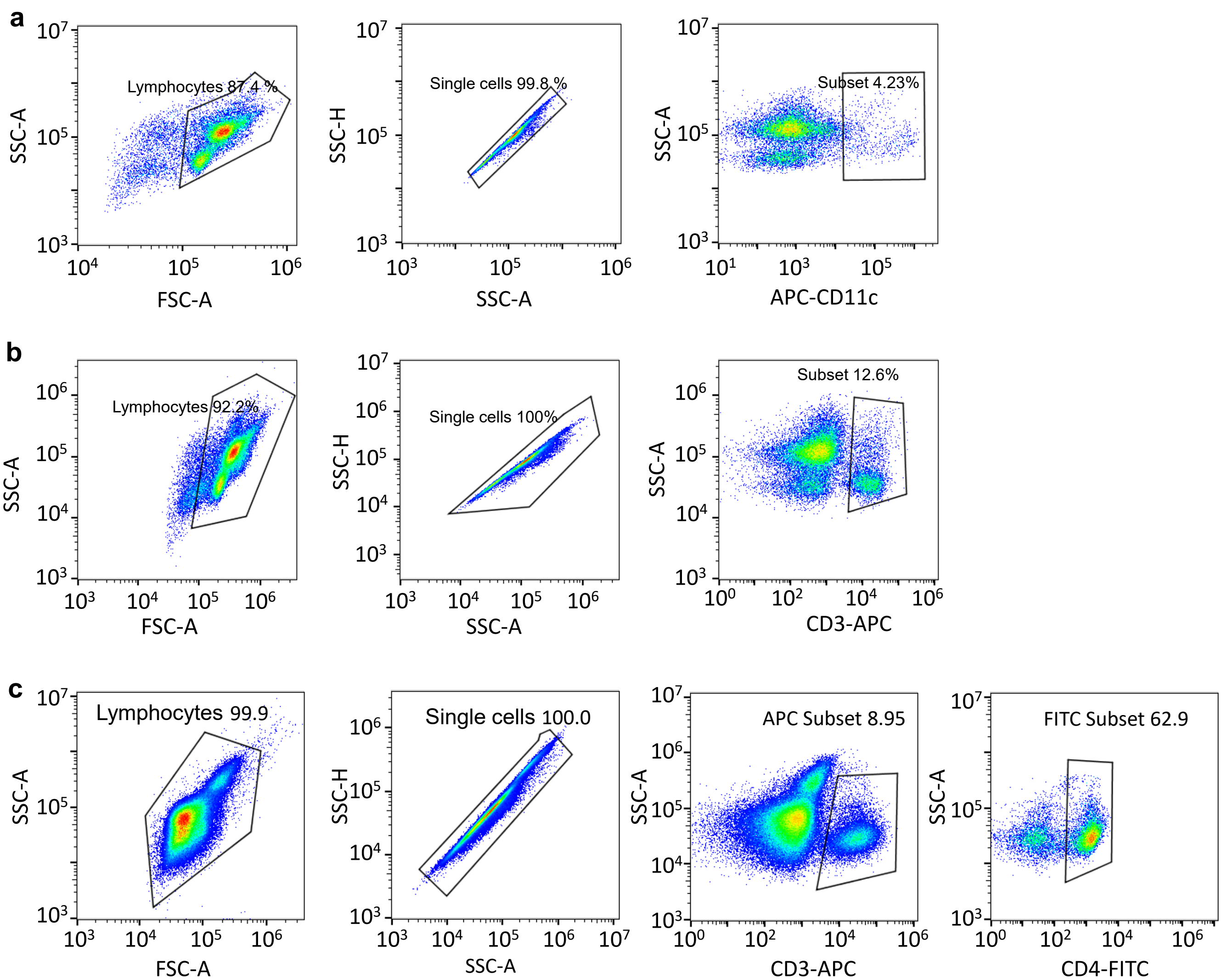

Figure S36. Gating control of flow cytometry immune cells.

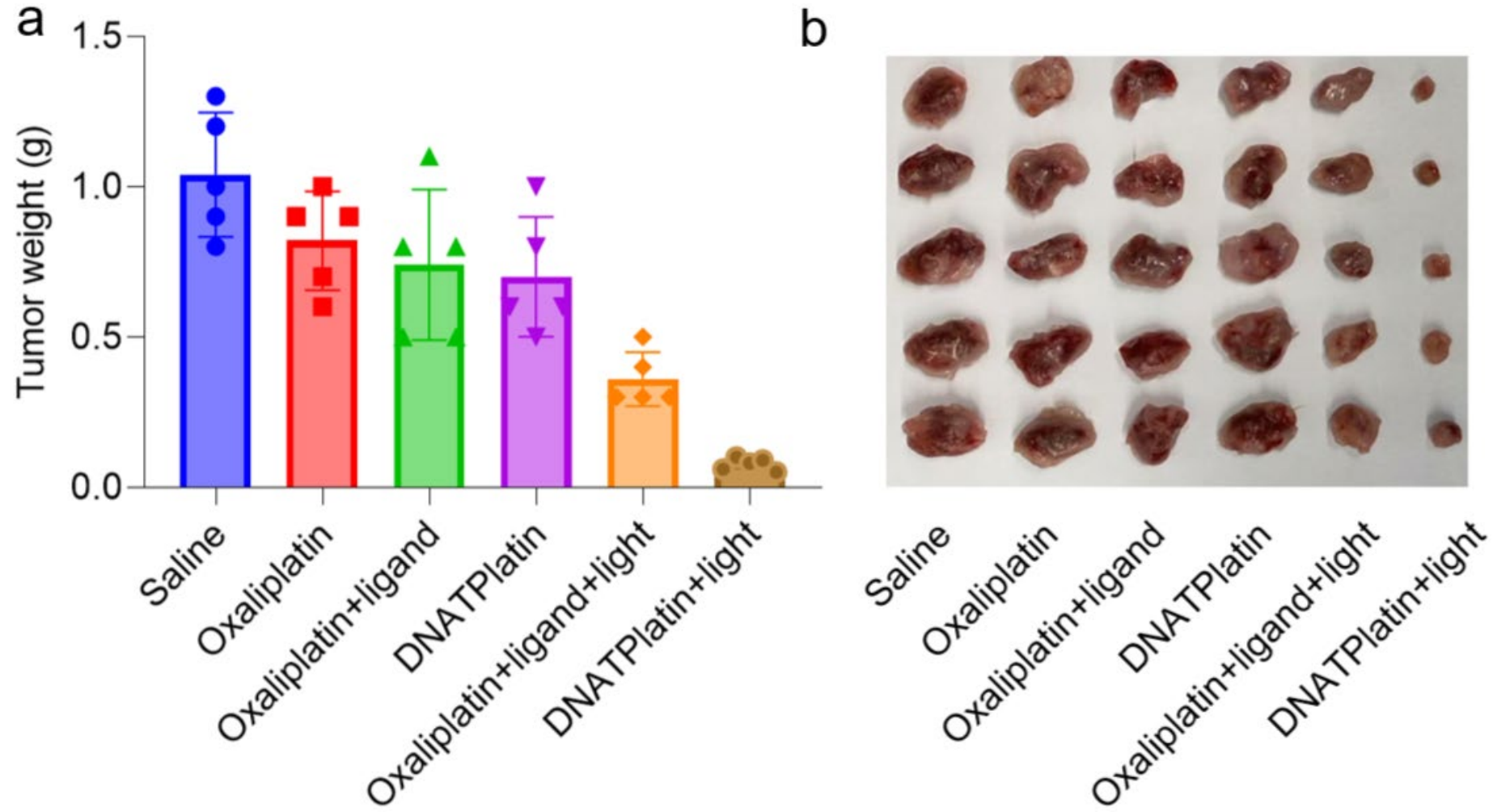

Figure S37. (**a**) Tumor weights and (**b**) tumor image of all mice were measured after three treatments on day 12.

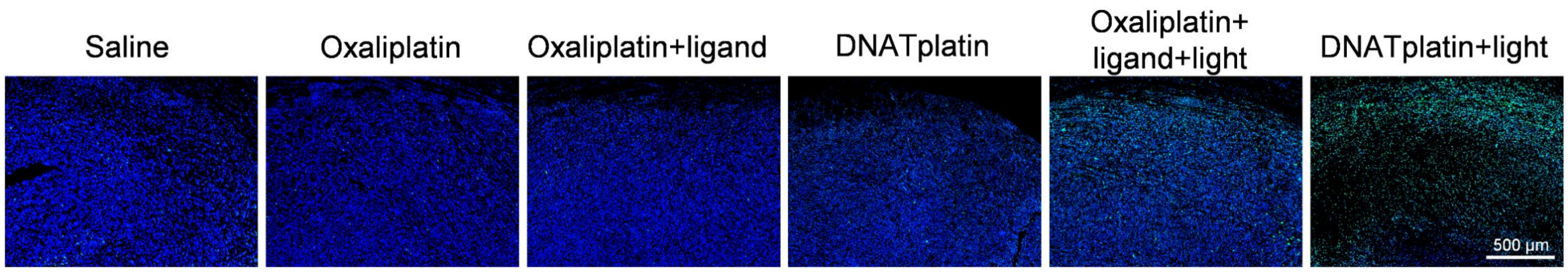

Figure S38. *γ*-H2AX staining of 4T1 tumor tissue sections from different treatment groups. Irradiation conditions for the animal experiments: λ_{ex} = 495 nm, 66 mW cm^{-2}.

Table S1. Solubility of the Pt(IV) complexes and oxaliplatin in water.

Chemical structures and names	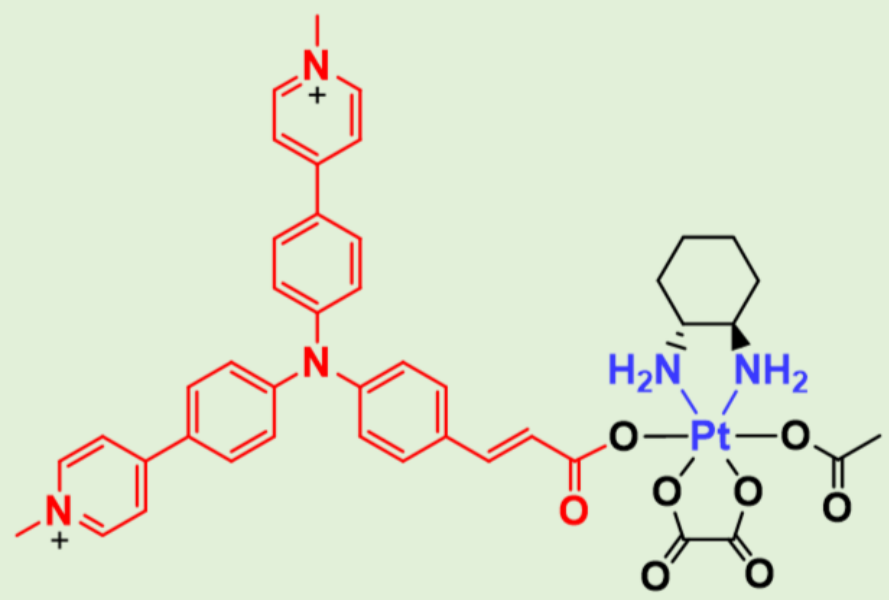 DNATplatin	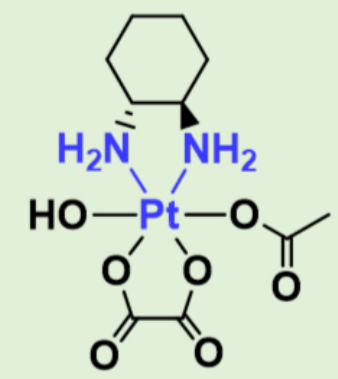 Ac-oxaPt(IV)-OH	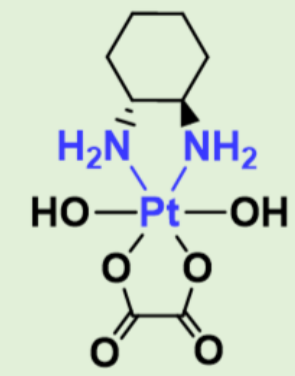 HO-oxaPt(IV)-OH	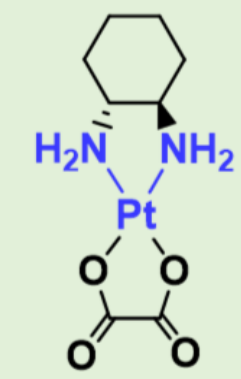 oxaliplatin
Solubility (mg Pt/mL H_2O)	18.82	17.96	1.14	2.12

Table S2. Comparative binding parameters of DNATplatin and free ligand toward different DNA topologies.

	DNA Topology	Fitting Model	Max Fold (I/I_0)	Hill Coeff.	K_D (nM)	K_a (M^{-1})
Ligand	dsDNA	Eq.1	~ 23-fold	N/A	22.98 ± 4.45	4.35×10^7
Ligand	G4DNA	Eq.2	~ 57-fold	2.85 ± 0.31	22.77 ± 0.93	4.39×10^7
DNATpaltin	dsDNA	Eq.1	~ 23-fold	N/A	20.24 ± 3.68	4.94×10^7
DNATpaltin	G4DNA	Eq.2	~ 58-fold	2.71 ± 0.10	21.33 ± 0.31	4.69×10^7

References:

(1) Qi, G.; Liu, X.; Li, H.; Qian, Y.; Liu, C.; Zhuang, J.; Shi, L.; Liu, B. A Dual-Mechanism Luminescent Antibiotic for Bacterial Infection Identification and Eradication. *Sci. Adv.* 2025, *11*, eadp9448.

(2) Xu, Z.; Lin, L.; Fu, B.; Huang, F. P.; Zhou, Q.; Zhu, G.; Wang, Z. Ligand Substitution Reactions Afford Oxaliplatin-Based Platinum(IV) Complexes Bearing Axial Alkoxido Ligands. *Inorg. Chem. Front.* 2023, *10*, 6058-6066.

(3) Barr, M. P.; Gray, S. G.; Hoffmann, A. C.; Hilger, R. A.; Thomale, J.; O'Flaherty, J. D.; Fennell, D. A.; Richard, D.; O'Leary, J. J.; O'Byrne, K. J. Generation and Characterisation of Cisplatin-Resistant Non-Small Cell Lung Cancer Cell Lines Displaying a Stem-Like Signature. *PLoS One* 2013, *8*, e54193.
